# KCTD1/KCTD15 complexes control ectodermal and neural crest cell functions, and their impairment causes aplasia cutis

**DOI:** 10.1172/JCI174138

**Published:** 2023-12-19

**Authors:** Jackelyn R. Raymundo, Hui Zhang, Giovanni Smaldone, Wenjuan Zhu, Kathleen E. Daly, Benjamin J. Glennon, Giovanni Pecoraro, Marco Salvatore, William A. Devine, Cecilia W. Lo, Luigi Vitagliano, Alexander G. Marneros

**Affiliations:** 1 Cutaneous Biology Research Center, Department of Dermatology, Massachusetts General Hospital, Harvard Medical School, Charlestown, Massachusetts, USA.; 2 IRCCS SYNLAB SDN, Naples, Italy.; 3 Stanford Cardiovascular Institute, Stanford University School of Medicine, Stanford, California, USA.; 4 Developmental Biology Department, John G. Rangos Sr. Research Center, University of Pittsburgh, Pittsburgh, Pennsylvania, USA.; 5 Institute of Biostructures and Bioimaging, Consiglio Nazionale delle Ricerche, Naples, Italy.

**Keywords:** Dermatology, Development, Embryonic development, Mouse models, Skin

## Abstract

Aplasia cutis congenita (ACC) is a congenital epidermal defect of the midline scalp and has been proposed to be due to a primary keratinocyte abnormality. Why it forms mainly at this anatomic site has remained a long-standing enigma. *KCTD1* mutations cause ACC, ectodermal abnormalities, and kidney fibrosis, whereas *KCTD15* mutations cause ACC and cardiac outflow tract abnormalities. Here, we found that KCTD1 and KCTD15 can form multimeric complexes and can compensate for each other’s loss and that disease mutations are dominant negative, resulting in lack of KCTD1/KCTD15 function. We demonstrated that KCTD15 is critical for cardiac outflow tract development, whereas KCTD1 regulates distal nephron function. Combined inactivation of KCTD1/KCTD15 in keratinocytes resulted in abnormal skin appendages but not in ACC. Instead, KCTD1/KCTD15 inactivation in neural crest cells resulted in ACC linked to midline skull defects, demonstrating that ACC is not caused by a primary defect in keratinocytes but is a secondary consequence of impaired cranial neural crest cells, giving rise to midline cranial suture cells that express keratinocyte-promoting growth factors. Our findings explain the clinical observations in patients with *KCTD1* versus *KCTD15* mutations, establish KCTD1/KCTD15 complexes as critical regulators of ectodermal and neural crest cell functions, and define ACC as a neurocristopathy.

## Abstract 2

<p>Loss of KCTD1/KCTD15 function in neural crest cells causes scalp aplasia cutis, whereas their loss in keratinocytes results in skin appendage defects.</p>

## Graphical abstract

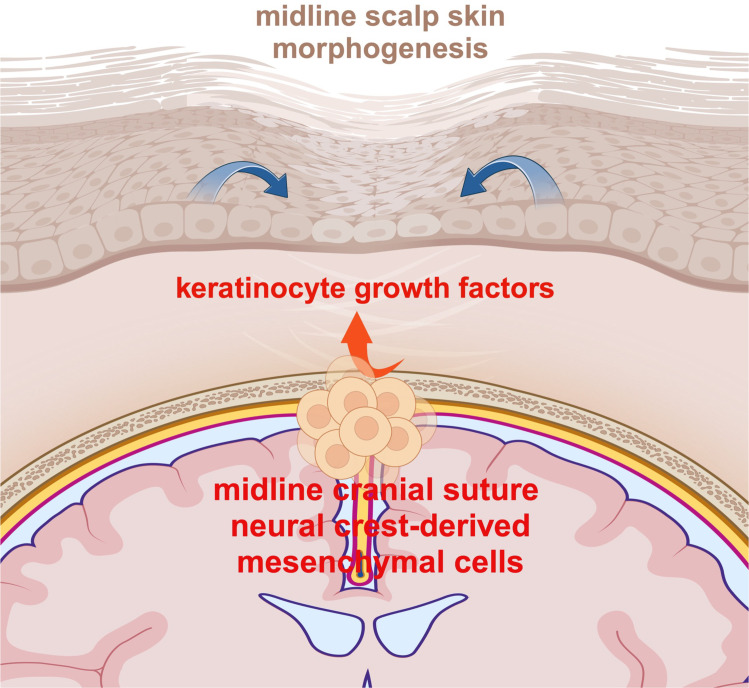

## Introduction

Aplasia cutis congenita (ACC) manifests with a congenital localized epidermal thinning (membranous ACC) or skin wound most commonly along the midline and vertex area of the scalp (1). Absence of skin at other anatomic sites and associated with various disorders has been reported as well, but these manifestations represent likely distinct clinical entities with a different pathogenesis (2). Since the description of scalp ACC centuries ago (3), it has remained a long-standing enigma why it occurs and, especially, why it is localized in most cases particularly at a defined anatomic site on the midline scalp without affecting other areas of the skin and without causing generalized skin and wound healing abnormalities in the adult. It has been postulated that ACC is the result of a primary defect in keratinocytes that results in their delayed migration or proliferation, leading to a skin closure defect of the rapidly expanding embryonic head where mechanical tension is particularly high (4–6). However, the molecular pathomechanisms and cellular origin of ACC remain unknown. Recently, missense mutations in the transcriptional regulators *KCTD1* and *KCTD15* have been linked to syndromes in which ACC occurs, in addition to various other clinical abnormalities (7, 8).

KCTD1 belongs to the family of BTB (bric-à-brac, tramtrack, broad complex) domain–containing proteins (9). A comprehensive understanding of the in vivo functions of KCTD1 is lacking. Heterozygous missense mutations in *KCTD1* lead to autosomal dominant scalp-ear-nipple (SEN) syndrome, which manifests primarily with scalp ACC, various additional ectodermal abnormalities (sparse hair, absence of incisors, anhidrosis, hypoplasia or absence of nipples/breasts), and facial dysmorphism, as well as progressive renal fibrosis (7, 10, 11). KCTD1 represses the transactivation of the transcription factor AP-2α through binding via its BTB domain, and *KCTD1* mutations in SEN syndrome abrogate the binding and inhibitory activity of KCTD1 on AP-2α, resulting in increased transcriptional activity of AP-2α (12–14). Notably, mutations in the *KCTD1* paralog *KCTD15* have been reported to cause ACC, frontonasal dysplasia, and cardiac outflow tract defects (8). Through which pathomechanisms *KCTD1* and *KCTD15* mutations cause these developmental defects remains unknown.

Here, we uncover the pathomechanistic basis and cellular origins of the clinical manifestations in patients with *KCTD1* or *KCTD15* mutations. Our data establish critical roles of KCTD1 and KCTD15 in keratinocytes for the formation of skin appendages (hairs, sebaceous glands, and eccrine sweat glands), whereas KCTD1 is critical for distal nephron function and KCTD15 is required for the proper development of the perimembranous ventricular septum and the aortic valve in the heart. Furthermore, we show that KCTD1 and KCTD15 control cranial neural crest cell (NCC) functions and that the combined loss of both KCTD1 and KCTD15 in NCCs results in ACC-like lesions of the midline scalp that are linked to midline cranial suture defects, establishing ACC as a neurocristopathy. Moreover, we show that KCTD1 and KCTD15 in NCCs are essential for the formation of nasal bones and incisors. Our findings demonstrate that ACC is not caused by a primary keratinocyte defect but instead is a secondary consequence of impaired NCCs that give rise to bones/sutures of the developing midline skull underlying ACC lesions and that normally express keratinocyte-promoting growth factors. Collectively, our data establish KCTD1 and KCTD15 as critical regulators of ectodermal and NCC functions, and their cell type–specific expression pattern explains the various clinical manifestations in patients with mutations in *KCTD1* or *KCTD15*.

## Results

### KCTD1 and KCTD15 can interact with each other in multimeric complexes.

**Figure 1:**
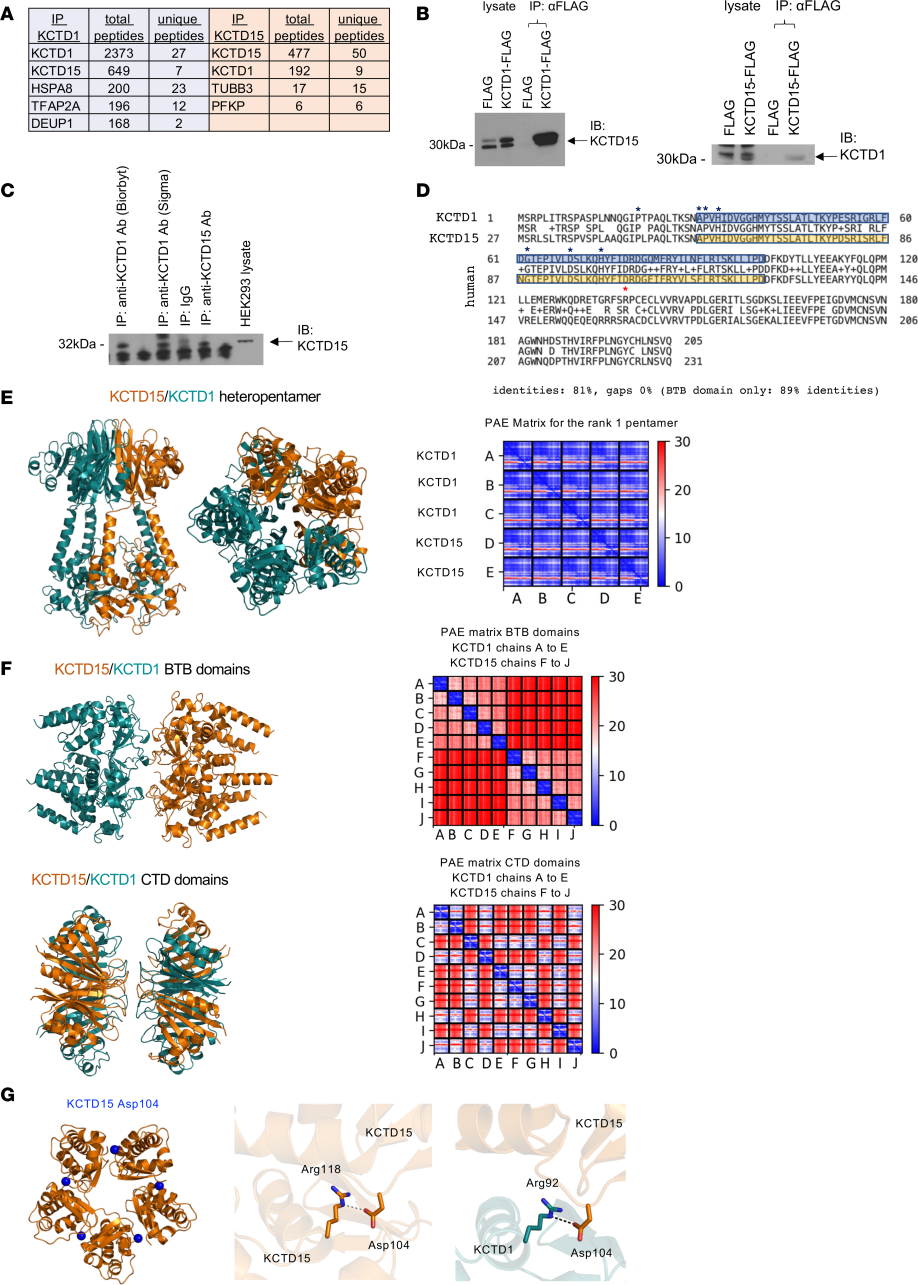
KCTD1 can form heteropentamers with KCTD15. (**A**) Proteins with the highest number of total peptides identified by mass spectrometry of immunoprecipitates (IP) with anti-FLAG antibodies using HEK293 cells that overexpress KCTD1-FLAG (IP KCTD1) or KCTD15-FLAG (IP KCTD15). (**B**) Left: IP of KCTD1-FLAG and immunoblotting for KCTD15. Right: IP of KCTD15-FLAG and immunoblotting for KCTD1. (**C**) Immunoblotting for KCTD15 of HEK293 cell lysates using KCTD1 or KCTD15 antibodies for IP. (**D**) Human KCTD1 and KCTD15 amino acid sequences show a high sequence identity, especially in the BTB domain (blue/yellow). Blue stars, amino acids affected by SEN syndrome mutations in *KCTD1*; red star, amino acid affected by *KCTD15* mutation. (**E**) Left: AlphaFold model of the KCTD1 (green)/KCTD15 (orange) heteropentamer. Right: Reliability of AlphaFold predictions assessed by the predicted aligned error (PAE) matrix, which provides a distance error for every pair of residues within the complex. Blue color, associated with low estimated errors of the distance of pairs of residues, is a strong indication of the global reliability of the model. These data support that KCTD1 and KCTD15 can form stable pentamers in any stoichiometry (here pentamer formation predicted to be stable between 3 KCTD1 and 2 KCTD15 monomers). (**F**) Top: Reliability of AlphaFold predictions for binding between 5 KCTD1 BTB domains (homomers) and 5 KCTD15 BTB domains (homomers) assessed by the PAE matrix, demonstrating that such an interaction would be unstable. Bottom: AlphaFold predictions for the association of 5 KCTD1 C-terminal domains and 5 KCTD15 C-terminal domains show that the tendency to form heteromers is greater than the tendency to form homomers (e.g., chain A forms non-red boxes with itself, and with chains B, D, H, and J, predicting heteromer formation rather than homomer formation). (**G**) Left: Asp104 (blue) at the intersubunit interface of the KCTD15 pentamer (BTB domains). Middle: Asp104 forms an intersubunit salt bridge with Arg118 between KCTD15 subunits. Right: Similar electrostatic interaction formed by Asp104 (KCTD15) and Arg92 (KCTD1), the residue that is equivalent to Arg118 of KCTD15, observed at the KCTD1-KCTD15 interface of the heteropentamer.

To identify proteins that interact with KCTD1, we overexpressed FLAG-tagged KCTD1 in HEK293 cells and performed immunoprecipitation (IP) followed by mass spectrometry. KCTD15 was the top-ranking protein identified as a KCTD1-interacting protein (Figure 1A). Additionally, TFAP2A (AP-2α) was found to interact with KCTD1 as well (Figure 1A). The identification of AP-2α and KCTD15 as binding proteins of KCTD1 is consistent with the previously published interaction between KCTD1 and AP-2α as well as KCTD15 and AP-2α (14, 15). Overexpression of FLAG-tagged KCTD15 in HEK293 cells and IP followed by mass spectrometry identified KCTD1 as the top-ranking KCTD15-interacting protein (Figure 1A). Co-IP experiments confirmed that KCTD1 and KCTD15 form protein complexes (Figure 1, B and C). The paralogs KCTD1 and KCTD15 belong to the family of BTB domain–containing proteins and have a high degree of sequence similarity (Figure 1D). X-ray crystallography showed that KCTD1 BTB domains (9) and the full-length proteins (Protein Data Bank 6S4L) form pentameric ring structures with plasticity in the KCTD1 rings, and modeling shows an almost identical structure for KCTD15 (Supplemental Figure 1A; supplemental material available online with this article; https://doi.org/10.1172/JCI174138DS1). This high sequence identity, together with the finding of the formation of protein complexes that contain KCTD1 and KCTD15, suggests that KCTD1 proteins form not only homo-oligomers but can also form hetero-oligomers with KCTD15 in vivo depending on the amounts of KCTD1 and KCTD15 present in a specific cell type. AlphaFold prediction suggests that KCTD1 and KCTD15 can form stable pentameric homomers and heteromers in any stoichiometry and that the tendency of the C-terminal domain to cross-associate into heteromers is higher than its tendency to self-associate into homomers (Figure 1, E and F, and Supplemental Figure 1B). In contrast, binding between homomeric pentamers is not predicted to be stable (Figure 1F). Thus, KCTD1 and KCTD15 are likely interchangeable within this pentameric structure depending on the abundance of KCTD1 versus KCTD15 in a cell type–dependent manner. Notably, a p.Asp104His mutation in the BTB domain of *KCTD15* has been reported to cause scalp ACC in a family in an autosomal dominant manner, similarly as observed in SEN syndrome families with *KCTD1* mutations (8). This p.Asp104His *KCTD15* mutation disrupts an intersubunit salt bridge with Arg118 of the adjacent KCTD15 or with the equivalent Arg92 of the KCTD1 subunit (Figure 1G), which would be predicted to destabilize pentameric assembly, similarly as reported for *KCTD1* mutations found in SEN syndrome (13). This *KCTD15* mutation and the *KCTD1* mutations in SEN syndrome affect amino acids that are conserved between these 2 highly homologous proteins (Figure 1D). Thus, the overlapping skin abnormalities in patients with mutations in *KCTD1* or *KCTD15* are consistent with a shared molecular function of these closely related paralogs in the developing skin and suggest that these missense mutations in *KCTD1* and *KCTD15* may act in a dominant-negative manner and inactivate KCTD1/KCTD15 complexes.

### Dominant-negative effects of KCTD1 mutations.

**Figure 2:**
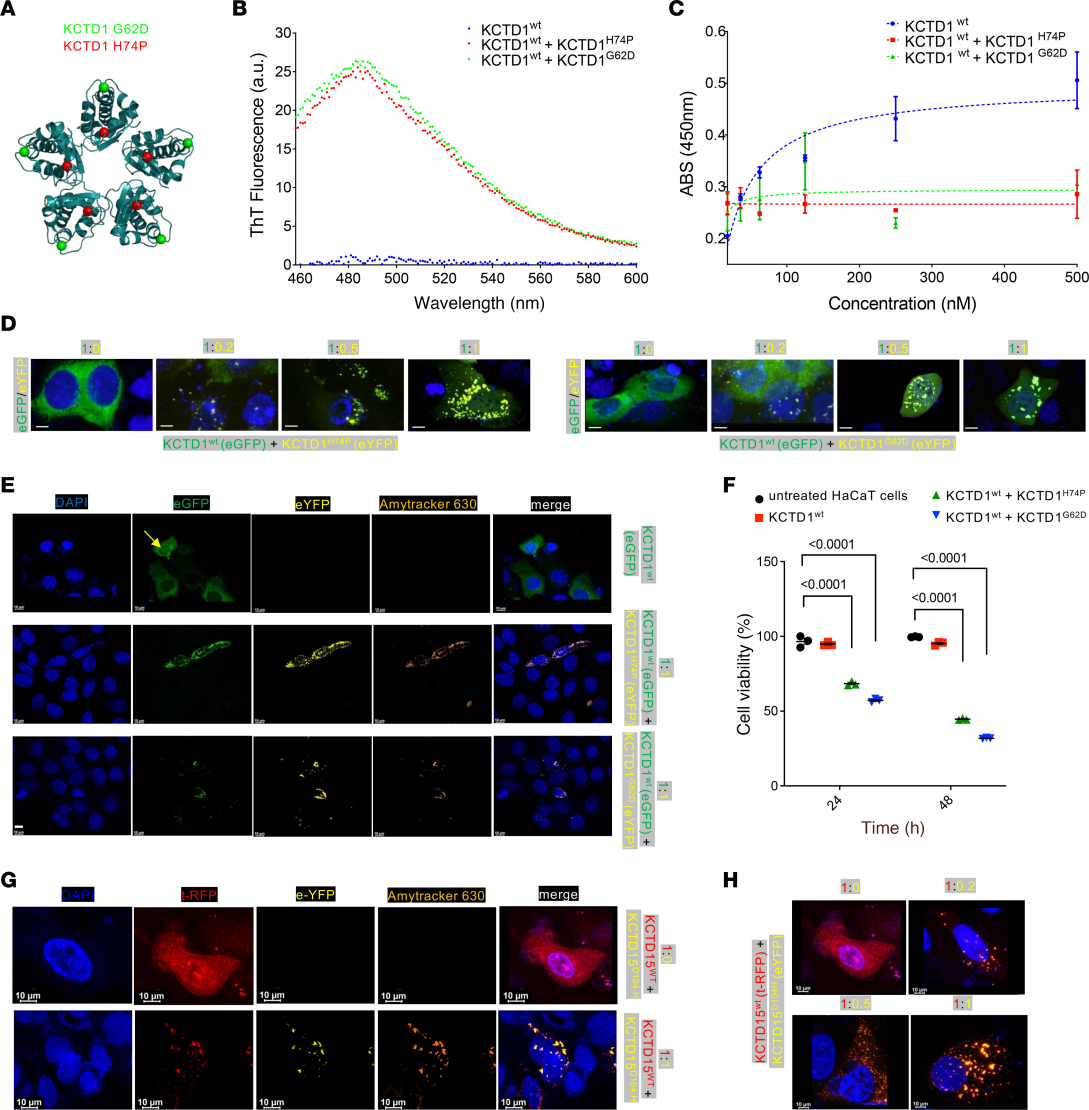
Dominant-negative effects of *KCTD1* mutations. (**A**) SEN syndrome mutation sites G62D (green) and H74P (red) in the BTB domain of KCTD1. (**B**) Thioflavin T (ThT) fluorescence assay shows amyloid-like aggregate formation in 5:1 mixtures of KCTD1^WT^ with KCTD1^H74P^ or KCTD1^G62D^ but not for KCTD1^WT^ without the mutant protein. Emission spectra obtained by addition of ThT solution to KCTD1^WT^ (blue circles), KCTD1^WT^ + KCTD1^H74P^ (red circles), and KCTD1^WT^ + KCTD1^G62D^ (green circles) proteins. Representative of 3 independent experiments. (**C**) Binding of KCTD1^WT^ (blue circles), KCTD1^WT^ + KCTD1^H74P^ (red squares), and KCTD1^WT^ + KCTD1^G62D^ (green triangles) to coated AP-2α determined by ELISA. KCTD1^WT^ was premixed with KCTD1^H74P^ or KCTD1^G62D^ mutants at a ratio of 5:1. Error bars represent SD (*n* = 2 per group). (**D**) With increasing ratio of KCTD1^H74P^ (eYFP, yellow) or KCTD1^G62D^ (eYFP, yellow) to KCTD1^WT^ (eGFP, green), KCTD1 protein aggregates increase in HaCaT cells. Scale bars: 10 μm. (**E**) KCTD1^WT^/KCTD1^H74P^ protein aggregates and KCTD1^WT^/KCTD1^G62D^ protein aggregates in HaCaT cells are detected by Amytracker-630. Arrow indicates nuclear KCTD1. Scale bars: 10 μm. (**F**) KCTD1^WT^/KCTD1^H74P^ protein aggregates and KCTD1^WT^/KCTD1^G62D^ aggregates (1:1 ratio) reduce cell viability in HaCaT cells. Mean ± SEM (*n* = 3 per group); *P* values (1-way ANOVA test with Dunnett’s multiple-comparison test). (**G**) KCTD15^D104H^ (eYFP, yellow) forms with KCTD15^WT^ (tRFP, red) amyloid-like aggregates (1:1 ratio) in HaCaT cells. Scale bars: 10 μm. (**H**) With increasing ratio of KCTD15^D104H^ (eYFP, yellow) to KCTD15^WT^ (tRFP, red) protein aggregates increase in HaCaT cells. Top left image is from **G**. Scale bars: 10 μm.

**Figure 3:**
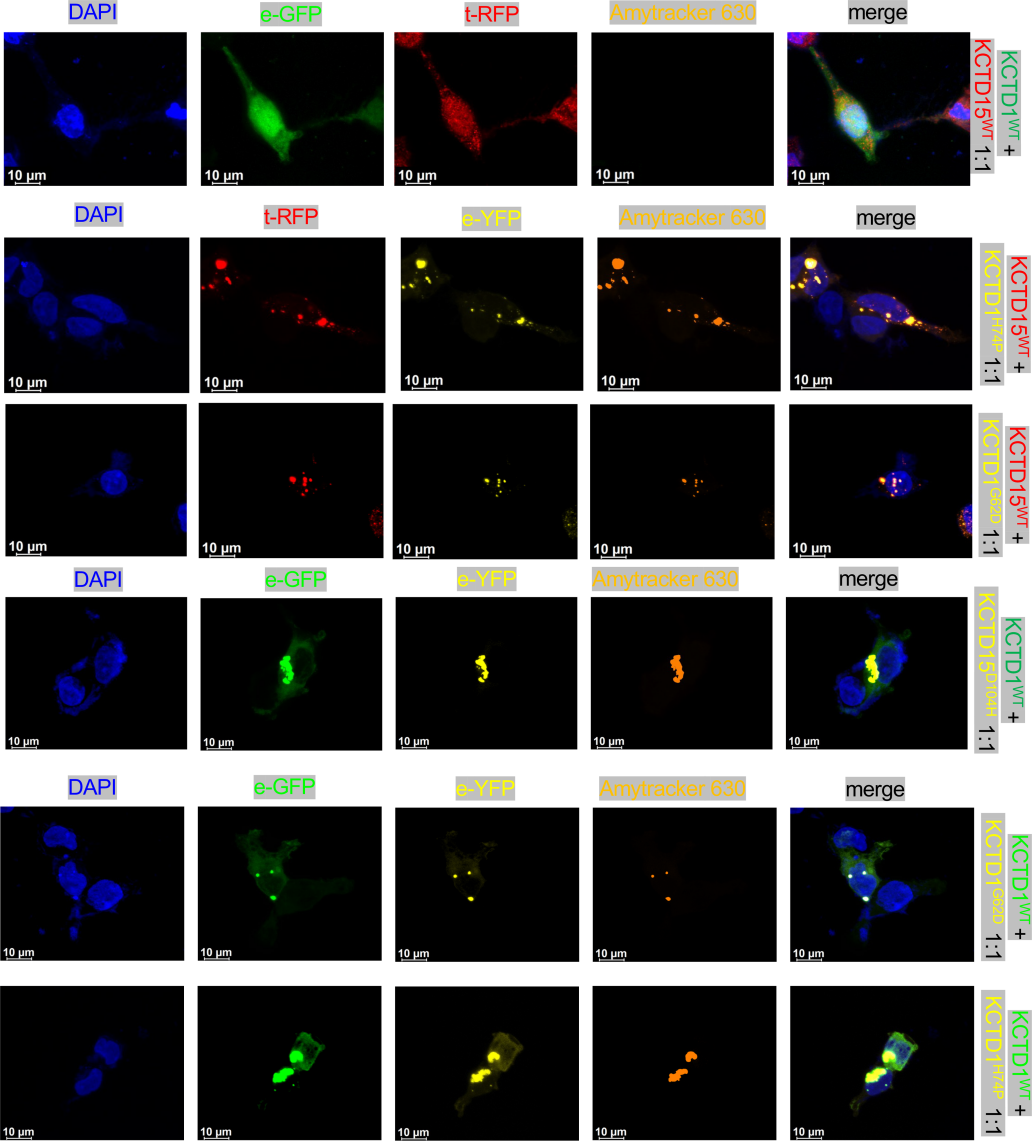
ACC-associated KCTD1 mutants sequester normal KCTD15 protein in amyloid-like aggregates and the ACC-associated KCTD15^D104H^ mutant sequesters normal KCTD1 protein in amyloid-like aggregates. KCTD1^WT^ (green) and KCTD15^WT^ (red) proteins show an overlapping expression pattern in HEK293 cells and do not form amyloid-like aggregates. In contrast, KCTD1^H74P^ and KCTD1^G62D^ mutants sequester both KCTD1^WT^ and KCTD15^WT^ in amyloid-like aggregates. The KCTD15^D104H^ mutant sequesters KCTD1^WT^ in amyloid-like aggregates. Scale bars: 10 μm.

SEN syndrome–causing *KCTD1* mutations favor an amyloid-like aggregation propensity of KCTD1 (13). We hypothesized that heterozygous SEN syndrome–causing *KCTD1* missense mutations exert a dominant-negative effect by causing an amyloid-like aggregation that sequesters not only mutant but also wild-type (WT) KCTD1 and/or KCTD15 that interact in oligomers, and thereby result in an inactivation of both KCTD1 and KCTD15. Thus, we assessed the amyloid-like aggregation propensity of WT KCTD1 (KCTD1^WT^) versus mixtures of KCTD1^WT^ with KCTD1^H74P^ or KCTD1^G62D^ (two SEN syndrome mutants [Figure 2A]) by performing thioflavin T fluorescence experiments (500 nM KCTD1^WT^ mixed with 100 nM KCTD1^H74P^ or KCTD1^G62D^). Amyloid-like aggregation was detected for the 2 mixtures but not for KCTD1^WT^ alone (Figure 2B). If a 1:5 ratio of mutant KCTD1 to KCTD1^WT^ leads to aggregation of KCTD1^WT^, then we would expect that this mixture would also prevent the observed binding of KCTD1 to AP-2α (a functional readout for KCTD1 activity, as KCTD1 binding to AP-2α is required for its inhibitory effect on the transcriptional activity of AP-2α; refs. 12, 13), whereas if only the mutant but not the WT KCTD1 would aggregate, then we would expect that the WT protein would still be able to bind to AP-2α. We found in KCTD1/AP-2α binding assays that whereas KCTD1^WT^ bound to AP-2α in a dose-dependent manner with submicromolar affinity (*K_D_* = 30 nM), the addition of KCTD1^H72P^ or KCTD1^G62D^ to KCTD1^WT^ completely prevented AP-2α binding despite the relative abundance of KCTD1^WT^ (Figure 2C). The combination of these 2 experiments suggests that SEN syndrome–causing *KCTD1* mutations exert their effects through a dominant-negative mechanism in which mutant KCTD1 proteins sequester normal KCTD1 or KCTD15 proteins that are part of their molecular complexes and, thereby, abrogate their functional activity. Consistent with this conclusion, we observe in keratinocyte cultures that KCTD1^WT^ overexpression does not result in cellular aggregates, whereas increasing ratios of KCTD1^H72P^ or KCTD1^G62D^ to KCTD1^WT^ lead to the formation of increasingly large intracellular aggregates that show intrinsic fluorescence (a feature of amyloid-like aggregates) (Figure 2D and Supplemental Figure 1C). These amyloid-like aggregates are detected by Amytracker, a fluorescent optotracer that labels protein aggregates with repetitive arrangement of β-sheets, identifying prefibrillar states of amyloids (Figure 2E and Supplemental Figure 1D). Similar results were also obtained in HEK293 and HeLa cells (Figure 3 and Supplemental Figures 2 and 3). The formation of amyloid-like KCTD1^WT^/KCTD1^H72P^ or KCTD1^WT^/KCTD1^G62D^ aggregates reduced cell viability of keratinocytes (Figure 2F). These aggregates were not found in the extracellular space. Similarly to the ACC-causing *KCTD1* mutants, the ACC-causing *KCTD15^D104H^* mutant resulted also in the sequestration of KCTD15^WT^ and the formation of amyloid-like aggregates (Figure 2, G and H). Moreover, while cotransfection of *KCTD1^WT^* with *KCTD15^WT^* resulted in subcellular colocalization without the formation of amyloid-like aggregates in HaCaT cells, HEK293 cells, and HeLa cells, the *KCTD15^D104H^* mutant sequestered *KCTD1^WT^* in amyloid-like aggregates and the *KCTD1^G62D^* and *KCTD1^H72P^* mutants sequestered *KCTD15^WT^* in amyloid-like aggregates (Figure 3 and Supplemental Figures 2 and 3). This finding is consistent with a dominant-negative effect of the ACC-associated *KCTD1* and *KCTD15* mutations that result in a loss-of-function effect of both KCTD1 and KCTD15 proteins.

KCTD1 and KCTD15 localize in cell lines in both the cytoplasmic and nuclear compartments, albeit the relative distribution differs between cell lines (Supplemental Figure 4, A and B, and Supplemental Videos 1 and 2). Immunolabeling shows that in both mouse and human epidermis KCTD1 and KCTD15 are mainly localized in nuclei of keratinocytes, consistent with their ability to inhibit the transcription factor AP-2α that shows nuclear localization (Supplemental Figure 4, C–E). Loss of AP-2α and AP-2β in keratinocytes does not prevent nuclear localization of KCTD1 and KCTD15 in epidermal keratinocytes in vivo and vice versa (Supplemental Figure 4, D and E).

### Expression pattern of Kctd1 and Kctd15.

**Figure 4:**
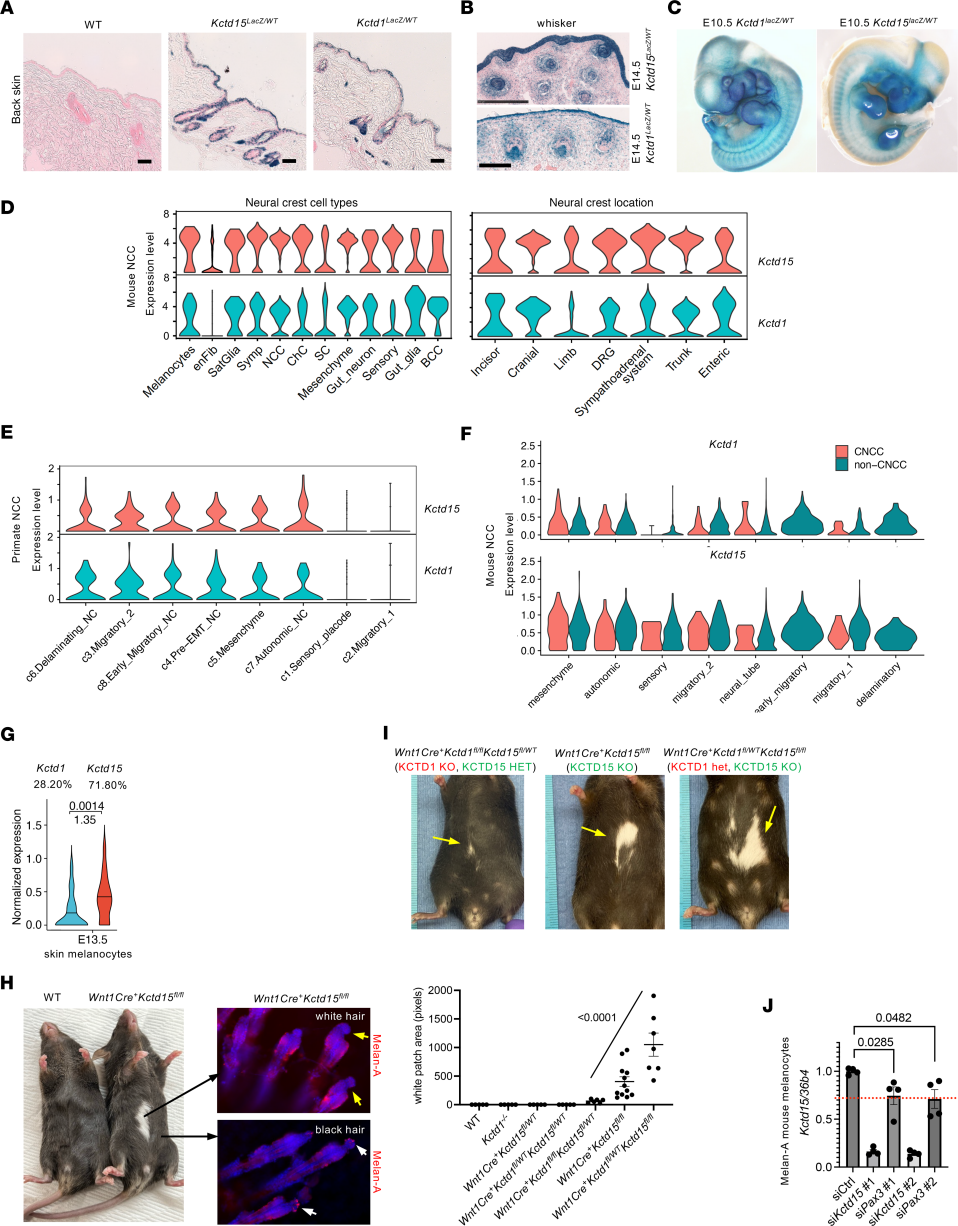
Expression pattern of *Kctd1* and *Kctd15*. (**A**) X-gal labeling of skin from adult *Kctd15^LacZ/WT^* and *Kctd1^LacZ/WT^* mice shows that *Kctd1* and *Kctd15* are expressed in epidermis and hair follicles. Scale bars: 250 μm. (**B**) *Kctd1* and *Kctd15* are expressed in developing epidermis and whiskers (E14.5 *Kctd15^LacZ/WT^* and *Kctd1^LacZ/WT^* mice). Scale bars: top, 250 μm; bottom, 100 μm. (**C**) X-gal staining of E10.5 *Kctd15^LacZ/WT^* and *Kctd1^LacZ/WT^* embryos shows labeling pattern similar to that of neural crest reporter mice (16). (**D**) *Kctd1* and *Kctd15* expression in mouse NCC populations (total 5,741 cells; ref. 18). BCC, boundary cap cells; ChC, chromaffin cells; DRG, dorsal root ganglia; enFib, endoneural fibroblasts; Sat, satellite; SC, Schwann cells; Symp, sympathetic neurons. (**E**) *KCTD1* and *KCTD15* expression in primate NCC populations (total 56,636 cells; ref. 22). (**F**) Comparison of expression of *Kctd1* and *Kctd15* in both cardiac NCC–derived (CNCC) and non–cardiac NCC–derived (non-CNCC) cells in mice between E8.5 and E10.5 (total 4,651 cells, including 260 CNCC and 4,391 non-CNCC; ref. 17). (**G**) *Kctd1* and *Kctd15* expression in melanocytes of developing mouse skin at E13.5 based on scRNA-Seq data (143 melanocytes in data set; ref. 48). Adjusted *P* value and log_2_(fold change) *Kctd15*/*Kctd1* are shown. Percentages indicate relative contribution to overall combined *Kctd1* and *Kctd15* transcript levels in these melanocytes. (**H**) Left: *Wnt1Cre^+^Kctd15^fl/fl^* mice develop a white belly patch due to lack of melanocytes. Right: Lack of Melan-A^+^ melanocytes in hair follicle whole mounts in white patches (yellow arrows), in contrast to presence of melanocytes in adjacent pigmented hair follicles (white arrows). Original magnification, ×20. (**I**) White belly patch size increases in this order: *Wnt1Cre^+^Kctd1^fl/fl^Kctd15^fl/WT^* mice < *Wnt1Cre^+^Kctd15^fl/fl^* mice < *Wnt1Cre^+^Kctd1^fl/WT^Kctd15^fl/fl^* mice. *Y* axis, pixel area of white patches (normalized to length scale). Mean ± SEM; *P* < 0.0001 (1-way ANOVA with test for linear trend among these 3 groups). (**J**) siRNA-mediated knockdown of *Kctd15* or *Pax3* in mouse melanocytes (Melan-A cells) inhibits expression of *Kctd15* (*Kctd15* transcript levels normalized to *36b4* and to control group treated with scrambled siRNA). Results shown for 2 different primer pairs for *Kctd15* and *Pax3*. *N* = 4 per group. Mean ± SEM; *P* values (Kruskal-Wallis test with Dunn’s multiple-comparison test).

To assess the cellular expression pattern of *Kctd1* and *Kctd15* in distinct tissues, we generated *Kctd1^lacZ/WT^* and *Kctd15^lacZ/WT^* reporter mice. We also analyzed single-cell RNA-Seq (scRNA-Seq) data sets from mice, humans, and primates at different developmental stages and in the adult. We found that, among other cell types, *Kctd1* and *Kctd15* are both expressed in keratinocytes of the epidermis, hair follicles, sweat glands, and teeth during development and in the adult, suggesting that KCTD1 and KCTD15 can compensate for each other’s loss in keratinocytes (Figure 4, A and B, and Supplemental Figures 5 and 6). An overlap in expression was also found in NCC populations, albeit *Kctd15* was more highly expressed than *Kctd1* in various NCC-derived cell subpopulations, such as in melanoblasts/melanocytes (Figure 4, C–F, and Supplemental Figures 6–8) (16–22). In contrast, in the distal convoluted tubule of the kidney, high expression was detected only for *Kctd1* but not for *Kctd15* (Supplemental Figure 9, A and B).

To provide in vivo evidence that KCTD1 and KCTD15 may have interchangeable and compensatory functions in a specific cell type where both are expressed and that the severity of a phenotype correlates to the degree of the combined loss of overall KCTD1/KCTD15 activity, we assessed the consequence of the loss of KCTD1 and KCTD15 in melanoblasts/melanocytes. NCC-derived melanocytes show higher expression of *Kctd15* than *Kctd1* in the developing skin (Figure 4G). Consistent with this difference, inactivation of KCTD15 in NCCs (*Wnt1Cre^+^Kctd15^fl/fl^* mice) resulted in a white belly patch, a loss-of-pigmentation phenotype with absence of melanocytes that is pathognomonic for an NCC-derived melanoblast migration defect (Figure 4H). In contrast, loss of KCTD1 (*Kctd1^–/–^* mice) or heterozygosity for both in NCCs (*Wnt1Cre^+^Kctd1^fl/WT^Kctd15^fl/WT^* mice) did not result in a pigmentation defect. This suggests that, because of the higher expression of *Kctd15* over *Kctd1* in melanocytes, loss of KCTD15 cannot be compensated for by KCTD1, whereas loss of KCTD1 can be compensated for by KCTD15. *Wnt1Cre^+^Kctd1^fl/fl^Kctd15^fl/WT^* (*Kctd1*-KO, *Kctd15*-heterozygous) mice developed smaller white patches than *Wnt1Cre^+^Kctd15^fl/fl^* (*Kctd15*-KO) mice, whereas *Wnt1Cre^+^Kctd1^fl/WT^Kctd15^fl/fl^* (*Kctd1*-heterozygous, *Kctd15*-KO) mice developed larger white patches than *Wnt1Cre^+^Kctd15^fl/fl^* (*Kctd15*-KO) mice (Figure 4I). This demonstrates that if heterozygosity for *Kctd15* in NCCs is present, KCTD1 can compensate for the reduced KCTD15 levels and prevent the pigmentation defect, whereas if KCTD1 is lacking, this compensation is not present and small white belly patches form. Thus, the size of the pigmentation defect correlates with the extent of the loss of overall KCTD1/KCTD15 activity, consistent with interchangeable functions and compensation mechanisms between KCTD1 and KCTD15 in vivo.

The pigmentation abnormalities in *Wnt1Cre^+^Kctd15^fl/fl^* mice resemble those in *Wnt1Cre^+^Pax3^fl/fl^* mice (23), PAX3 being a key regulator of NCCs. Knockdown of *Pax3* in melanoblasts resulted in reduced expression of *Kctd15* (Figure 4J), indicating that KCTD15 may function downstream of PAX3 in these cells during development.

### Compensation between KCTD1 and KCTD15 in keratinocytes.

To assess possible compensation effects between KCTD1 and KCTD15 in keratinocytes and to test whether ACC is a consequence of a primary keratinocyte defect, we inactivated both *Kctd1* and *Kctd15* selectively in keratinocytes (using *K14Cre* mice). Consistent with an interchangeable function between KCTD1 and KCTD15 in keratinocytes, neither *K14Cre^+^Kctd1^fl/fl^* mice nor *K14Cre^+^Kctd15^fl/fl^* mice showed an apparent skin or hair phenotype, whereas inactivation of both *Kctd1* and *Kctd15* in keratinocytes (*K14Cre^+^Kctd1^fl/fl^Kctd15^fl/fl^* mice) resulted in skin and hair abnormalities that phenocopied the sparse hair and anhidrosis clinical findings in SEN syndrome patients with *KCTD1* mutations. This observation is consistent with a dominant-negative effect of these *KCTD1* mutations that result in a combined loss of KCTD1/KCTD15 function. However, no ACC-like lesions were observed in *K14Cre^+^Kctd1^fl/fl^Kctd15^fl/fl^* mice, suggesting that ACC occurs due to a primary loss of KCTD1/KCTD15 function in a cell type other than keratinocytes.

**Figure 5:**
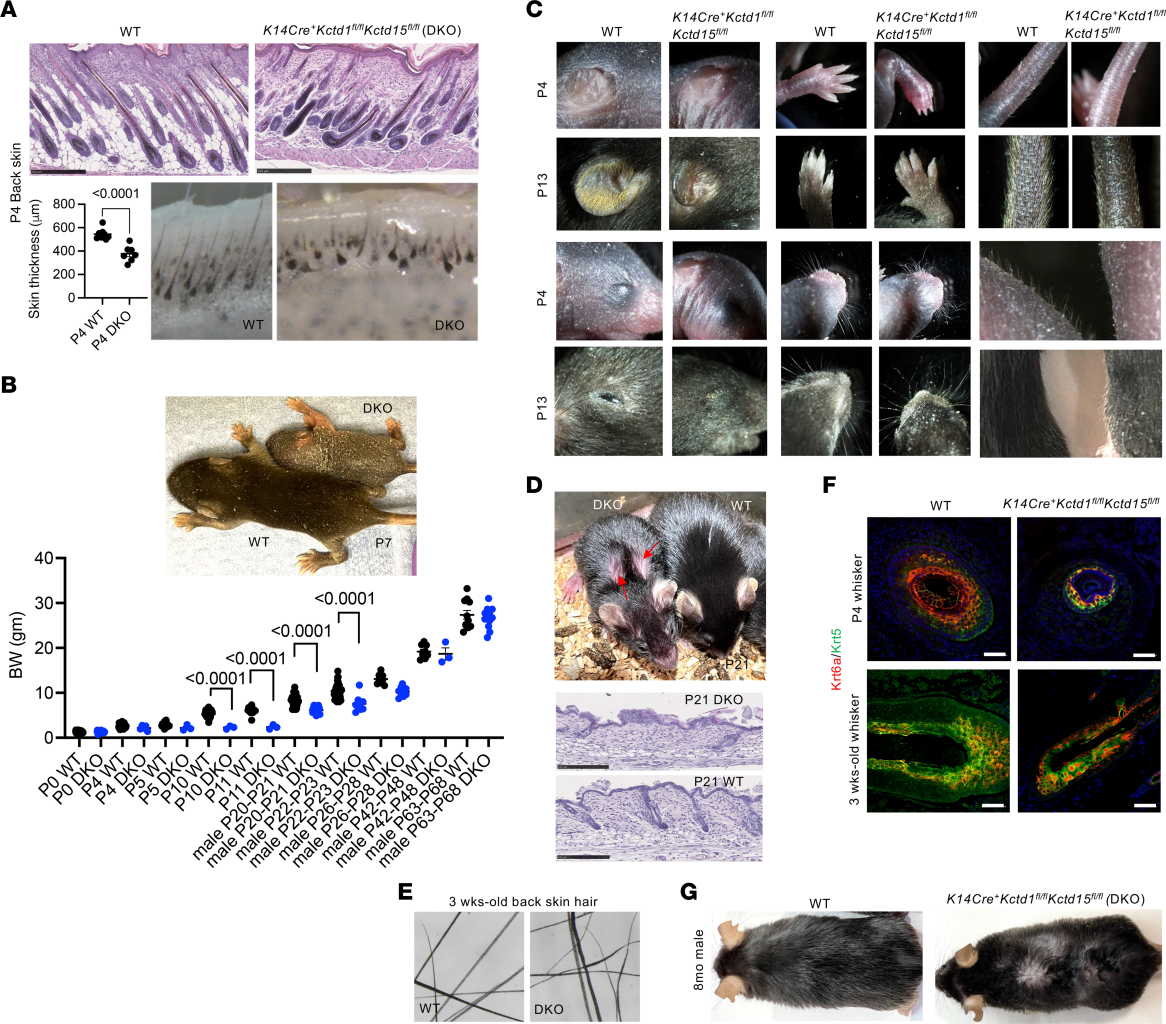
KCTD1/KCTD15 complexes in keratinocytes are critical regulators of skin and hair follicles. (**A**) Hair/skin abnormalities in P4 *K14Cre^+^Kctd1^fl/fl^Kctd15^fl/fl^* mice (DKO). P4 DKO skin is thinner and shows abnormal and shorter hair follicles. Graph shows skin thickness in micrometers (*n* = 7–8 mice per group; mean ± SEM; *P* values, 2-tailed *t* test). Scale bars: 250 μm. (**B**) Delay in hair growth and postnatal growth retardation in DKO mice at P7. *K14Cre^+^Kctd1^fl/fl^Kctd15^fl/fl^* mice (DKO) (blue dots) are born with similar body weight (BW) compared to their controls (black dots) but develop a postnatal growth retardation. Mean ± SEM; *P* values (2-tailed *t* test). (**C**) P4 and P13 *K14Cre^+^Kctd1^fl/fl^Kctd15^fl/fl^* mice versus WT littermates. A delay in opening of the eyelids, interdigital web space formation, and hair growth are observed in *K14Cre^+^Kctd1^fl/fl^Kctd15^fl/fl^* mice, as well as curly whiskers. (**D**) At P21, DKO mice have a sparser fur coat and patches with diminished hair on the upper back (red arrows). Scale bars: 250 μm. (**E**) Structural hair shaft abnormalities in 3-week-old DKO mice. (**F**) Whisker abnormalities in *K14Cre^+^Kctd1^fl/fl^Kctd15^fl/fl^* mice. Scale bars: 50 μm. (**G**) Generalized sparse hair in adult DKO mice at 8 months of age.

**Figure 6:**
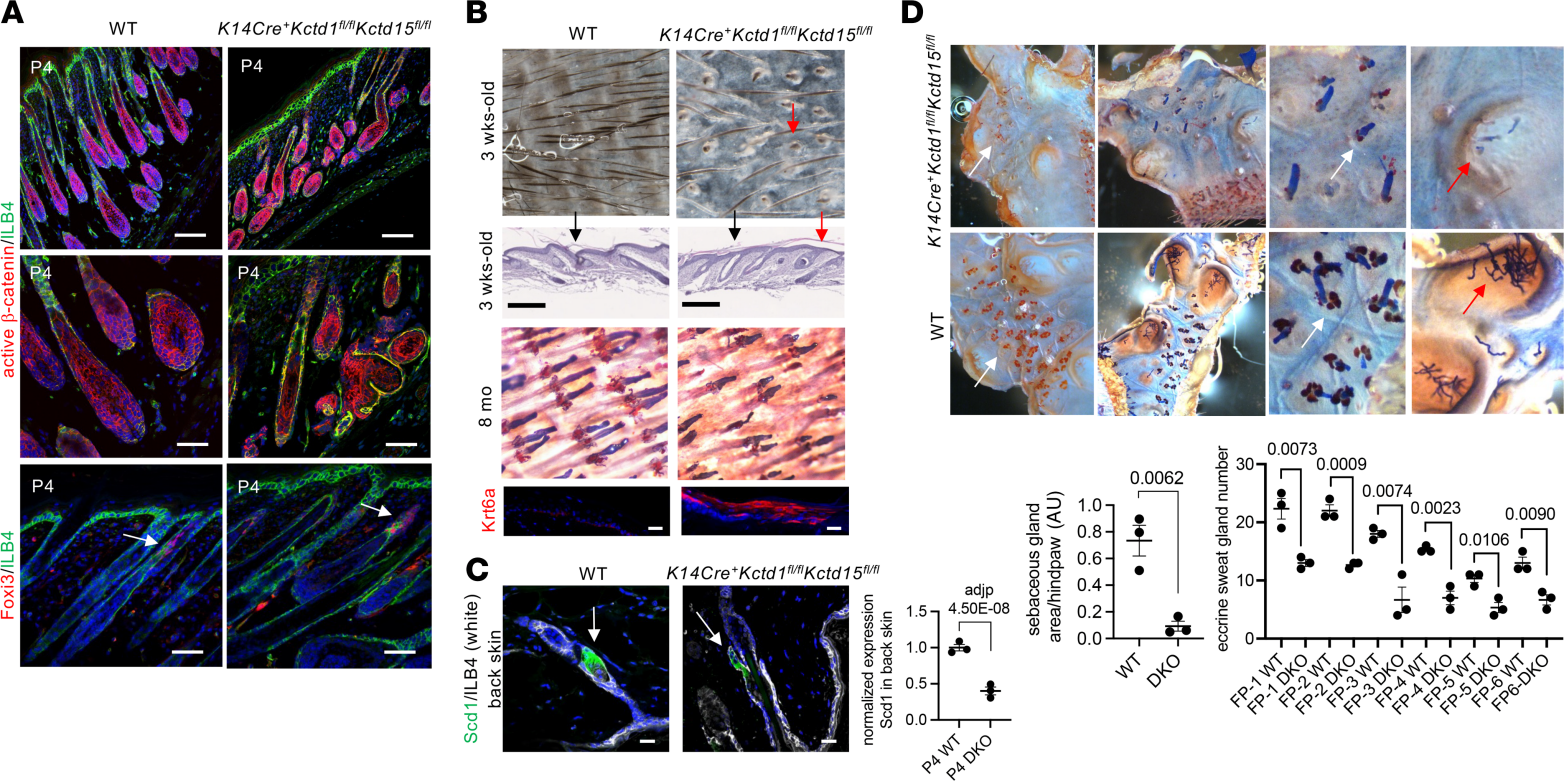
KCTD1/KCTD15 complexes in keratinocytes are required for the proper formation of skin appendages. (**A**) Immunolabeling for active β-catenin of P4 back skin in *K14Cre^+^Kctd1^fl/fl^Kctd15^fl/fl^* mice and WT mice shows abnormal and shorter hair follicles in the mutant mice. Foxi3 is localized at the isthmus/infundibulum junction of hair follicles at P4 (arrows). ILB4, isolectin B4. Scale bars: 100 μm (top); 50 μm (middle and bottom). (**B**) Top 2 rows: Reduced hairs and abnormal hair follicles (red arrows) with flattened scale/interscale junctions (black arrows) in 3-week-old tail skin of *K14Cre^+^Kctd1^fl/fl^Kctd15^fl/fl^* mice. Scale bars: 250 μm. Bottom 2 rows: Abnormal hairs and reduced sebaceous glands in tail skins of 8-month-old *K14Cre^+^Kctd1^fl/fl^Kctd15^fl/fl^* mice, with increased Krt6a in the interfollicular epidermis. Scale bars: 10 μm. (**C**) Left: SCD1 immunolabeling of back skin of adult *K14Cre^+^Kctd1^fl/fl^Kctd15^fl/fl^* mice (DKO) and littermate controls. Scale bars, 20 μm. Right: RNA-Seq values (*n* = 3 per group) for *Scd1* expression in P4 back skin of DKO versus control mice. Mean ± SEM; adjusted *P* value. (**D**) Oil Red O and Nile blue staining of footpad skin from adult *K14Cre^+^Kctd1^fl/fl^Kctd15^fl/fl^* mice shows diminished sebaceous glands (orange, white arrows) and loss of eccrine sweat glands (red arrows) compared with WT controls. Quantification of Oil Red O^+^ hind paw area (sebaceous glands) in 7-week-old male *K14Cre^+^Kctd1^fl/fl^Kctd15^fl/fl^* mice (DKO) and littermate controls. Eccrine gland duct numbers for each footpad (FP1 to FP6) of these mice are shown. Mean ± SEM; *P* values (2-tailed *t* test; *n* = 3 mice per group).

*K14Cre^+^Kctd1^fl/fl^Kctd15^fl/fl^* mice developed hair follicle abnormalities and a postnatal growth retardation, associated with reduced skin thickness and a delay in hair growth and skin maturation (e.g., a delay in the opening of the eyelids and the formation of interdigital web spaces), a generalized sparseness of the fur coat, and structural hair abnormalities, as well as abnormal whisker hair follicles with curly whiskers (Figure 5, A–G, Figure 6, A and B, and Supplemental Figure 10, A–E). Sebaceous glands, which still expressed their differentiation marker SCD1, were markedly diminished (Figure 6, C and D, and Supplemental Figure 10F). Eccrine sweat gland numbers in footpads were strongly reduced in *K14Cre^+^Kctd1^fl/fl^Kctd15^fl/fl^* mice, consistent with the reported anhidrosis in SEN syndrome patients (Figure 6D). Thus, KCTD1/KCTD15 complexes are key regulators of the formation of skin appendages (hair follicles, sebaceous glands, eccrine sweat glands).

**Figure 7:**
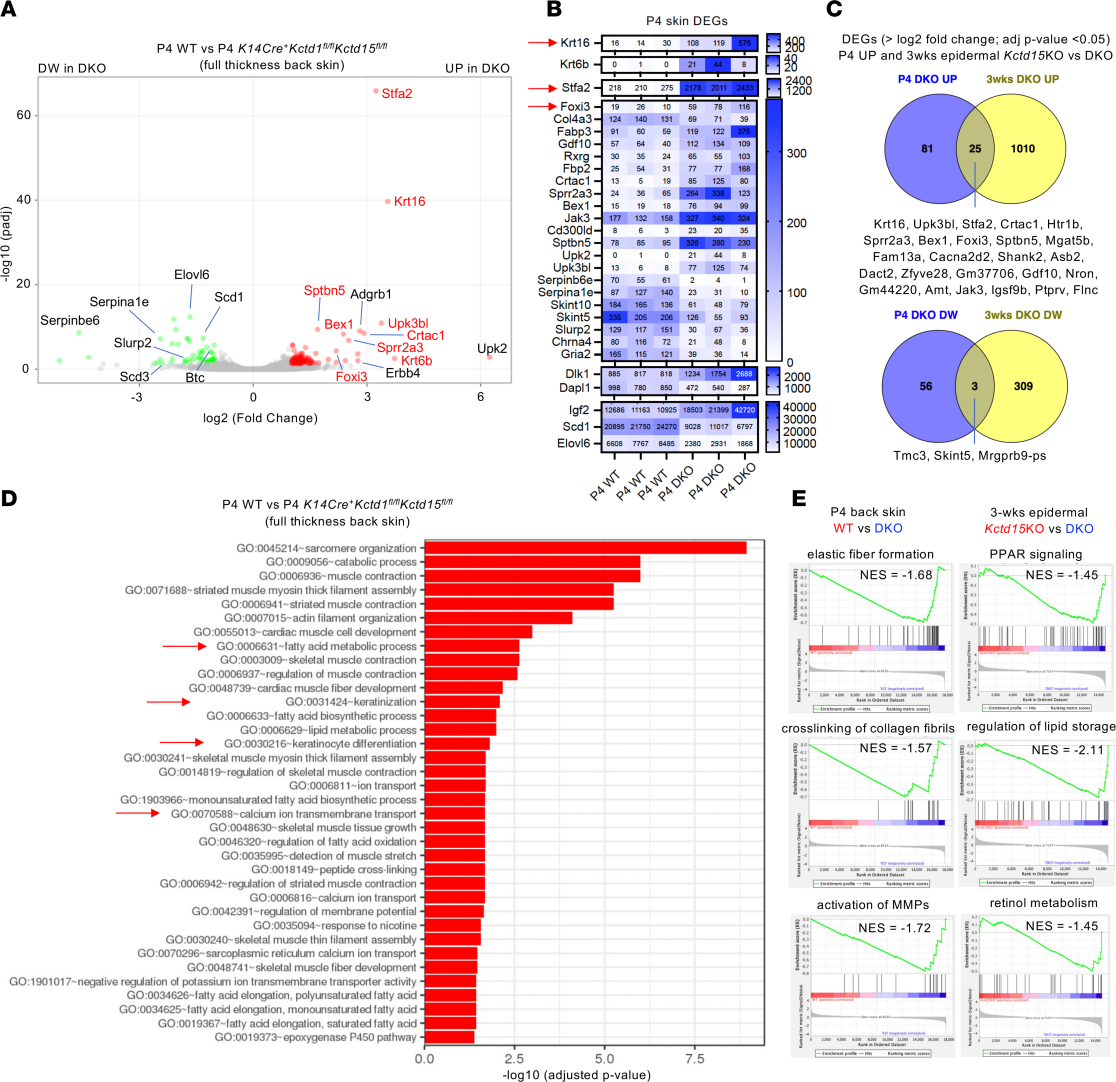
Transcriptomic characterization of skin abnormalities in *K14Cre^+^Kctd1^fl/fl^Kctd15^fl/fl^* mice. (**A**) Volcano plot of RNA-Seq data from back skin of P4 control mice compared with *K14Cre^+^Kctd1^fl/fl^Kctd15^fl/fl^* mice. (**B**) Heatmap shows expression of selected differentially expressed genes (DEGs) in the back skin of P4 control mice compared with *K14Cre^+^Kctd1^fl/fl^Kctd15^fl/fl^* mice. Upregulated DEGs in the skin of *K14Cre^+^Kctd1^fl/fl^Kctd15^fl/fl^* mice include *Krt16*, *Foxi3*, and *Stfa2* (arrows). (**C**) Venn diagrams show DEGs in P4 back skin or 3-week-old back skin epidermis of *K14Cre^+^Kctd1^fl/fl^Kctd15^fl/fl^* mice (compared with *K14Cre^+^Kctd15^fl/fl^* mice that did not have hair/skin abnormalities). Among DEGs upregulated at both time points examined are Foxi3, Krt16, and Stfa2. (**D**) Gene Ontology (GO) enrichment analysis of P4 back skin RNA-Seq data from *K14Cre^+^Kctd1^fl/fl^Kctd15^fl/fl^* mice versus control littermates. (**E**) GSEA shows downregulated pathways in the back skin of P4 *K14Cre^+^Kctd1^fl/fl^Kctd15^fl/fl^* mice, as well as in the back skin epidermis of 3-week-old *K14Cre^+^Kctd1^fl/fl^Kctd15^fl/fl^* mice compared with *K14Cre^+^Kctd15^fl/fl^* mice. NES, normalized enrichment score.

RNA-Seq of full-thickness skin from the back of P4 *K14Cre^+^Kctd1^fl/fl^Kctd15^fl/fl^* mice versus WT littermates showed a strong increase in the expression of the alarmin keratins *Krt6* and *Krt16* and the hair follicle stem cell regulator *Foxi3*, as well as *Stfa2*, which plays a role in epidermal differentiation (Figure 7, A–C). Genes downregulated in P4 skin of *K14Cre^+^Kctd1^fl/fl^Kctd15^fl/fl^* mice included regulators of fatty acid metabolism (*Elovl6*, *Scd1*), which are expressed in the skin mainly in sebaceous glands (Figure 7, A and B, and Supplemental Figure 10F). This supports our finding of reduced sebaceous glands in *K14Cre^+^Kctd1^fl/fl^Kctd15^fl/fl^* mice but not of a sebocyte differentiation block, as immunolabeling for the sebocyte differentiation marker SCD1 was observed in smaller sebaceous glands of these mice (Figure 6C). Gene Ontology analysis of the differentially expressed genes showed a link to changes in keratinocyte differentiation, fatty acid metabolism, and calcium ion transport processes (Figure 7D). Gene set enrichment analysis (GSEA) provided evidence for abnormal extracellular matrix/keratinocyte interactions in the mutant skin as well (downregulation of genes linked to elastic fiber formation, cross-linking of collagen fibrils, and activation of matrix metalloproteinases) (Figure 7E). We also found upregulation of *Foxi3*, *Krt16*, and *Stfa2* in epidermis derived from P22 *K14Cre^+^Kctd1^fl/fl^Kctd15^fl/fl^* mice (telogen/anagen transition phase), indicating a key role of these genes in keratinocytes for the observed abnormalities (Figure 7C). Notably, expression of *Kctd1* and *Kctd15* in keratinocytes was not dependent on AP-2α or AP-2β and vice versa (Supplemental Figure 10G). GSEA linked gene expression changes in the epidermis of P22 *K14Cre^+^Kctd1^fl/fl^Kctd15*^fl/fl^ mice to alterations in PPAR signaling, retinol metabolism, and regulation of lipid storage (Figure 7E). Overall, inactivation of *Kctd1* and *Kctd15* in keratinocytes provides evidence for a critical role of KCTD1/KCTD15 complexes for the proper formation of hair follicles, sebaceous glands, and eccrine sweat glands. Heterozygosity for the genes for AP-2α, AP-2α/AP-2β, or β-catenin in keratinocytes did not rescue these skin abnormalities in *K14Cre^+^Kctd1^fl/fl^Kctd15*^fl/fl^ mice (data not shown), suggesting that hyperactivation of AP-2 or β-catenin pathway activity due to loss of KCTD1 and KCTD15, which both inhibit these pathways in vitro, is likely not critical for the manifestation of these skin appendage defects.

### ACC is a neurocristopathy.

**Figure 8:**
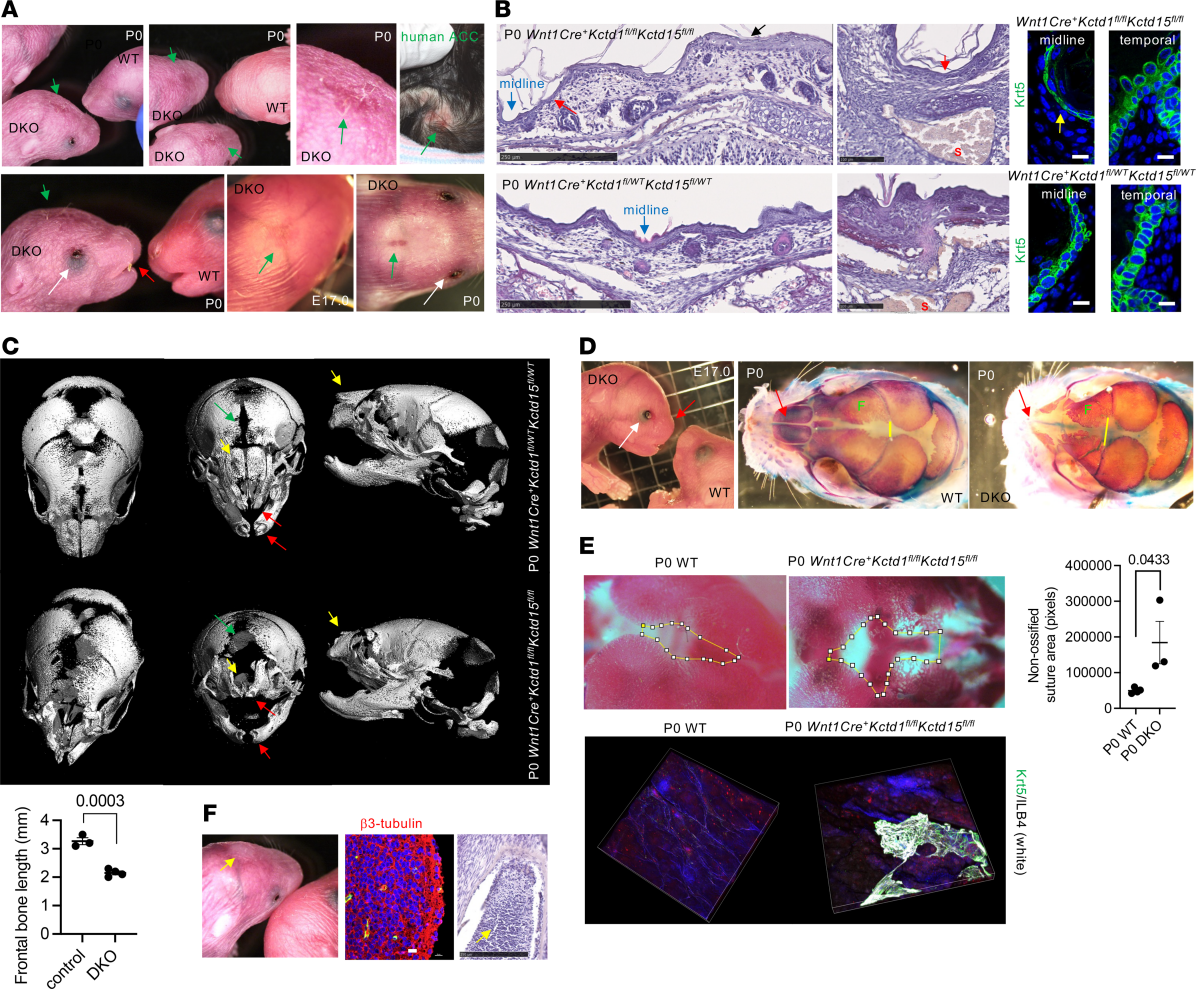
ACC is a neurocristopathy caused by loss of KCTD1/KCTD15 function in NCCs. (**A**) Newborn *Wnt1Cre^+^Kctd1^fl/fl^Kctd15^fl/fl^* mice show thin/eroded epidermis of the midline scalp overlying the interfrontal or sagittal sutures (green arrows), open eyelids (white arrows), and diminished nasal structures (red arrow). Top right: Newborn child with scalp ACC (arrow) (49). (**B**) H&Es show thinned epidermis (red arrows) with a flat Krt5^+^ basal layer (yellow arrow) overlying the interfrontal suture (midline, blue arrows) in P0 *Wnt1Cre^+^Kctd1^fl/fl^Kctd15^fl/fl^* mice, whereas the adjacent more temporal skin has a normal thickness (black arrow). This difference is not observed in P0 *Wnt1Cre^+^Kctd1^fl/WT^Kctd15^fl/WT^* mice. S, sagittal sinus. Scale bars: left, 250 μm; middle, 100 μm; right, 10 μm. (**C**) μCT images of P0 *Wnt1Cre^+^Kctd1^fl/fl^Kctd15^fl/fl^* mice (DKO) and *Wnt1Cre^+^Kctd1^fl/WT^Kctd15^fl/WT^* littermate mice show loss of incisors (red arrows), loss of nasal bones (yellow arrows), and delayed frontal bone ossification along the interfrontal suture (green arrows) in *Wnt1Cre^+^Kctd1^fl/fl^Kctd15^fl/fl^* mice. Graph shows frontal bone length in P0 control and DKO mice in millimeters (*n* = 3–4 mice per group; mean ± SEM; *P* value, 2-tailed unpaired *t* test). (**D**) Open eyes and flat nasal structures in E17.0 *Wnt1Cre^+^Kctd1^fl/fl^Kctd15^fl/fl^* mice (DKO). Skeletal preps of a P0 *Wnt1Cre^+^Kctd1^fl/fl^Kctd15^fl/fl^* mouse (DKO) and a littermate WT control show loss of nasal bones in the DKO (red arrows), shortened frontal bones (F), and delayed ossification of the interfrontal suture (yellow line shows increased distance between frontal bones at the site where the interfrontal suture crosses with the coronal suture in DKO mice). (**E**) Top: P0 *Wnt1Cre^+^Kctd1^fl/fl^Kctd15^fl/fl^* mice (DKO) show diminished ossification along the interfrontal and sagittal sutures and expanded non-ossified area at that site (outlined in yellow) compared with littermate controls (*n* = 3–4 mice per group; mean ± SEM; *P* values, 2-tailed unpaired *t* test). Bottom: Whole-mount immunolabeling of scalp skin shows demarcation of ACC-like region in a P0 DKO (Krt5 and ILB4 labeling). (**F**) Midline scalp mass (arrow) in a P0 *Wnt1Cre^+^Kctd1^fl/fl^Kctd15^fl/fl^* mouse shows β_3_-tubulin^+^ heterotopic neuronal tissue. Scale bars: 10 μm (immunolabeling); 250 μm (H&E).

As the combined loss of KCTD1 and KCTD15 in keratinocytes did not result in ACC, we hypothesized that ACC may be a secondary consequence of a primary defect in the underlying midline bones/sutures/mesenchymal cells of the developing skull. This hypothesis is based on the observation that most cases of membranous ACC, which is characterized by a thin epidermis of the midline scalp skin, form at sites overlying the midline cranial sutures and many of those have associated underlying bone/suture defects and heterotopic meningeal/brain tissue in the overlying skin (1). Notably, the midline cranial sutures (interfrontal and sagittal), as well as several midline skull bones (including nasal, frontal, and interparietal bones), are derived from NCCs (24). Thus, ACC forms at sites where the underlying bone/suture structures are derived from NCC populations. Based on the observed expression of *Kctd1* and *Kctd15* in cranial NCCs (Figure 4D), we tested whether KCTD1/KCTD15 complexes are critical regulators of NCC-derived bone/suture structures and whether their inactivation in NCCs results in skull defects and ACC. Indeed, inactivation of KCTD1/KCTD15 complexes in NCCs (*Wnt1Cre^+^Kctd1^fl/fl^Kctd15^fl/fl^* mice) resulted in congenital bone/suture defects of NCC-derived structures of the midline skull associated with overlying membranous ACC-like skin defects with epidermal thinning (Figure 8, A–E). These defects were not observed in mice lacking only KCTD1 or only KCTD15 in NCCs. Micro-CT (μCT) imaging and skeletal preparations demonstrated in newborn (P0) *Wnt1Cre^+^Kctd1^fl/fl^Kctd15^fl/fl^* mice a delayed ossification of shortened frontal bones along the interfrontal suture and much extended, abnormal midline cranial sutures with a receded osteogenic front (Figure 8, C–E). The ACC-like lesions occurred atop these abnormal interfrontal or sagittal sutures and had a flattened epidermis with a thin Krt5^+^ basal layer (Figure 8, B–E). Notably, a cutaneous forehead mass consisting of heterotopic neuronal tissue was observed in *Wnt1Cre^+^Kctd1^fl/fl^Kctd15^fl/fl^* mice as well (Figure 8F), consistent with an abnormal closure of bony structures in the skull during development and similar to heterotopic brain tissue observed at skin sites adjacent to membranous ACC lesions in patients.

**Figure 9:**
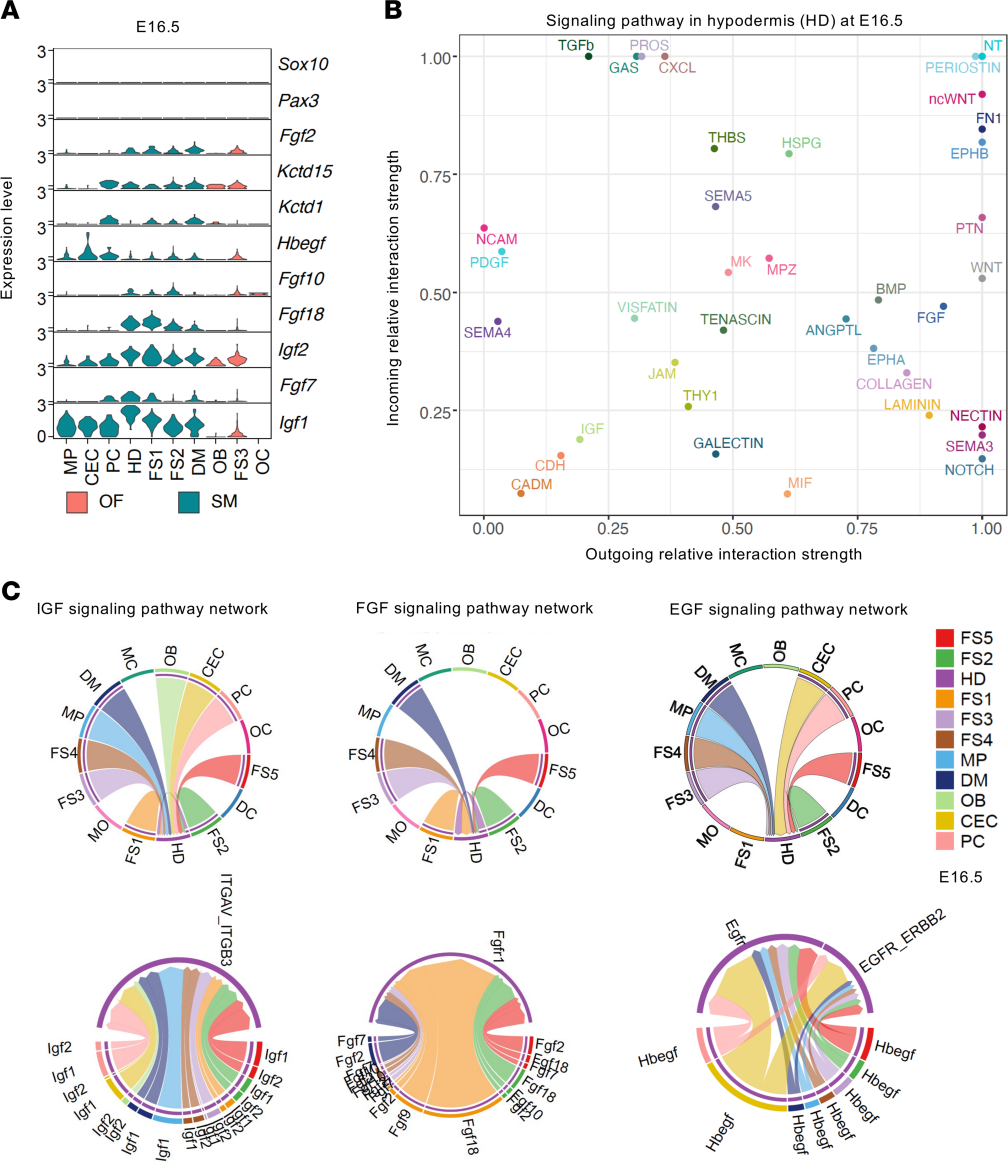
NCC-derived interfrontal suture mesenchymal cells express keratinocyte growth factors at E16.5. (**A**) Expression of *Kctd1*, *Kctd15*, keratinocyte-promoting growth factors (*Igf1*, *Igf2*, *Fgf2*, *Fgf7*, *Fgf10*, *Fgf18*, *Hbegf*), and NCC markers (*Pax3*, *Sox10*) in the interfrontal suture of E16.5 mice (total 6,632 cells; ref. 25). (**B**) The predicted incoming relative strength of the signaling pathways in the hypodermis (HD) cell population at E16.5, based on interfrontal suture scRNA-Seq data at E16.5. (**C**) scRNA-Seq data analysis of embryonic interfrontal mouse suture at E16.5: chord diagrams of inferred IGF, EGF, and FGF signaling networks from the various cell types to the HD population. Top subpanels show cell-cell interactions. Segments with large arrows represent signaling targets, and inner bars represent signaling sources in which the colors indicate signaling targets. The thickness of each string indicates the number of different interaction pairs colored by cell clusters. Bottom subpanels show inferred ligand-receptor interactions (total 6,632 cells; ref. 25). CEC, capillary endothelial cells; DC, dendritic cells; DM, dura mater; FS, frontal suture clusters; HD, hypodermis; MC, mast cells; MO, monocytes; MP, macrophages; OB, osteoblasts; OF, osteogenic front; PC, pericytes; SM, suture mesenchyme.

Notably, scRNA-Seq data analyses show that both *Kctd1* and *Kctd15* are expressed in mesenchymal cells and the osteogenic front of the embryonic interfrontal suture at E16.5 (cells that are derived from NCCs and have differentiated to not express NCC markers *Pax3* and *Sox10*) (Figure 9A) (25). Moreover, these interfrontal mesenchymal suture cells and cells of the osteogenic front normally express several growth factors that are known to promote keratinocyte migration or proliferation (including keratinocyte growth factor [*Kgf/Fgf7*], *Igf1*, *Igf2*, and *Fgf10*) (Figure 9A and Supplemental Figure 11A), suggesting that the structural midline skull/suture defects in *Wnt1Cre^+^Kctd1^fl/fl^Kctd15^fl/fl^* mice likely cause ACC as a consequence of reduced spatiotemporal expression of these keratinocyte-promoting growth factors at that site during development. Receptor-ligand interaction analyses of these scRNA-Seq data also highlight extensive interactions between interfrontal suture mesenchymal cells/osteogenic front cells and cells of the hypodermis, implicating NCC-derived suture mesenchymal cells as signaling hubs that instruct differentiation processes of the overlying skin (Figure 9, B and C; Supplemental Figure 11B; and Supplemental Figure 12). These include incoming signals into the hypodermis by IGF, EGF, and FGF families of growth factors as well as other growth factors or signaling molecules (Figure 9, B and C, Supplemental Figure 11B, and Supplemental Figure 12).

**Figure 10:**
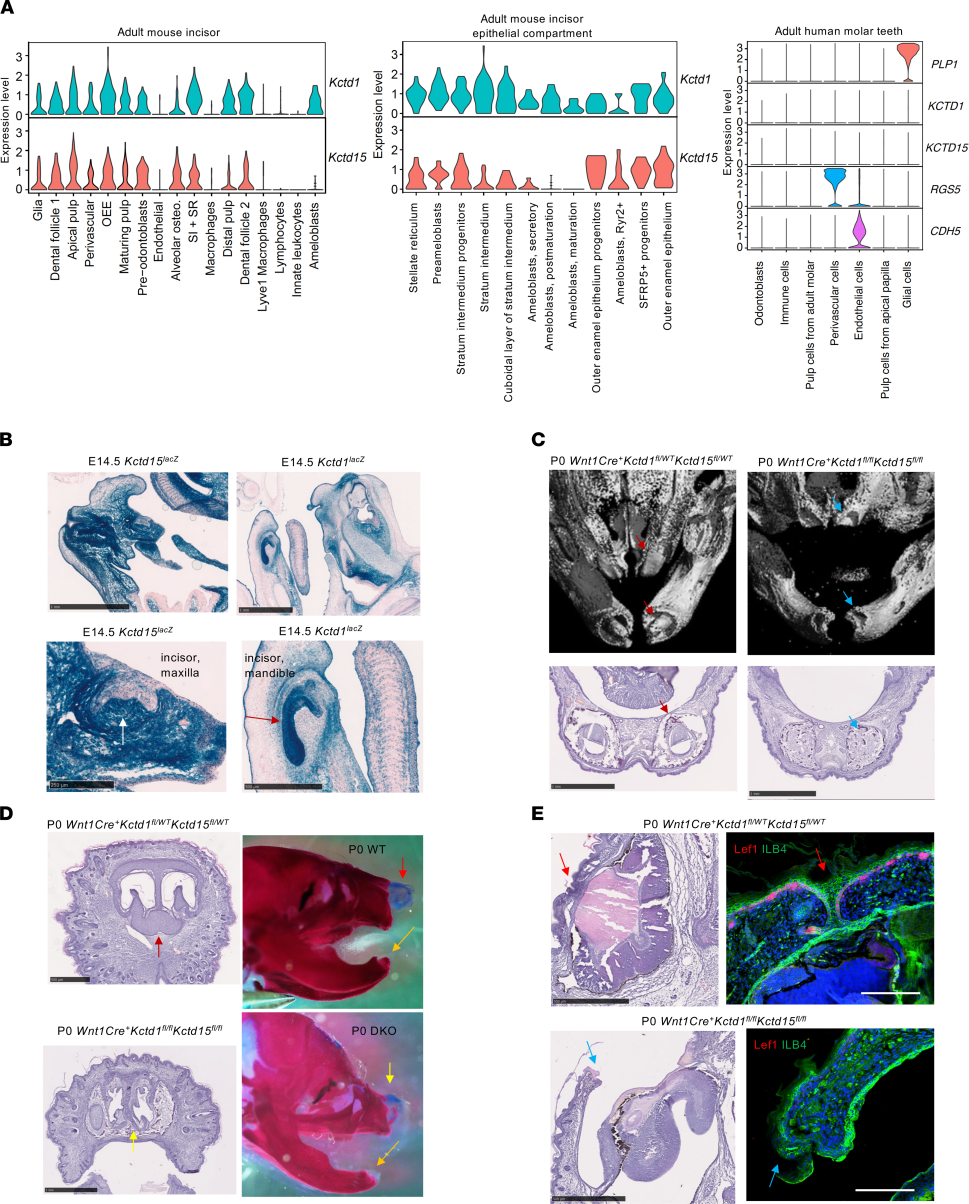
*Wnt1Cre^+^Kctd1^fl/fl^Kctd15^fl/fl^* mice show absence of incisors and defects in nose and eyelid development. (**A**) Expression of *Kctd1* and *Kctd15* in adult mouse incisors (left) and their epithelial compartment (middle), or in human molar teeth (right) (mouse incisor scRNA-Seq: 2,880 total cells; epithelial component: 267 total cells; human molar teeth: 41,674 total cells; ref. 50). OEE, outer enamel epithelium; SI, stratum intermedium; SR, stellate reticulum. (**B**) X-gal staining of E14.5 *Kctd15^LacZ/WT^* and *Kctd1^LacZ/WT^* embryos shows *Kctd1* and *Kctd15* expression in nasal cartilage/bone and teeth. *Kctd1* is expressed at E14.5 mainly in tooth epithelium (red arrow) and less in the dental papilla. *Kctd15* is expressed at E14.5 mainly in the tooth papilla (white arrow) and less in the tooth epithelium. Scale bars: top, 1 mm; bottom left, 250 μm; bottom right, 500 μm. (**C**) μCT images and histology show absence of incisors in P0 *Wnt1Cre^+^Kctd1^fl/fl^Kctd15^fl/fl^* mice (blue arrows) that are present in heterozygotes (red arrows). Scale bars: 1 mm. (**D**) Diminished nasal bones and nasal cartilage (yellow arrows) result in nasal airway abnormalities in P0 *Wnt1Cre^+^Kctd1^fl/fl^Kctd15^fl/fl^* mice (control mice: red arrows). Incisors (orange arrow) are absent in P0 *Wnt1Cre^+^Kctd1^fl/fl^Kctd15^fl/fl^* mice (DKO). Scale bars: top, 500 μm; bottom, 1 mm. (**E**) P0 *Wnt1Cre^+^Kctd1^fl/fl^Kctd15^fl/fl^* mice have open eyelids at birth (blue arrows), whereas WT littermates have fused eyelids at birth (red arrows). Lef1/isolectin B4–FITC (ILB4) labeling. Scale bars: H&Es, 500 μm; immunolabeling, 100 μm.

SEN syndrome patients often have tooth abnormalities, including an absence of incisors (7). Tooth formation occurs through interactions between the dental epithelium and the underlying mesenchyme that forms the dental papilla and is NCC-derived as well. scRNA-Seq data analyses show expression of both *Kctd1* and *Kctd15* in NCC-derived cells of the incisors during development (Figure 4D). In cell populations of adult mouse incisors, *Kctd1* and *Kctd15* show a partially overlapping expression pattern, including in the dental follicle, whereas no high expression of *KCTD1* or *KCTD15* is observed in adult human molar teeth (Figure 10A). We observed strong expression of *Kctd1* in particularly the dental epithelium of incisors at E14.5, whereas at that time *Kctd15* was strongly expressed in the dental papilla and to a lesser degree in the dental epithelium (Figure 10B). Lack of KCTD1 and KCTD15 in the dental epithelium (*K14Cre^+^Kctd1^fl/fl^Kctd15^fl/fl^* mice) did not affect incisor formation, whereas their absence in the NCC lineage (and thus the dental papilla) in *Wnt1Cre^+^Kctd1^fl/fl^Kctd15^fl/fl^* mice resulted in absence of mandibular and maxillary incisors (Figure 10, C and D).

Nasal bones are derived from cranial NCCs as well, and *Wnt1Cre^+^Kctd1^fl/fl^Kctd15^fl/fl^* newborn mice had diminished nasal bones and cartilage that led to abnormal nasal airways, resembling the facial dysmorphism with nose abnormalities that has been described in patients with *KCTD1* or *KCTD15* mutations (Figure 8, A, C, and D, and Figure 10D). In addition, we observed that *Wnt1Cre^+^Kctd1^fl/fl^Kctd15^fl/fl^* mice had open eyes with abnormal eyelids when assessed at E17.0 or P0 (Figure 8, A and D, and Figure 10E). This finding suggests an important role of NCC-derived periocular mesenchymal cells for proper eyelid morphogenesis during development.

Collectively, the distinct phenotypes in *Wnt1Cre^+^Kctd1^fl/fl^Kctd15^fl/fl^* mice and *K14Cre^+^Kctd1^fl/fl^Kctd15^fl/fl^* mice provide the cellular origin for the clinical abnormalities observed in patients with *KCTD1* or *KCTD15* mutations: hair, sebaceous gland, and sweat gland abnormalities are due to loss of KCTD1/KCTD15 in keratinocytes, whereas ACC, nasal abnormalities, and incisor defects are caused by loss of KCTD1/KCTD15 in NCCs.

### Non-redundant functions of KCTD1 and KCTD15.

**Figure 11:**
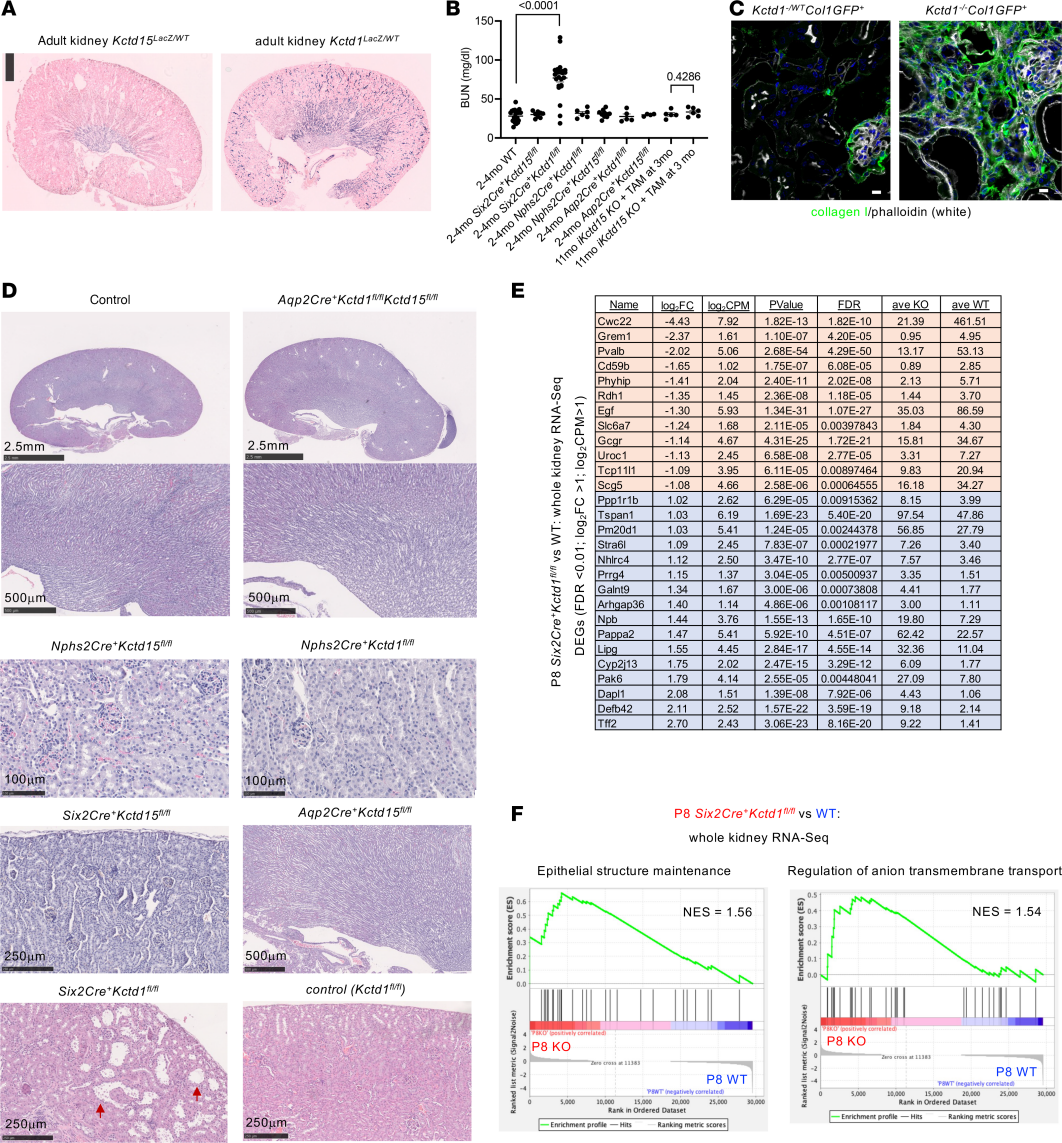
KCTD1 but not KCTD15 is required for renal function. (**A**) *Kctd15* is expressed in the kidney mainly in medullary collecting ducts, whereas *Kctd1* is expressed in all distal nephron segments. X-gal staining in adult kidneys of *Kctd1^Lacz/WT^* and *Kctd15^Lacz/WT^* mice. (**B**) *Six2Cre^+^Kctd1^fl/fl^* mice show elevated blood urea nitrogen (BUN), whereas inactivation of KCTD15 in the kidney during development or in the adult does not affect renal function. Inducible inactivation of KCTD15 in all cells (*β*-*actin-CreERT2^+^Kctd15^fl/fl^* mice [iKctd15 KO] treated with tamoxifen [TAM] at 3 months of age and assessed at 11 months of age) does not increase BUN. Mean ± SEM; *P* values (Mann-Whitney test). (**C**) Interstitial fibrosis in kidneys of *Kctd1^–/–^Col1GFP^+^* mice (green, type I collagen). Seven-month-old *Kctd1^–/–^Col1GFP^+^* and *Kctd1^–/WT^Col1GFP^+^* mice. Scale bars: 10 μm. (**D**) Normal kidney morphology in adult *Aqp2Cre^+^Kctd1^fl/fl^Kctd15^fl/fl^* mice, *Aqp2Cre^+^Kctd15^fl/fl^* mice, *Nphs2Cre^+^Kctd15^fl/fl^* mice, or *Six2Cre^+^Kctd15^fl/fl^* mice. Adult (3-month-old) *Six2Cre^+^Kctd1^fl/fl^* mice show abnormal and dilated distal nephron segments in the kidney cortex (red arrows). (**E**) DEGs in P8 kidneys of *Six2Cre^+^Kctd1^fl/fl^* mice and their nephron segment location (compared with P8 controls). DEGs of whole-kidney bulk RNA-Seq defined by: FDR < 0.01; log_2_(fold change) > 1; log_2_(CPM) > 1. CPM, counts per million. (**F**) Significantly upregulated pathways according to GSEA in P8 kidneys of *Six2Cre^+^Kctd1^fl/fl^* mice include “epithelial structure maintenance” and “anion transmembrane transport.” NES, normalized enrichment score.

While skin defects are found in patients with either *KCTD1* or *KCTD15* mutations, consistent with the overlapping expression pattern of *KCTD1* and *KCTD15* in the affected cell types, renal abnormalities occur in SEN syndrome patients with *KCTD1* mutations, whereas cardiac outflow tract (OFT) defects have been reported in patients with *KCTD15* mutations (7, 8). To assess the reason for this difference, we analyzed the expression of *Kctd1* and *Kctd15* in these organs. We found that *Kctd1* and *Kctd15* show a largely non-overlapping expression pattern in the kidney (Figure 11A and Supplemental Figure 9, A and B). Especially in the distal convoluted tubule (DCT), only *Kctd1* but not *Kctd15* was highly expressed (Supplemental Figure 9A) (26). Inactivation of *Kctd1* in the kidney (*Six2Cre^+^Kctd1^fl/fl^* mice) results in a terminal differentiation defect in the DCT that impairs renal function and leads to a severe interstitial renal fibrosis in the adult with aging, similarly as seen in SEN syndrome patients (Figure 11, B–D) (11, 27). Analysis of RNA-Seq data from P8 kidneys of *Six2Cre^+^Kctd1^fl/fl^* mice shows expression changes in genes that function in epithelial structure maintenance and anion transmembrane transport (Figure 11, E and F, and Supplemental Figure 9C). In contrast, inactivation of *Kctd15* in the kidney during development or in the adult did not affect kidney function (Figure 11, B and D). Thus, KCTD1 is critical for DCT function, and its loss cannot be compensated for by KCTD15 in the DCT, which is not expressed at similar levels in this kidney segment, explaining why patients with *KCTD1* mutations develop renal abnormalities.

**Figure 12:**
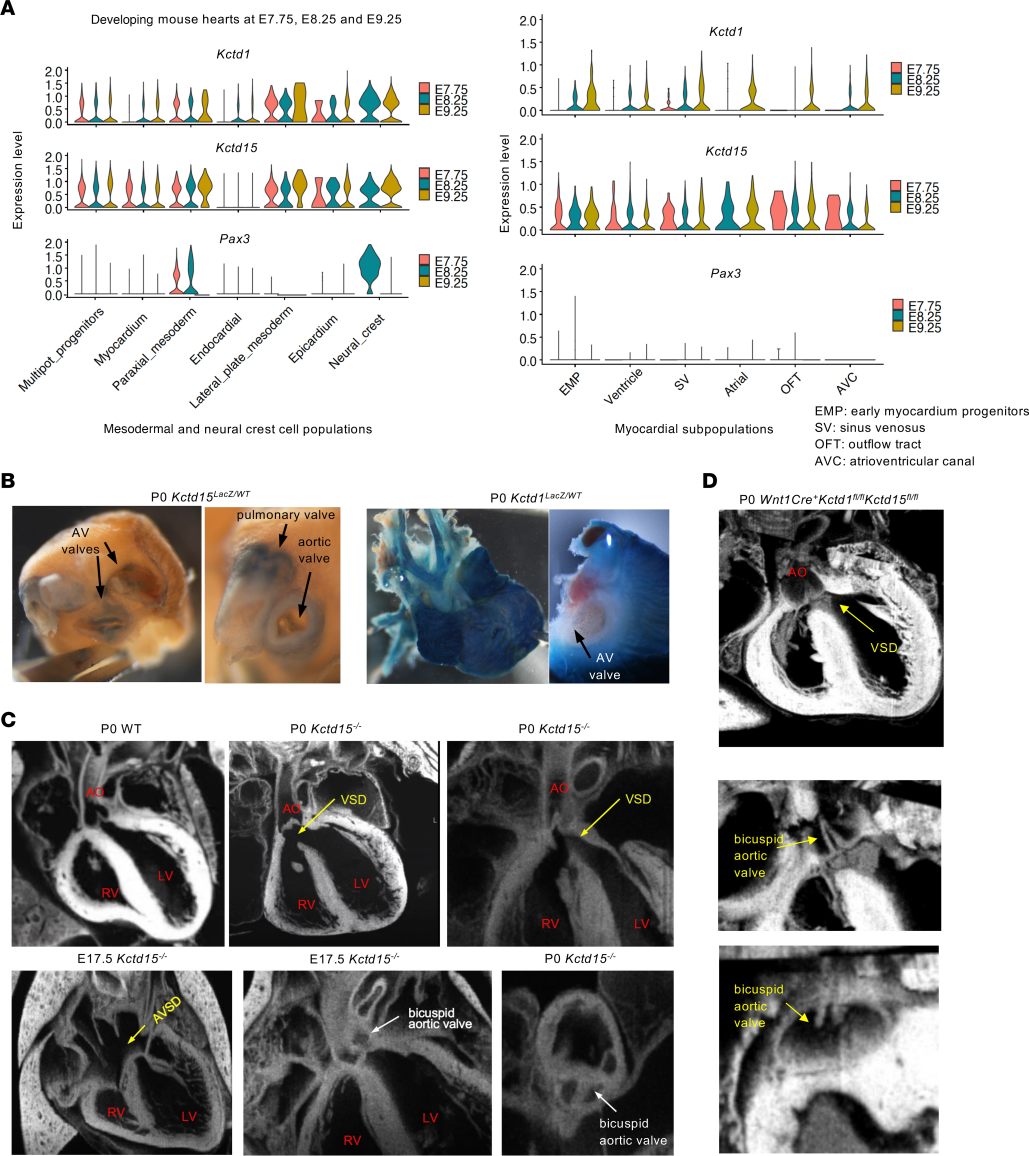
KCTD15 is required for the proper development of the perimembranous ventricular septum and the aortic valve. (**A**) Expression of *Kctd1*, *Kctd15*, and *Pax3* during early mouse heart development (E.7.75, E8.25, and E9.25). *Kctd15* is much more highly expressed than *Kctd1* in the outflow tract and atrioventricular canal at E7.75. Violin plots of scRNA-Seq data separated by mesodermal and NCC populations (left) and myocardial subpopulations (right) (total 21,988 cells; ref. 20). (**B**) Hearts of newborn *Kctd15^LacZ/WT^* mice (P0) show X-gal staining (blue) of cardiac valves and vessels. Hearts of newborn *Kctd1^LacZ/WT^* mice (P0) show X-gal staining (blue) of ventricular and atrial myocardium, but no strong labeling is observed in cardiac valves. AV, atrioventricular. (**C**) Episcopic fluorescence image capture (EFIC) imaging of hearts from *Kctd15^–/–^* mice and littermate controls at P0 or E17.5. In contrast to P0 WT hearts, hearts from P0 *Kctd15^–/–^* mice show a subaortic membranous ventricular septal defect (VSD), which can be accompanied by an overriding aorta, or an atrioventricular septal defect (AVSD), as well as a bicuspid aortic valve. AO, aorta; LV, left ventricle; RV, right ventricle. (**D**) EFIC imaging of hearts from P0 *Wnt1Cre^+^Kctd1^fl/fl^Kctd15^fl/fl^* mice shows a subaortic membranous VSD, an overriding aorta, and a bicuspid aortic valve.

Cardiac NCCs give rise to several structures of the cardiac OFT and atrioventricular canal (AVC) (28). scRNA-Seq data of NCCs between E8.5 and E10.5 show that *Kctd15* and *Kctd1* are both expressed in cardiac NCC populations (Figure 4F, Figure 12A, and Supplemental Figure 7B) (17, 19–21). In addition, scRNA-Seq data from developing mouse hearts show that in cells forming the cardiac OFT and the AVC, *Kctd15* is highly expressed at E7.75, whereas *Kctd1* is not expressed in these cells at that early time point but is expressed later on at E9.25. In contrast, expression of both *Kctd1* and *Kctd15* is observed in cardiac NCCs at E8.25 and E9.25 (Figure 12A). Moreover, *Kctd1* and *Kctd15* were both also expressed in multiple heart cell populations other than cardiac NCC–derived populations (Figure 12A). Analysis of scRNA-Seq data from human hearts during development and in the adult largely recapitulated these differences in the expression pattern between *Kctd1* and *Kctd15* in the mouse heart (Supplemental Figure 8) (29–31). X-gal staining of hearts from P0 *Kctd1^lacZ/WT^* and *Kctd15^lacZ/WT^* mice showed *Kctd15* to be mainly expressed in cells of the cardiac valves and vessels, whereas *Kctd1* was mainly expressed in myocardial cells (Figure 12B).

We found in *Kctd15^–/–^* mice congenital subaortic perimembranous ventricular septal defects and bicuspid aortic valves (analyzed at E17.5 or P0 by episcopic fluorescence image capture microscopy) (Figure 12C). These cardiac defects in structures whose proper development is dependent on cardiac NCCs were not observed in *Kctd1^–/–^* mice or WT littermate mice. These defects, together with the observation of cardiac defects in cardiac NCC–derived structures in patients with *KCTD15* mutations (8), identify KCTD15 as an important regulator of the formation of the subaortic ventricular septum and of the aortic valve and show that its deficiency cannot be compensated for by KCTD1 during their development.

The *Wnt1Cre* strain allows inactivation of genes in NCC populations that give rise to cardiac structures only after about E8.5 (23). Notably, the heart defects observed in *Kctd15^–/–^* mice were also observed in *Wnt1Cre^+^Kctd1^fl/fl^Kctd15^fl/fl^* mice but not in *Wnt1Cre^+^Kctd15^fl/fl^* mice (Figure 12D). The observation of these cardiac defects in *Kctd15^–/–^* mice and *Wnt1Cre^+^Kctd1^fl/fl^Kctd15^fl/fl^* mice but not in *Kctd1^–/–^* mice and *Wnt1Cre^+^Kctd15^fl/fl^* mice is consistent with the observation that *Kctd15* but not *Kctd1* is expressed at E7.75 in cardiac OFT structures (thus, loss of KCTD15 cannot be compensated for by KCTD1 in these cells), whereas both *Kctd1* and *Kctd15* are expressed in cardiac NCCs at later stages (and compensation likely can occur between these two in these cells). This indicates that for the proper development of the perimembranous ventricular septum and the aortic valve, KCTD15 function is likely critical already at an early time point (~E7.75) in cells of the developing cardiac OFT, whereas both KCTD1 and KCTD15 are important in cardiac NCCs afterward.

## Discussion

**Figure 13:**
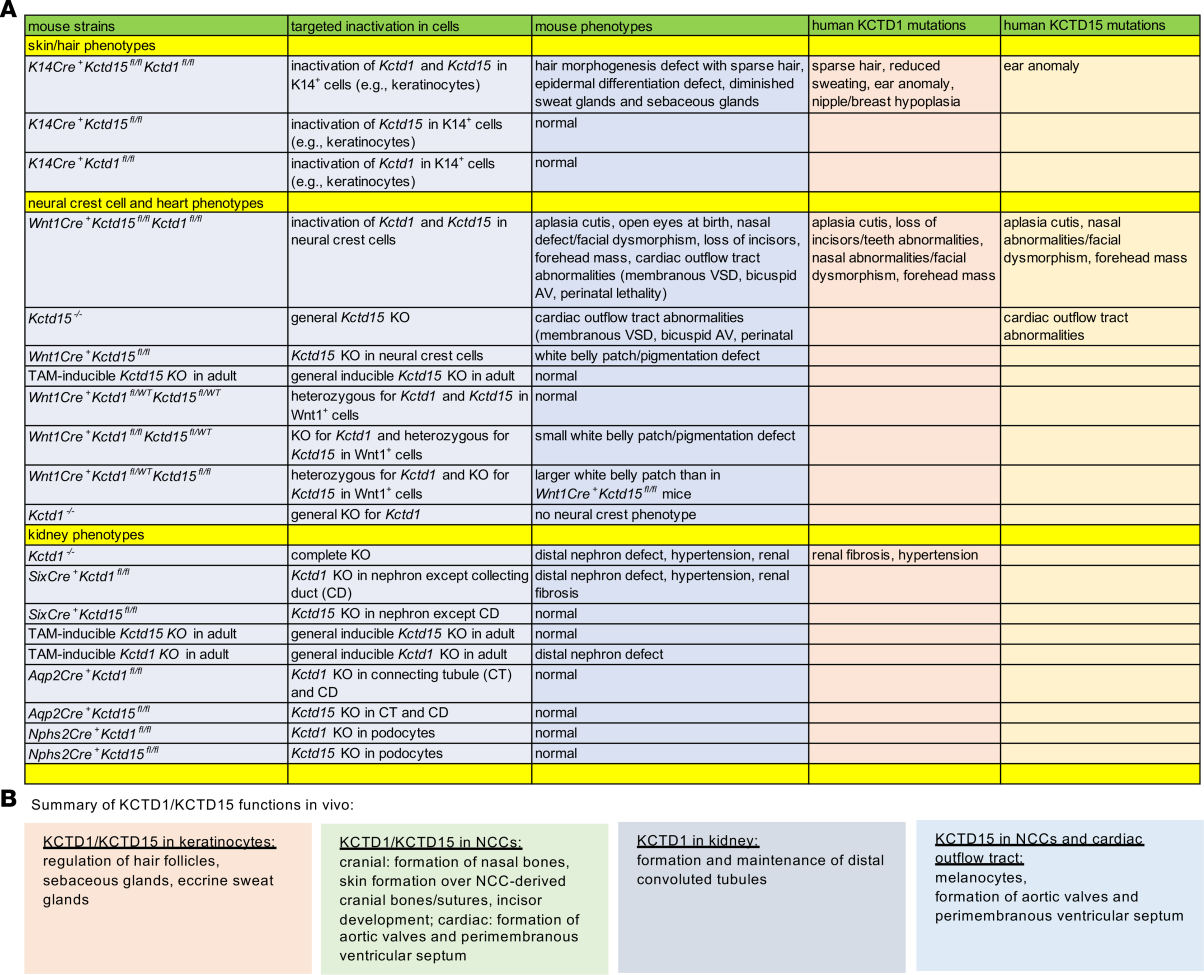
Overlap of functions of KCTD1/KCTD15 complexes in mice and humans. (**A**) Comparison of phenotypes in mouse strains generated in this study and patients with *KCTD1* or *KCTD15* mutations. (**B**) Summary of proposed KCTD1/KCTD15 functions.

We demonstrate that KCTD1 and KCTD15 regulate the development of skin appendages and specific NCC populations (Figure 13, A and B). Combined loss of both KCTD1 and KCTD15 in NCCs causes midline membranous ACC-like scalp epidermal defects overlying abnormalities in NCC-derived cranial bones/sutures, whereas their inactivation in keratinocytes does not result in this phenotype. This suggests that membranous ACC is a neurocristopathy and is not caused by a primary keratinocyte defect. Thus, the epidermal defect in ACC in patients with *KCTD1* or *KCTD15* mutations is likely a secondary consequence of abnormalities in cranial NCC-derived mesenchymal cell populations that lack KCTD1/KCTD15 function. The data demonstrate a previously unknown role of cranial NCC-derived mesenchymal cells of midline cranial sutures in orchestrating the formation of the overlying scalp epidermis.

The observations that ACC-like lesions and skin appendage defects occur only in mice that lack both KCTD1 and KCTD15 in NCCs or keratinocytes, respectively, but not when only one of these proteins is inactivated, that these developmental defects phenocopy the clinical findings in patients with heterozygous *KCTD1* or *KCTD15* mutations, and that these mutations result in amyloid-like aggregates that sequester both KCTD1 and KCTD15 WT proteins as well, are consistent with a dominant-negative effect of these *KCTD1* or *KCTD15* mutations that result in a combined inactivation of KCTD1 and KCTD15 in cells in which both are expressed. The midline skull abnormalities that are spatiotemporally linked to ACC-like lesions in *Wnt1Cre^+^Kctd1^fl/fl^Kctd15^fl/fl^* mice suggest that ACC is a consequence of diminished inductive signals from NCC-derived mesenchymal cells that would normally promote proper epidermis formation at that site. Consistent with this hypothesis, scRNA-Seq data show that NCC-derived mesenchymal cells of the embryonic interfrontal suture express several growth factors that are known to promote keratinocyte migration or proliferation (including *Kgf/Fgf7*, *Fgf10*, *Igf1*, and *Igf2*) (32, 33). Notably, *Igf1* was the most highly expressed among those growth factors in the interfrontal suture, and epidermal *Igf1r* deficiency results in epidermal thinning (33), similarly as observed in ACC-like lesions in *Wnt1Cre^+^Kctd1^fl/fl^Kctd15^fl/fl^* mice.

ACC-like lesions in *Wnt1Cre^+^Kctd1^fl/fl^Kctd15^fl/fl^* mice resemble particularly the membranous type of ACC in humans, characterized by a flattened epidermis of the midline/vertex scalp, which is also in a large proportion of cases linked to underlying defects of the bony structures and heterotopic meningeal/brain tissue (1). Thus, a defect in cranial NCCs is likely the cellular origin of membranous ACC in humans.

As KCTD1 and KCTD15 both act as inhibitors of AP-2 transcription factors (14, 15), loss of KCTD1/KCTD15 function may impair cellular differentiation owing to increased AP-2 activity, since there is a requirement for downregulating AP-2 activity to achieve proper keratinocyte maturation (34). However, heterozygosity for *Tfap2a* and *Tfap2b* did not rescue skin appendage defects in mice lacking KCTD1/KCTD15 in keratinocytes. Thus, KCTD1/KCTD15 inactivation may result in skin appendage defects through AP-2–independent downstream mechanisms. Alternatively, lack of KCTD1/KCTD15 may result in skin defects due to combined changes in both AP-2–dependent and AP-2–independent downstream pathways, which would explain why heterozygosity for *Tfap2a* and *Tfap2b* is not sufficient to rescue these skin defects.

*Kctd1* and *Kctd15* are both expressed in NCCs, albeit at different levels in specific subpopulations. For example, *Kctd15* is more highly expressed than *Kctd1* in NCC-derived melanocytes. This explains why the loss of KCTD15 cannot be compensated for by KCTD1 in melanocytes and why the loss of KCTD15 alone is sufficient to result in pigmentation defects. However, KCTD1 can prevent pigmentation defects in mice heterozygous for *Kctd15* in melanocytes, which is consistent with interchangeable and compensatory functions between KCTD1 and KCTD15. That these pigmentation defects are a consequence of NCC abnormalities is supported by the observation of a similar phenotype in mouse mutants of regulators of NCCs (*Pax3*- or *Tfap2a*-mutant mice) (23, 35, 36).

The perimembranous ventricular septal defect and bicuspid aortic valve, affecting cardiac NCC–derived structures, occurred in *Kctd15^–/–^* mice and *Wnt1Cre^+^Kctd1^fl/fl^Kctd15^fl/fl^* mice but not in *Kctd1^–/–^* mice and *Wnt1Cre^+^Kctd15^fl/fl^* mice. Similarly, the reported cardiac defects in patients with the *KCTD15^D104H^* mutation affect cardiac NCC–derived structures (8). This suggests that KCTD15 is a critical regulator of the development of the aortic valve and the perimembranous ventricular septum, while loss of KCTD1 can be compensated for by KCTD15 in their development. Notably, Wnt1Cre^+^ activity is absent in NCCs that contribute to heart structures at about E8.5 and occurs only afterward (23). The scRNA-Seq data in developing mouse hearts show that both *Kctd1* and *Kctd15* are expressed in cardiac NCCs at E8.25 and E9.25, albeit *Kctd15* is also expressed earlier (~E7.75) in other cell types that contribute to the formation of the cardiac OFT and AVC. This suggests that loss of KCTD15 in both cardiac NCCs and other cardiac cells contributes to the formation of the observed cardiac defects (explaining the phenotype in *Kctd15^–/–^* mice) while if KCTD15 is lacking only in cardiac NCCs after E8.5 (*Wnt1Cre^+^Kctd15^fl/fl^* mice) KCTD1 can compensate for its loss and the combined loss of both KCTD1 and KCTD15 in Wnt1^+^ cardiac NCCs is required to produce the same phenotype (explaining that the same cardiac phenotype as in *Kctd15^–/–^* mice occurs also in *Wnt1Cre^+^Kctd1^fl/fl^Kctd15^fl/fl^* mice but not in *Kctd1^–/–^* mice and *Wnt1Cre^+^Kctd15^fl/fl^* mice). Alternatively, loss of KCTD15 in cells that give rise to cardiac NCCs at a time point before Wnt1Cre activity occurs contributes to the cardiac phenotype. The latter scenario would be similar to the observation of cardiac OFT abnormalities in *Pax3* or *Tfap2a* germline mouse mutants (both transcription factors are critical for NCC function) (23, 35, 36), while conditional inactivation of *Pax3* or *Tfap2a* with the *Wnt1Cre* strain does not result in cardiac defects but instead results in a white belly patch (23, 37). This has led to the hypothesis that Pax3 and AP-2α are important in regulating cells that become cardiac NCCs at a very early time point before *Wnt1Cre* activity occurs, whereas they retain importance in NCC-derived melanoblasts at a time point after *Wnt1Cre* is active. Similarly, *Wnt1Cre^+^Kctd15^fl/fl^* mice had a white belly patch due to the absence of melanocytes, but no cardiac defects. The observation of bicuspid aortic valves and perimembranous ventricular septal defects in *Wnt1Cre^+^Kctd1^fl/fl^Kctd15^fl/fl^* mice demonstrates that these structures require KCTD1/KCTD15 in cardiac NCCs for their development.

The findings in our mouse models provide a comprehensive characterization of the cellular origin of the various pathologies observed in patients with mutations in *KCTD1* or *KCTD15*. Notably, the spectrum of clinical abnormalities due to *KCTD1* mutations is better characterized than that due to *KCTD15* mutations, as more SEN syndrome families with *KCTD1* mutations have been reported than families with *KCTD15* mutations. It is likely that more overlap in ectodermal and NCC phenotypes between patients with *KCTD1* and *KCTD15* mutations will be observed once additional patients with *KCTD15* mutations are reported.

Our data suggest that the paralogs KCTD1 and KCTD15 can form multimeric complexes in vivo depending on their relative abundance in a specific cell type. In keratinocytes and cranial NCCs, in which both KCTD1 and KCTD15 are present, KCTD15 can compensate for the loss of KCTD1 and vice versa, whereas KCTD1 cannot be compensated for by KCTD15 in the DCT of the kidney and KCTD15 cannot be compensated for by KCTD1 in melanoblasts and in a subset of cardiac cell populations that give rise to the perimembranous ventricular septum and the aortic valve. The striking overlap between human and mouse phenotypes suggests conserved functions of KCTD1 and KCTD15 between humans and mice and that the pathomechanisms in our mouse models apply to these human diseases as well. Collectively, our data provide the pathomechanistic basis for the organ-specific abnormalities seen in patients with *KCTD1* or *KCTD15* mutations, identify KCTD1/KCTD15 complexes as critical regulators of ectodermal and NCC functions, and define membranous ACC as a neurocristopathy.

## Methods

### Mice.

We reported the generation of *Kctd1^lacZ/WT^* mice, *Kctd1^–/–^* mice, and floxed *Kctd1* (*Kctd1^fl/fl^*) mice (11). *Kctd15tm1a^(EUCOMM)Wtsi^* mice were obtained from the Mutant Mouse Resource and Research Centers (MMRRC) (048308-UCD, C57BL/6N-Kctd15^tm1a(EUCOMM)Wtsi^/MbpMmucd). The tm1a allele is a KO first allele (lacZ reporter-tagged insertion with conditional potential) (38). *LoxP* sites flank exon 4, and removal of exon 4 results in disruption of the critical BTB domain after crossing of these mice with β-actin-Cre^+^ mice (tm1b allele, representing *Kctd15^–/–^* mice). Subsequently, the Cre allele was crossed out. The *Kctd15^tm1a^* allele after β-actin-Cre^+^ excision of the floxed region maintains a *lacZ* cassette, allowing monitoring of β-galactosidase expression from the endogenous *Kctd15* locus: heterozygous *Kctd15^WT/lacZ^* mice serve as *Kctd15* reporter mice. *Kctd15^tm1a^* mice were crossed with a FLP-deleter mouse strain to remove the *lacZ* and *neoR* cassettes that are flanked by FRT sites, thereby allowing for normal *Kctd15* expression, but maintaining *loxP* sites flanking exon 4 of *Kctd15* (tm1c) (*Kctd15^fl/fl^* mice). These mice were crossed with Cre lines to establish cell type–specific conditional-*Kctd15*-KO mice: Krt14Cre^+^ mice (International Mouse Strain Resource [IMSR] catalog JAX:018964, RRID:IMSR_JAX:018964), Six2Cre^+^ mice (IMSR catalog JAX:009606, RRID:IMSR_JAX:009606; ref. 39), Aqp2Cre^+^ mice (IMSR catalog JAX:006881, RRID:IMSR_JAX:006881; using only female Aqp2Cre^+^ mice for matings), Nphs2Cre^+^ mice (IMSR catalog JAX:008205, RRID:IMSR_JAX:008205), Wnt1Cre2^+^ mice (IMSR catalog JAX:022501, RRID:IMSR_JAX:022501; using only female Wnt1Cre2^+^ mice for matings; ref. 40). Induced inactivation of *Kctd15* in the adult was achieved in *β*-*actin-CreERT2^+^Kctd15^fl/fl^* mice (crossing CAGGCreERT2^+^ mice [IMSR catalog JAX:004453, RRID:IMSR_JAX:004453; ref. 41] with *Kctd15^fl/fl^* mice) with injections of tamoxifen (T5648, Sigma-Aldrich; 6 mg/40 g body weight daily for 5 consecutive days i.p.) (41). Efficient Cre-mediated recombination and removal of exon 4 of *Kctd15* and of exon 3 of *Kctd1* were confirmed by RNA-Seq in epidermal sheets from *K14Cre^+^Kctd15^fl/fl^* mice and *K14Cre^+^Kctd1^fl/fl^* mice, respectively (Supplemental Figure 10C), as well as in *K14Cre^+^Kctd1^fl/fl^Kctd15^fl/fl^* mice. *Tfap2a^fl/fl^* mice (37) and *Tfap2b^fl/fl^* mice (42) have been reported. These mice were crossed with K14Cre^+^ mice to generate *K14Cre^+^Tfap2a^fl/fl^Tfap2b^fl/fl^* mice. Blood urea nitrogen was measured from serum with a Dri-Chem7000 chemistry analyzer (Heska). International guidelines for the care and use of laboratory research animals were followed. ARRIVE guidelines for reporting animal studies were followed.

### Immunoprecipitation and mass spectrometry.

FLAG-tagged KCTD1 (human C-terminal Myc-DDK–tagged KCTD1, transcript variant 1, pCMV6-Entry vector, RC226740, Origene), FLAG-tagged KCTD15 (human C-terminal Myc-DDK–tagged KCTD15, transcript variant 1, pCMV6-Entry vector, RC200838, Origene), or the pCMV6-Entry vector (C-terminal Myc-DDK tag, PS100001, Origene) was overexpressed in HEK293 cells. IP was performed 48 hours after transfection using a monoclonal anti-DDK (FLAG) antibody (TA50011-100, clone OTI4C5, Origene) and Dynabeads protein G (10004D, Life Technologies), followed by mass spectrometry. For co-IP experiments, an anti-KCTD1 antibody (antigen: synthetic peptide corresponding to amino acids 121–135 of human KCTD1; SAB1103965, Sigma-Aldrich) and an anti-KCTD15 antibody (ab106373, Abcam) were used. For IP of endogenous KCTD1 in HEK293 cells, 2 different anti-KCTD1 antibodies were used (SAB1103965, Sigma-Aldrich; and the anti-KCTD1 antibody from Biorbyt, orb184655), and immunoblotting was performed with the anti-KCTD15 antibody.

### Immunolabeling and morphological analyses.

Tissues were fixed in 4% paraformaldehyde, processed, and embedded in paraffin. Skin thickness was defined as the distance between the granular layer of the epidermis and the panniculus carnosus muscle. Immunolabeling experiments were performed on slides that were citrate buffer–treated for heat-mediated antigen retrieval. Sections were permeabilized in 0.5% Triton X-100 and blocked with serum in which the secondary antibodies were raised. Primary antibodies are listed in Supplemental Table 1. DAPI was used to stain nuclei (D3571, Thermo Fisher Scientific). Secondary Alexa Fluor 488/555/647 antibodies were used at a dilution of 1:200 (Thermo Fisher Scientific). FITC-conjugated Griffonia Simplicifolia Lectin I isolectin B4 (FL-1201, Vector Laboratories) was used at a dilution of 1:100. Hind paws were treated with dispase II (20 mg/mL) (D4693, Sigma-Aldrich) overnight and stained with Oil Red O to assess sebaceous glands and with Nile Blue to stain sweat ducts.

### Morphological analyses of mouse hearts and skulls.

P0 or embryonic mouse hearts were fixed in 4% paraformaldehyde and analyzed by episcopic fluorescence image capture microscopy (43). No cardiac abnormalities were detected in P0 *Kctd1^–/–^* mice, *Kctd15^–/+^* mice, *Wnt1Cre^+^Kctd15^fl/fl^* mice, or WT littermates, whereas congenital heart defects were observed in E16.5 to P0 *Kctd15^–/–^* mice or P0 *Wnt1Cre^+^Kctd1^fl/fl^Kctd15^fl/fl^* mice (*n* = 3–8 hearts per group examined). For μCT studies, P0 mouse skulls were fixed in 4% paraformaldehyde and imaged with a high-resolution μCT imaging system (μCT40, Scanco Medical AG). Scans were acquired using an 8 μm^3^ isotropic voxel size, 45 kVP, 177 μA, 300 milliseconds integration time, and were subjected to Gaussian filtration and segmentation. A threshold of 150 mg HA/cm^3^ was used to segment bone from soft tissue for 3D imaging. Skeletal preps with Alcian blue and Alizarin red were performed according to standard protocols.

### Semiquantitative reverse transcriptase PCR.

Melan-A mouse melanocytes were cultured in RPMI 1640 medium containing 10% FBS and 200 nM phorbol-12-myristate-13-acetate (TPA) (524400, Sigma-Aldrich). For siRNA-mediated knockdown of *Kctd15* or *Pax3*, cells were treated with Lipofectamine RNAiMax transfection reagent (13778030, Thermo Fisher Scientific) and SMARTPool siRNAs targeting mouse *Kctd15* or *Pax3* (ON-TARGETplus Mouse *Kctd15* siRNA L-052498-01-0005, ON-TARGETplus Mouse *Pax3* siRNA L-062276-01-0005) or control siRNA (Horizon, PerkinElmer) for 47 hours. RNA was isolated with Trizol (15596018, Thermo Fisher Scientific). cDNA was produced using the high-capacity cDNA reverse transcription kit with RNase inhibitor (4374966, Life Technologies). Semiquantitative reverse transcriptase PCR was performed using PowerUp SYBR Green Master Mix (A25742, Thermo Fisher Scientific) on a QuantStudio5 machine (Applied Biosystems). Two different primer pairs were used for the detection of *Kctd15* or *Pax3*. Results were normalized to levels for *36b4* (housekeeping gene control). Transcript levels were determined by the –ΔΔCt method. Experiments for all samples were performed in triplicate with *n* = 4 per experimental group. Primers used are listed in Supplemental Table 2.

### AP-2α binding assay.

ELISAs were performed using 200 nM AP-2α (TP304603, Origene) and increasing concentrations of KCTD1^WT^ (from 18 nM to 500 nM). An anti-KCTD1 antibody (LS‑C260993, LSBio) was used for detection. KCTD1^WT^ was premixed with KCTD1^H74P^ and KCTD1^G62D^ mutants at a ratio of 5:1 (500 nM of KCTD1 and 100 nM of KCTD1 SEN-associated mutants) overnight at 4°C.

### Thioflavin T fluorescence experiments and in vitro experiments with KCTD1 mutants.

Thioflavin T (ThT) (50 μM) was added to KCTD1^WT^ (500 nM) and to KCTD1^WT^ (500 nM) premixed overnight with 100 nM of KCTD1^H74P^ or KCTD1^G62D^ mutants. ThT fluorescence was measured with a Varian Cary Eclipse spectrofluorometer (Agilent Technologies). Measurements were collected at 25°C with excitation and emission wavelengths of 440 and 450–600 nm, respectively. Experiments were repeated 3 times with similar results.

### Cell culture experiments.

The HaCaT human keratinocyte cell line, HEK293 cells, and HeLa cells were obtained from the IRCCS SYNLAB SDN Biobank. Cells were grown in DMEM with 10% FBS and cultured at 37°C in a humidified atmosphere containing 5% CO_2_. For transfections of KCTD1^wt^, KCTD1^wt^/KCTD1^H74P^, or KCTD1^wt^/KCTD1^G62D^, 250 ng total DNA was used with Lipofectamine 3000 (L3000001, Thermo Fisher Scientific). For experiments with KCTD15 proteins, 75 ng total DNA was used. KCTD1^WT^ (eGFP tag), KCTD1^H74P^ or KCTD1^G62D^ mutants (eYFP tag), KCTD15^D104H^ mutants (eYFP tag), and KCTD15^WT^ (tRFP tag) constructs were purchased at CliniSciences (CUST-DNA 03022023-1 A/B/C). Confocal microscopy was conducted using a ×63 water immersion objective on the MICA microhub (Leica) platform. For live cell imaging, HaCaT cells were transfected with 250 ng of eGFP-KCTD1^WT^ vector. One image every 2 hours was recorded for a total time of 48 hours.

Cells were treated with Amytracker-630 (A630-MQ, Ebba Biotech AB). Imaging of cells before and after addition of Amytracker-630 showed that the signal was not due to autofluorescence of protein aggregates. Cell viability was assessed using the ATPlite assay (6016943, PerkinElmer). HaCaT cells were seeded in 96-well plates at a density of 8 × 10^3^ cells per well, and the ATPlite luminescence signal was detected using an automatic plate reader (Victor Nivo, PerkinElmer). Cell viability was assessed as a percentage with respect to the untreated cells.

### Prediction of the 3D structure of KCTD1/KCTD15 oligomers.

The pentameric structure of the KCTD15 homo-oligomers and of the KCTD1/KCTD15 hetero-oligomers was predicted using the AlphaFold v2.0 algorithm as implemented on the Colab server (https://colab.research.google.com/github/sokrypton/ColabFold/blob/main/AlphaFold2.ipynb) by considering combinations of subunits for KCTD15 and KCTD1. Predictions were performed without considering any homologous experimental template and with 3 as the number of recycles. The reliability of the AlphaFold predictions was assessed by analysis of the predicted aligned error (PAE) matrix, which provides a distance error for every pair of residues within the complex. The tendency of these proteins to form high-order assemblies formed by interacting pentamers was evaluated by consideration of 10 (5 KCTD1 + 5 KCTD15) chains. Because of the large number of residues involved, these analyses were performed by splitting of the proteins into the BTB and the C-terminal domains, and their tendency to form high-order assemblies was evaluated separately for each of them.

### RNA-Seq.

Total RNA samples were quantified using a Qubit 2.0 Fluorometer (Life Technologies), and RNA integrity was checked with a TapeStation 4200 (Agilent Technologies). For RNA-Seq library construction, rRNA depletion sequencing library was prepared using a QIAGEN FastSelect rRNA HMR Kit. For RNA-Seq library preparation, we used the NEBNext Ultra II RNA Library Preparation Kit for Illumina (New England Biolabs). Sequencing libraries were validated using the TapeStation 4200, quantified using a Qubit 2.0 Fluorometer (Thermo Fisher Scientific) as well as quantitative PCR (KAPA Biosystems), and loaded to an Illumina NovaSeq 6000 system for 150 bp paired-end sequencing. Raw sequence reads were trimmed to remove possible adapter sequences and low-quality reads using Trimmomatic v0.36 (44). The trimmed reads were mapped to the mouse GRCm38 reference genome using the STAR aligner v2.5.2b (45). Reads mapping to annotated genes were quantified using featureCounts from the Subread package v1.5.2 (46). Differential expression analysis was performed with DESeq2 (47). Genes with adjusted *P* values less of than 0.05 and absolute log_2_ fold changes greater than 1 were called as differentially expressed genes. GSEA was performed using GSEA v4.3.2 software (https://www.gsea-msigdb.org/gsea/index.jsp). Gene Ontology (GO) enrichment analyses were performed using ShinyGO 0.77 (http://bioinformatics.sdstate.edu/go/). Interaction analysis was conducted by STRING (https://string-db.org). RNA-Seq of P8 kidneys was previously described (11).

### Analysis of scRNA-Seq data.

For scRNA-Seq data sets where the expression matrix file and per-cell annotation were available, violin plots of genes of interest were generated by Seurat v4.3.0. For each scRNA-Seq data set where per-cell annotation was not available, we reanalyzed scRNA-Seq data using Cellranger v7.1.0 and Seurat v4.3.0 according to the bioinformatics analysis pipeline described in the original publication. For clustering, we generated multiple clusterings of different resolutions and manually picked the one optimally matching published clustering. Markers for all clusters were generated by FindAllMarkers function with default parameters. Known marker genes described in the original publication were used to annotate cell types. Cell-cell interaction networks were predicted using CellChat with default parameters.

### Statistics.

Statistical tests used are described in the figure legends. They include 2-tailed unpaired *t* test, 1-way ANOVA test with a Dunnett’s multiple-comparison test, 1-way ANOVA with test for linear trend, Kruskal-Wallis test with Dunn’s multiple-comparison test, and a Mann-Whitney test. *P* values of less than 0.05 were considered to be statistically significant.

### Study approval.

All animal studies were reviewed and approved by the Massachusetts General Hospital Institutional Animal Care and Use Committee.

### Data availability.

Raw data for graphs are provided in the Supporting Data Values file. RNA-Seq data were deposited to the NCBI Gene Expression Omnibus database (GEO GSE126326, GSE130864, and GSE233496). Expression values are listed in Supplemental Tables 3 and 4. scRNA-Seq data sets of mouse NCCs are available in GEO (GSE201257 and GSE129114). scRNA-Seq data sets of cardiac NCCs in mouse hearts from E10.5 to P7 and the developing cardiac OFT in mice at E12–E13 are available at the NCBI Sequence Read Archive (PRJNA562135 and PRJNA489304). scRNA-Seq data set of primate gastrulation and early organogenesis is available in GEO (GSE193007). Single-nucleus RNA-Seq data sets of adult human ventricles and developing human fetal heart at day 83 are available at the Broad Single Cell Portal (SCP1852 and SCP1020). scRNA-Seq data set of fetal human hearts at 4.5–5, 6.5, and 9 weeks after conception is available at https://www.spatialresearch.org/ scRNA-Seq data set of calvarial suturogenesis was deposited with FaceBase (FB00001013). scRNA-Seq data set on mouse incisors and human molar teeth is available in GEO (GSE146123). scRNA-Seq data of developing mouse skin are available at ArrayExpress (E-MTAB-11920).

## Author contributions

Experiments and/or data analyses were performed by JRR, HZ, GS, WZ, KED, BJG, GP, MS, WAD, CWL, LV, and AGM. AGM conceived the study, designed the experiments, and wrote the manuscript.

## Supplementary Material

Supplemental data

Supplemental tables 1-2

Supplemental table 3

Supplemental table 4

Supplemental video 1

Supplemental video 2

Supporting data values

## Version 1 12/19/2023

In-Press Preview

## Version 2 02/15/2024

Electronic publication

## References

[1] Bessis D (2017). The scalp hair collar and tuft signs: a retrospective multicenter study of 78 patients with a systematic review of the literature. J Am Acad Dermatol.

[2] Frieden IJ (1986). Aplasia cutis congenita: a clinical review and proposal for classification. J Am Acad Dermatol.

[3] Campbell W (1826). Case of congenital ulcer on the cranium of a fetus. Edin J Med Sci.

[4] Marneros AG (2015). Genetics of aplasia cutis reveal novel regulators of skin morphogenesis. J Invest Dermatol.

[5] Stephan MJ (1982). Origin of scalp vertex aplasia cutis. J Pediatr.

[6] Sutton RL (1935). Congenital defect of the skin of the newborn. Arch Dermatol Syph.

[7] Marneros AG (2013). Mutations in KCTD1 cause scalp-ear-nipple syndrome. Am J Hum Genet.

[8] https://www.ncbi.nlm.nih.gov/pmc/articles/PMC6777611/.

[9] Ji AX (2016). Structural insights into KCTD protein assembly and Cullin3 recognition. J Mol Biol.

[10] Wang D (2023). KCTD1 and scalp-ear-nipple (‘Finlay-Marks’) syndrome may be associated with myopia and thin basement membrane nephropathy through an effect on the collagen IV α3 and α4 chains. Ophthalmic Genet.

[11] Marneros AG (2020). AP-2β/KCTD1 control distal nephron differentiation and protect against renal fibrosis. Dev Cell.

[12] Hu L (2020). KCTD1 mutants in scalp‑ear‑nipple syndrome and AP‑2α P59A in Char syndrome reciprocally abrogate their interactions, but can regulate Wnt/β‑catenin signaling. Mol Med Rep.

[13] Smaldone G (2019). Molecular basis of the scalp-ear-nipple syndrome unraveled by the characterization of disease-causing KCTD1 mutants. Sci Rep.

[14] Ding X (2009). The interaction of KCTD1 with transcription factor AP-2alpha inhibits its transactivation. J Cell Biochem.

[15] Zarelli VE, Dawid IB (2013). Inhibition of neural crest formation by Kctd15 involves regulation of transcription factor AP-2. Proc Natl Acad Sci U S A.

[16] Chen G (2017). Specific and spatial labeling of P0-Cre versus Wnt1-Cre in cranial neural crest in early mouse embryos. Genesis.

[17] Soldatov R (2019). Spatiotemporal structure of cell fate decisions in murine neural crest. Science.

[18] Kastriti ME (2022). Schwann cell precursors represent a neural crest-like state with biased multipotency. EMBO J.

[19] Chen W (2021). Single-cell transcriptomic landscape of cardiac neural crest cell derivatives during development. EMBO Rep.

[20] de Soysa TY (2019). Single-cell analysis of cardiogenesis reveals basis for organ-level developmental defects. Nature.

[21] Liu X (2019). Single-cell RNA-seq of the developing cardiac outflow tract reveals convergent development of the vascular smooth muscle cells. Cell Rep.

[22] Zhai J (2022). Primate gastrulation and early organogenesis at single-cell resolution. Nature.

[23] Olaopa M (2011). Pax3 is essential for normal cardiac neural crest morphogenesis but is not required during migration nor outflow tract septation. Dev Biol.

[24] Mishina Y, Snider TN (2014). Neural crest cell signaling pathways critical to cranial bone development and pathology. Exp Cell Res.

[25] Holmes G (2020). Integrated transcriptome and network analysis reveals spatiotemporal dynamics of calvarial suturogenesis. Cell Rep.

[26] Ransick A (2019). Single-cell profiling reveals sex, lineage, and regional diversity in the mouse kidney. Dev Cell.

[27] Marneros AG (2021). Magnesium and calcium homeostasis depend on KCTD1 function in the distal nephron. Cell Rep.

[28] George RM (2020). The heart of the neural crest: cardiac neural crest cells in development and regeneration. Development.

[29] Hill MC (2022). Integrated multi-omic characterization of congenital heart disease. Nature.

[30] Miao Y (2020). Intrinsic endocardial defects contribute to hypoplastic left heart syndrome. Cell Stem Cell.

[31] Asp M (2019). A spatiotemporal organ-wide gene expression and cell atlas of the developing human heart. Cell.

[32] Seeger MA, Paller AS (2015). The roles of growth factors in keratinocyte migration. Adv Wound Care (New Rochelle).

[33] Gunschmann C (2013). Insulin/IGF-1 controls epidermal morphogenesis via regulation of FoxO-mediated p63 inhibition. Dev Cell.

[34] Li L (2019). TFAP2C- and p63-dependent networks sequentially rearrange chromatin landscapes to drive human epidermal lineage commitment. Cell Stem Cell.

[35] Johnson AL (2020). Early embryonic expression of AP-2α is critical for cardiovascular development. J Cardiovasc Dev Dis.

[36] Brewer S (2002). Requirement for AP-2alpha in cardiac outflow tract morphogenesis. Mech Dev.

[37] Brewer S (2004). Wnt1-Cre-mediated deletion of AP-2alpha causes multiple neural crest-related defects. Dev Biol.

[38] Skarnes WC (2011). A conditional knockout resource for the genome-wide study of mouse gene function. Nature.

[39] Kobayashi A (2008). Six2 defines and regulates a multipotent self-renewing nephron progenitor population throughout mammalian kidney development. Cell Stem Cell.

[40] Lewis AE (2013). The widely used Wnt1-Cre transgene causes developmental phenotypes by ectopic activation of Wnt signaling. Dev Biol.

[41] Hayashi S, McMahon AP (2002). Efficient recombination in diverse tissues by a tamoxifen-inducible form of Cre: a tool for temporally regulated gene activation/inactivation in the mouse. Dev Biol.

[42] Van Otterloo E (2018). AP-2α and AP-2β cooperatively orchestrate homeobox gene expression during branchial arch patterning. Development.

[43] Rosenthal J (2004). Rapid high resolution three dimensional reconstruction of embryos with episcopic fluorescence image capture. Birth Defects Res C Embryo Today.

[44] (2014). Trimmomatic: a flexible trimmer for Illumina sequence data. Bioinformatics.

[45] Dobin A (2013). STAR: ultrafast universal RNA-seq aligner. Bioinformatics.

[46] (2013). The Subread aligner: fast, accurate and scalable read mapping by seed-and-vote. Nucleic Acids Res.

[47] (2014). Moderated estimation of fold change and dispersion for RNA-seq data with DESeq2. Genome Biol.

[48] Jacob T (2023). Molecular and spatial landmarks of early mouse skin development. Dev Cell.

[49] Marneros AG (2013). BMS1 is mutated in aplasia cutis congenita. PLoS Genet.

[50] Krivanek J (2020). Dental cell type atlas reveals stem and differentiated cell types in mouse and human teeth. Nat Commun.

