# Supplementary material for: KCTD1/KCTD15 complexes control ectodermal and neural crest cell functions, and their impairment causes aplasia cutis
Source: J Clin Invest. 2023 Dec 19;134(4):e174138. doi: 10.1172/JCI174138 (PMC10866662; doi:10.1172/JCI174138)
Supplement: Supplemental data [file jci-134-174138-s079.pdf]

## SUPPLEMENTAL FIGURE LEGENDS

**Supplemental Figure 1: KCTD1 and KCTD15 can form stable pentamers in any stochiometry.**

**A,** AlphaFold modeling of pentameric KCTD15 homomer (right) based on KCTD1 structure (left). Two different orientations of the three-dimensional model of the KCTD15 pentamer are shown. The BTB and the C-terminal domains of KCTD15 have been colored orange and green respectively.

**B**, Left: The reliability of the AlphaFold predictions was assessed by analyzing the Predicted Aligned Error (PAE) matrix, which provides a distance error for every pair of residues within the complex. The prevalence of the blue color, which is associated with low estimated errors of the distance of pairs of residues, is a strong indication of the global reliability of the model. These data support that KCTD1 and KCTD15 can form stable pentamers in any stochiometry. Top PAE matrix also shown in Figure 1E. Right: AlphaFold modeling of corresponding pentameric KCTD15/KCTD1 heteromers.

**C**, HaCaT cells transfected with $KCTD1^{wt}$ and $KCTD1^{H74P}$-eYFP show aggregates with intrinsic fluorescence (excitation wavelength: 350nm; emission wavelength: 450nm). Scale bars, 10$\mu$m.

**D**, $KCTD1^{wt}$/$KCTD1^{H74P}$ protein aggregates and $KCTD1^{wt}$/$KCTD1^{G62D}$ protein aggregates in human keratinocytes (HaCaT cells) from experiment shown in Figure 2E before addition of Amytracker-630, demonstrating that the Amytracker-630 signal is not due to autofluorescence. Scale bars, 10$\mu$m.

**Supplemental Figure 2: ACC-associated KCTD1 mutants sequester normal KCTD15 protein in amyloid-like aggregates and the ACC-associated KCTD15 mutant sequesters normal KCTD1 protein in amyloid-like aggregates in HaCaT cells.**

KCTD1$^{WT}$ (green) and KCTD15$^{WT}$ (red) proteins show an overlapping cellular localization pattern in the human keratinocyte cell line HaCaT and do not form amyloid-like aggregates. In contrast, KCTD1$^{H74P}$ and KCTD1$^{G62D}$ mutants sequester KCTD15$^{WT}$ in amyloid-like aggregates. The KCTD15$^{D104H}$ mutant sequesters KCTD1$^{WT}$ in amyloid-like aggregates. Scale bars, 10μm.

**Supplemental Figure 3: ACC-associated KCTD1 mutants sequester normal KCTD15 protein in amyloid-like aggregates and the ACC-associated KCTD15 mutant sequesters normal KCTD1 protein in amyloid-like aggregates in HeLa cells.**

KCTD1$^{WT}$ (green) and KCTD15$^{WT}$ (red) proteins show an overlapping cellular localization pattern in HeLa cells and do not form amyloid-like aggregates. In contrast, KCTD1$^{H74P}$ and KCTD1$^{G62D}$ mutants sequester KCTD15$^{WT}$ or KCTD1$^{WT}$ in amyloid-like aggregates. The KCTD15$^{D104H}$ mutant sequesters KCTD1$^{WT}$ in amyloid-like aggregates. Scale bars, 10μm.

**Supplemental Figure 4: Cellular localization of KCTD1 in vitro and in vivo.**

**A**, Left: KCTD1-eGFP transfection in HEK293 cells, HeLa cells, and HaCaT cells shows that KCTD1 can occur both in the nucleus and the cytoplasm, albeit it is mostly nuclear in HEK293 and HeLa cells and predominantly cytoplasmic in HaCaT cells. Scale bars, 10μm. Right: A subset of HaCaT cells shows strong nuclear localization as well. Scale bar, 100μm.

**B**, GFP transfection control for experiments in all three cell lines is shown. Scale bars, 10μm.

**C**, Immunolabeling for KCTD1 and KCTD15 in human skin shows mostly nuclear localization in keratinocytes (white arrow). CD68$^{+}$ dermal cells also stain for KCTD15 (yellow arrow). Scale bar, 50μm.

**D**, Mainly nuclear localization of KCTD15 in keratinocytes of WT mouse epidermis, which is not seen in *K14Cre$^{+}$Kctd15$^{fl/fl}$* mice but is maintained when AP-2α and AP-2β are inactivated. Scale bar, 50 μm.

**E**, Inactivation of KCTD1/KCTD15 in keratinocytes has no effect on nuclear localization of AP-2$\alpha$ and AP-2$\beta$. Scale bars, 50 $\mu$m.

**Supplemental Figure 5: X-gal labeling of tissues in *Kctd15$^{lacZ/WT}$* mice and in *Kctd1$^{lacZ/WT}$* mice.**

**A**, X-gal labeling of tissues from adult *Kctd15$^{LacZ/WT}$* and *Kctd1$^{LacZ/WT}$* reporter mice shows that both *Kctd1* and *Kctd15* are expressed in the skin epidermis (e.g., footpad skin) and hair follicles), or the tongue or esophagus epithelium. Footpad sweat gland epithelium shows expression of *Kctd1*, whereas sweat gland myoepithelial cells express *Kctd15*. Smooth muscle cells (e.g., of the tongue or esophagus) express *Kctd15* but not *Kctd1*. Ciliary body epithelium expresses *Kctd1* but not *Kctd15*. Scale bars, low magnification images 250$\mu$m, high magnification images 25$\mu$m.

**B**, *Kctd15* is expressed in the epithelium of bronchiae (black arrows) and surrounding smooth muscle (red arrows), whereas *Kctd1* is expressed only in the epithelium of bronchiae but not smooth muscle cells. *Kctd15* is expressed in bladder urothelium (black arrows) and bladder muscular layer (red arrow), whereas *Kctd1* is expressed only in urothelium but not smooth muscle cells. *Kctd15* is strongly expressed in colonic epithelium (black arrows), whereas *Kctd1* does not show a strong expression in colonic epithelium. Scale bars, low magnification images 250$\mu$m, high magnification images 25$\mu$m.

**C**, X-gal labeling of skin from E14.5 and E15.5 *Kctd15$^{LacZ/WT}$* and *Kctd1$^{LacZ/WT}$* reporter mice shows that both *Kctd1* and *Kctd15* are expressed in the developing epidermis. Scale bar sizes indicated in $\mu$m.

**D**, *Kctd1* is strongly expressed in the developing mouse heart at E14.5, particularly in the ventricular myocardium. *Kctd15* is strongly expressed in valves but not the ventricular myocardium of the developing mouse heart at E14.5. X-gal staining in *Kctd1$^{LacZ/WT}$* mice and *Kctd15$^{LacZ/WT}$* mice (blue color). Scale bars, 1mm.

**E**, Periocular mesenchyme shows expression of *Kctd1* and *Kctd15* at E14.5. X-gal staining in *Kctd1*$^{LacZ/WT}$ mice and *Kctd15*$^{LacZ/WT}$ mice (blue color). Scale bars, 500μm.

**F**, X-gal labeling in E8.5 *Kctd1*$^{LacZ/WT}$ mice and *Kctd15*$^{LacZ/WT}$ embryos.

**Supplemental Figure 6: *Kctd1* and *Kctd15* are both expressed in developing skin epidermis and in NCCs.**

**A**, Expression of *Kctd1* and *Kctd15* in the developing mouse skin based on scRNA-seq data (1).

**B**, Analysis of mouse scRNA-seq data (total 5,741 cells (2)) of neural crest cell populations shows an overlap in expression of *Kctd1* and *Kctd15* in neural crest cell populations. Expression of selected growth factors that stimulate keratinocyte migration/proliferation is shown as well.

**C**, Left: Analysis of scRNA-seq data from primate embryos (cynomolgus monkeys) shows an overlap in expression of *KCTD1* and *KCTD15* in both the surface ectoderm and in NCCs during embryonic development (total 56,636 cells (3)). Right: Expression of *KCTD1*, *KCTD15*, neural crest cell markers, and selected keratinocyte growth factors in the neural crest cell populations.

**Supplemental Figure 7: scRNA-seq data analysis shows higher *Kctd15* expression in cardiac neural crest cells (CNCC) and cardiac outflow tract during mouse development compared to *Kctd1*.**

**A**, Expression of *Kctd1*, *Kctd15*, and *Pax3* in CNCC-derived cells in mice from E10.5 to P7 (total 34,131 cells (4)). *Kctd15* shows a higher expression than *Kctd1* particularly in mural, mesenchymal, and neuronal cardiac NCC-derived cells.

**B**, Expression of *Kctd1, Kctd15*, and *Pax3* in both CNCC and non-CNCC-derived cells in mice between E8.5 to E10.5. *Kctd15* shows a higher expression than Kctd1 in all NCC-derived cells, including in the neural tube (total 4,651 cells (5)).

**C**, Interaction network of KCTD15, PAX3, and AP-2α generated by STRING.

**D**, Expression of *Kctd1*, *Kctd15* and *Pax3* in mouse outflow tract (OFT) dissected from three successive developmental stages corresponding to the early, middle, and late stages of OFT remodeling and septation. *Kctd15* is higher expressed than *Kctd1* in mesenchymal and epicardial cells of the OFT (total 55,611 cells (6)).

**E**, scRNA-seq expression in developing mouse hearts shown in Figure 12A (total 21,988 cells (7)). FHF: first heart field; SHF: second heart field; AHF: anterior heart field (from (7)).

PMIDs for the published datasets are shown.

**Supplemental Figure 8: scRNA-seq data analysis of *KCTD15* and *KCTD1* expression in the developing and adult human heart.**

**A**, In postnatal healthy human hearts, *KCTD1* is highly expressed in cardiomyocytes, cardiac neurons, and adipocytes, whereas *KCTD15* is expressed in endothelial and endocardial cells (total 54,260 cells (8)).

**B**, scRNA-seq data analysis from healthy human fetal hearts at day 83 gestational age shows that *KCTD15* is stronger expressed than *KCTD1* in endothelial and endocardial cells (total 13,569 cells (9)).

**C**, scRNA-seq data analysis from human fetal hearts at 6.5-7 post-conception weeks [PCW] shows that *KCTD15* is stronger expressed than *KCTD1* in endothelial cells and OFT (total 3,713 cells (10)).

PMIDs for the published datasets are shown.

**Supplemental Figure 9: KCTD1 but not KCTD15 is required for kidney function.**

**A**, Mouse scRNA-seq data show that *Kctd1* is strongly expressed in DCT epithelium, whereas *Kctd15* shows only low-level expression. From https://cello.shinyapps.io/kidneycellexplorer/. CT: connecting tubule; CD: collecting duct; PC: principal cell; IC: intercalated cell; LOH: loop of Henle.

**B**, Top and middle panels: Distinct non-overlapping expression pattern of *Kctd1* and *Kctd15* in the adult kidney. *Kctd15* is expressed mainly in medullary collecting ducts, whereas *Kctd1* is expressed in the distal nephron (medulla and cortex). X-gal staining in adult kidneys of *Kctd1*$^{Lacz/WT}$ and *Kctd15*$^{Lacz/WT}$ mice. In contrast to scRNA-seq data shown in A, no expression of *Kctd15* is detected in the glomeruli of the kidneys of *Kctd15*$^{Lacz/WT}$ mice. Related to Figure 11A. Lower panels: A distinct distribution of *Kctd1* and *Kctd15* expression patterns can already be observed at E14.5 in the developing kidney.

**C,** STRING interactions of DEGs in P8 kidneys from Six2*Cre*$^{+}$*Kctd1*$^{fl/fl}$ mice.

**Supplemental Figure 10: Inactivation of KCTD1 and KCTD15 in keratinocytes results in epidermal and hair follicle differentiation defects.**

**A**, A delay in hair growth and a postnatal growth retardation is observed in *K14Cre*$^{+}$*Kctd1*$^{fl/fl}$*Kctd15*$^{fl/fl}$ mice compared to WT littermates.

**B**, Three-week-old *K14Cre*$^{+}$*Kctd1*$^{fl/fl}$*Kctd15*$^{fl/fl}$ mice show sparse hair density and curly abnormal whiskers compared to control littermates.

**C**, RNA-seq data from 3-week-old back skin epidermis shows efficient K14Cre-mediated excision of floxed exon 3 of the *Kctd1* gene in *K14Cre*$^{+}$*Kctd1*$^{fl/fl}$ mice and of floxed exon 4 of the *Kctd15* gene in *K14Cre*$^{+}$*Kctd15*$^{fl/fl}$ mice, which results in the disruption of the critical BTB domain.

**D**, Normal distribution of Krt5 (basal cell layer) and loricrin (granular layer) in the skin of *K14Cre*$^{+}$*Kctd1*$^{fl/fl}$*Kctd15*$^{fl/fl}$ mice and WT controls. 4-week-old mice. No major difference in immunolabeling for the proliferation marker Ki67 or the transcription factors AP-2$\alpha$ and AP-2$\beta$ are detected. Scale bars, 50$\mu$m.

**E**, Top: No ectopically increased immunolabeling for Lef1 is found in the back skin epidermis of P24 *K14Cre*$^{+}$*Kctd1*$^{fl/fl}$*Kctd15*$^{fl/fl}$ mice. Bottom: Immunolabeling for collagen 17, keratin 5, and Ki67 in P4 WT and *K14Cre*$^{+}$*Kctd1*$^{fl/fl}$*Kctd15*$^{fl/fl}$ mouse skin. Scale bars, 100$\mu$m.

**F**, Expression of *Scd1* and *Elovl6* occurs mainly in sebaceous glands in mouse skin (based on (11)).

**G**, Expression of AP-2 transcription factors in keratinocytes does not depend on KCTD1 and/or KCTD15 in the epidermis and *vice versa*. RNA-seq data from 3-week-old back skin epidermis in control versus $K14Cre^{+}Kctd1^{fl/fl}Kctd15^{fl/fl}$ mice or $K14Cre^{+}Tfap2a^{fl/fl}Tfap2b^{fl/fl}$ mice.

**Supplemental Figure 11: ScRNA-seq data expression analysis of embryonic frontal mouse suture.**

**A**, Left: Expression of *Kctd1*, *Kctd15*, selected keratinocyte-promoting growth factors (*Fgf10, Igf2, Igf1, Fgf7, Tgfa), Tfap2a, Tfap2b*, and neural crest markers (*Pax3, Sox10*) in frontal suture cell populations of E16.5 and E18.5 mice (total 6,632 cells scRNA-seq (12)).

Right: Comparison of expression levels of IGF, FGF, and EGF signaling components in E16.5 and E18.5 mouse frontal suture cells.

FS: frontal suture clusters; DM: dura mater; HD: hypodermis; PC: pericytes; DC: dendritic cells; MP: macrophages; MO: monocytes; MC: mast cells; CEC: capillary endothelial cells.

**B**, The predicted incoming and outgoing relative strength of the signaling pathways in the HD cell population at E18.5.

**Supplemental Figure 12: Network structure and signaling strength of putative cell-cell communications in embryonic frontal mouse suture.**

**A**, Interaction analysis identifying signaling sources and targets at E16.5 and E18.5.

**B**, Heatmap depicting the cell-connectivity-summary networks based on mean expression weight at E16.5 and E18.5.

**C-E**, Chord diagrams of inferred IGF (C), EGF (D), and FGF (E) signaling networks from the various cell types to the HD (hypodermis) population at E18.5. Above subpanels show cell-cell interactions. Segments with large arrows represent signaling targets and inner bars represent signaling sources in which the colors indicate signaling targets. The thickness of each string

indicates the number of different interaction pairs colored by cell clusters. Below subpanels show inferred ligand-receptor interactions (total 6,632 cells (12)).

## TABLES

**Supplemental Table 1: Primary antibodies used in this study for immunofluorescence labeling.**

**Supplemental Table 2: PCR primers used in this study for semiquantitative RT-PCR.**

## SUPPLEMENTAL DATA FILES

**Supplemental Data, Excel File (Supplemental Table 3): RNA-seq expression data from P4 skin of WT and *K14Cre⁺Kctd1^fl/fl^Kctd15^fl/fl^* mice.**

**Supplemental Data, Excel File (Supplemental Table 4): RNA-seq expression data from 3-week-old epidermis of $K14Cre^{+}$ $Kctd15^{fl/fl}$ mice and $K14Cre^{+}Kctd1^{fl/fl}Kctd15^{fl/fl}$ mice.**

**Supplemental Movies: HaCaT cells transfected with KCTD1-eGFP.**

Images were taken every two hours for 48 hours (except the first 4 hours). Green: KCTD1-eGFP. Blue: DAPI (nuclear).

Movie 1: Green channel is shown (KCTD1-eGFP)

Movie 2: Green channel (KCTD1-eGFP) and blue channel (nuclear DAPI) are merged.

SUPPLEMENTAL REFERENCES

1. Jacob T, Annusver K, Czarnewski P, Dalessandri T, Kalk C, Levra Levron C, et al. Molecular and spatial landmarks of early mouse skin development. *Dev Cell.* 2023.
2. Kastriti ME, Faure L, Von Ahsen D, Bouderlique TG, Bostrom J, Solovieva T, et al. Schwann cell precursors represent a neural crest-like state with biased multipotency. *EMBO J.* 2022;41(17):e108780.
3. Zhai J, Guo J, Wan H, Qi L, Liu L, Xiao Z, et al. Primate gastrulation and early organogenesis at single-cell resolution. *Nature.* 2022;612(7941):732-8.
4. Chen W, Liu X, Li W, Shen H, Zeng Z, Yin K, et al. Single-cell transcriptomic landscape of cardiac neural crest cell derivatives during development. *EMBO Rep.* 2021;22(11):e52389.
5. Soldatov R, Kaucka M, Kastriti ME, Petersen J, Chontorotzea T, Englmaier L, et al. Spatiotemporal structure of cell fate decisions in murine neural crest. *Science.* 2019;364(6444).
6. Liu X, Chen W, Li W, Li Y, Priest JR, Zhou B, et al. Single-Cell RNA-Seq of the Developing Cardiac Outflow Tract Reveals Convergent Development of the Vascular Smooth Muscle Cells. *Cell Rep.* 2019;28(5):1346-61 e4.
7. de Soysa TY, Ranade SS, Okawa S, Ravichandran S, Huang Y, Salunga HT, et al. Single-cell analysis of cardiogenesis reveals basis for organ-level developmental defects. *Nature.* 2019;572(7767):120-4.
8. Hill MC, Kadow ZA, Long H, Morikawa Y, Martin TJ, Birks EJ, et al. Integrated multi-omic characterization of congenital heart disease. *Nature.* 2022;608(7921):181-91.
9. Miao Y, Tian L, Martin M, Paige SL, Galdos FX, Li J, et al. Intrinsic Endocardial Defects Contribute to Hypoplastic Left Heart Syndrome. *Cell Stem Cell.* 2020;27(4):574-89 e8.
10. Asp M, Giacomello S, Larsson L, Wu C, Furth D, Qian X, et al. A Spatiotemporal Organ-Wide Gene Expression and Cell Atlas of the Developing Human Heart. *Cell.* 2019;179(7):1647-60 e19.

11. Joost S, Annusver K, Jacob T, Sun X, Dalessandri T, Sivan U, et al. The Molecular Anatomy of Mouse Skin during Hair Growth and Rest. *Cell Stem Cell.* 2020;26(3):441-57 e7.

12. Holmes G, Gonzalez-Reiche AS, Lu N, Zhou X, Rivera J, Kriti D, et al. Integrated Transcriptome and Network Analysis Reveals Spatiotemporal Dynamics of Calvarial Suturogenesis. *Cell Rep.* 2020;32(1):107871.

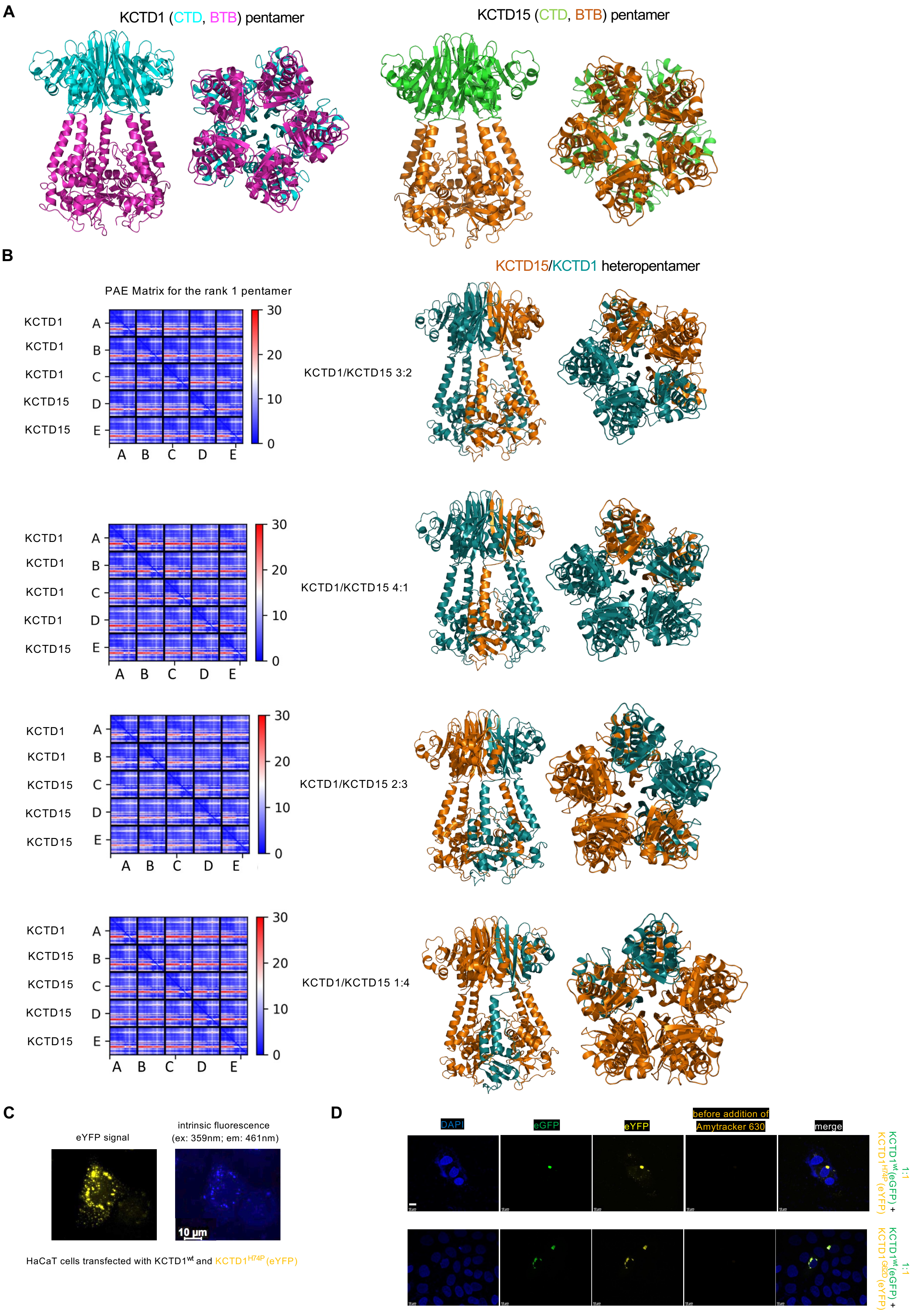


**Supplemental Figure 1**

HaCaT cells

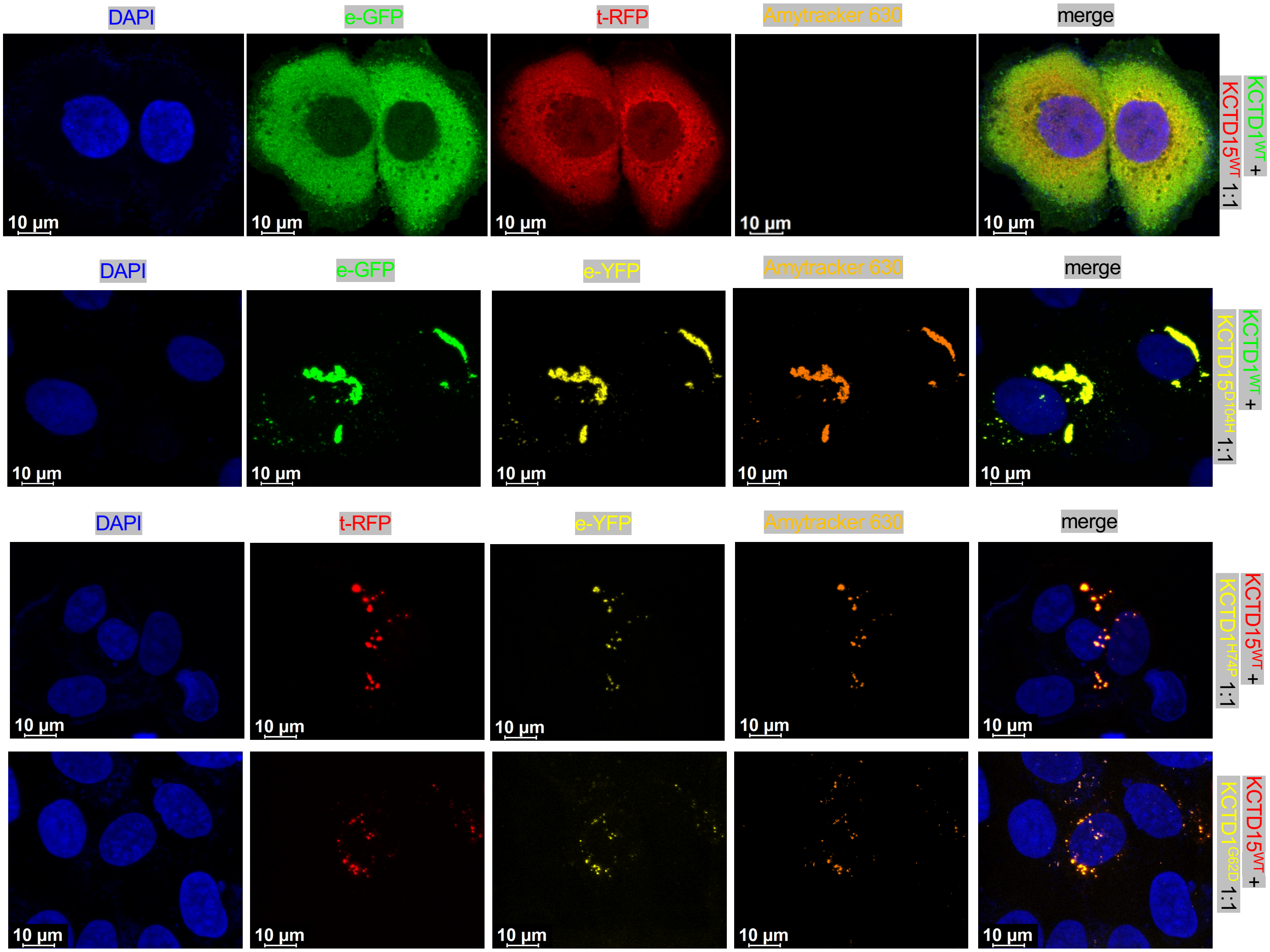


**Supplemental Figure 2**

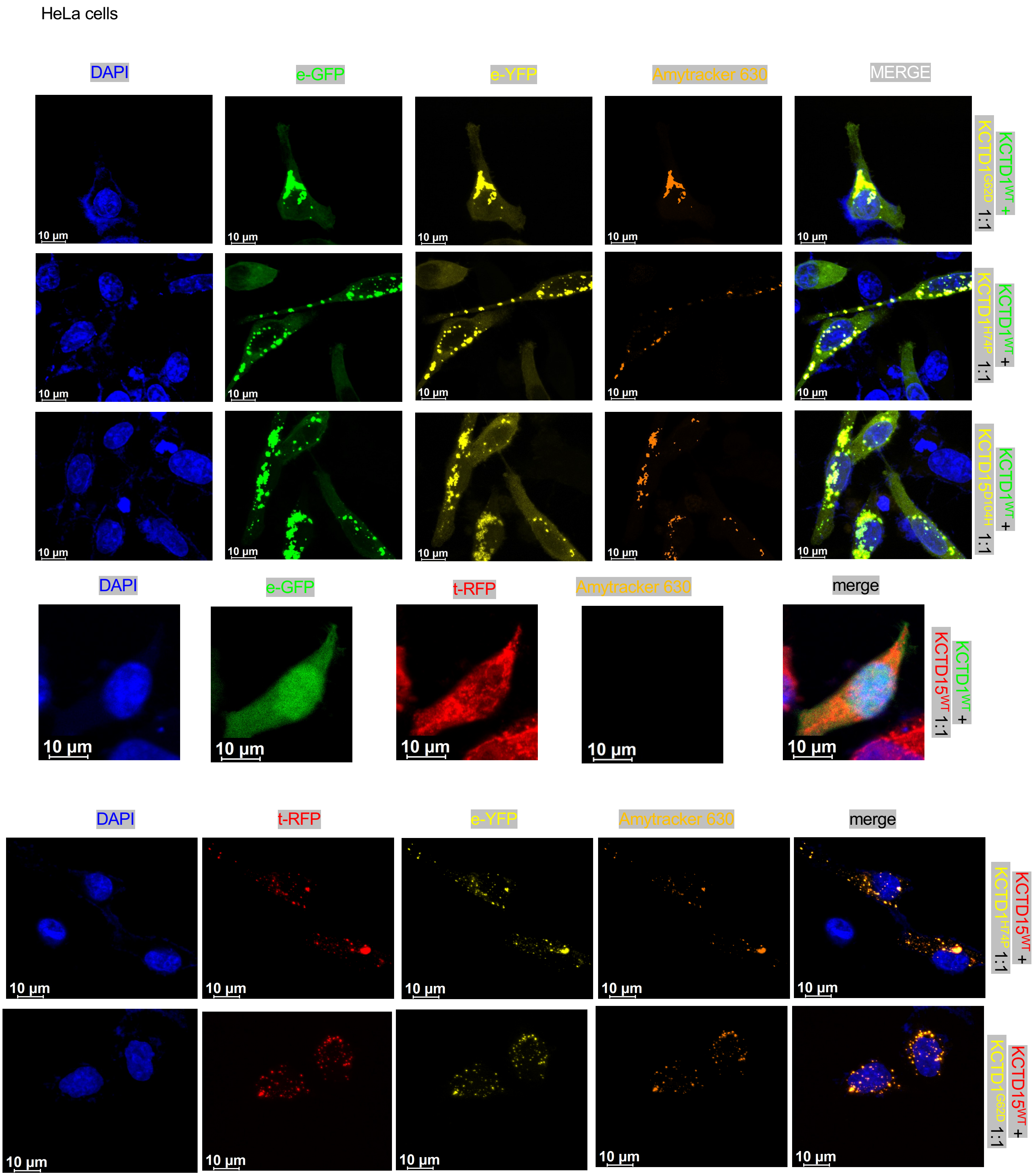


**Supplemental Figure 3**

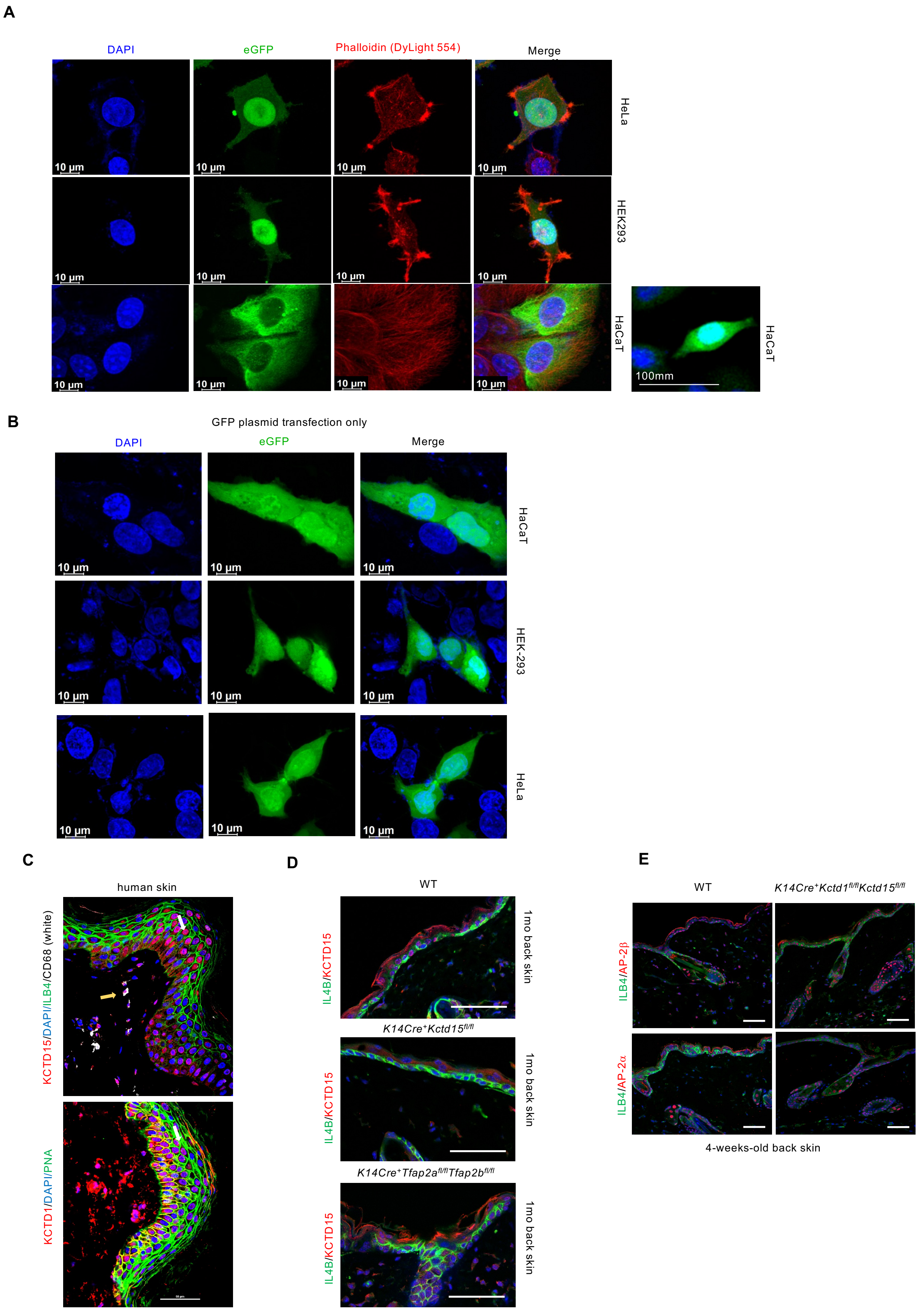


**Supplemental Figure 4**

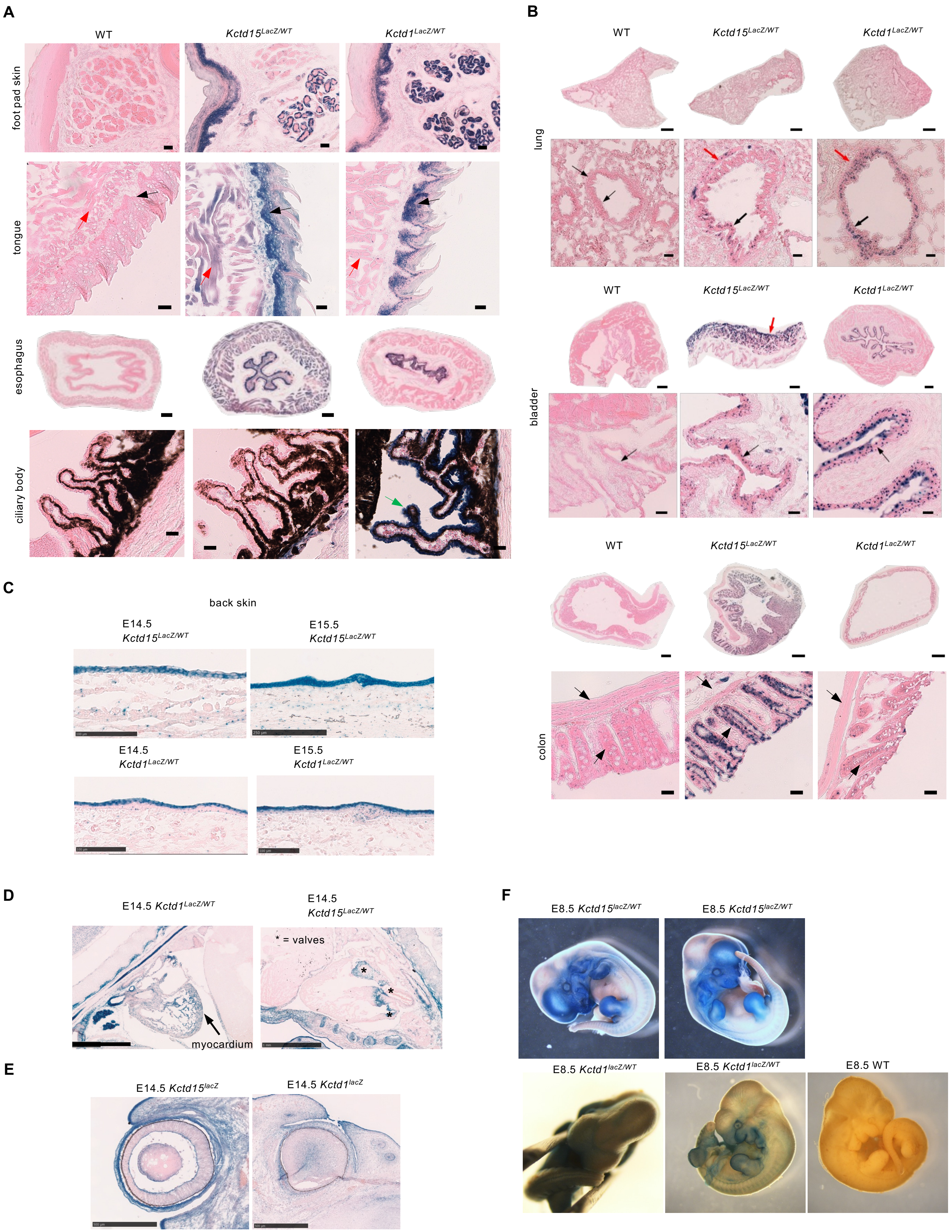


# Supplemental Figure 5

**A**

scRNA-Seq data: mouse skin development

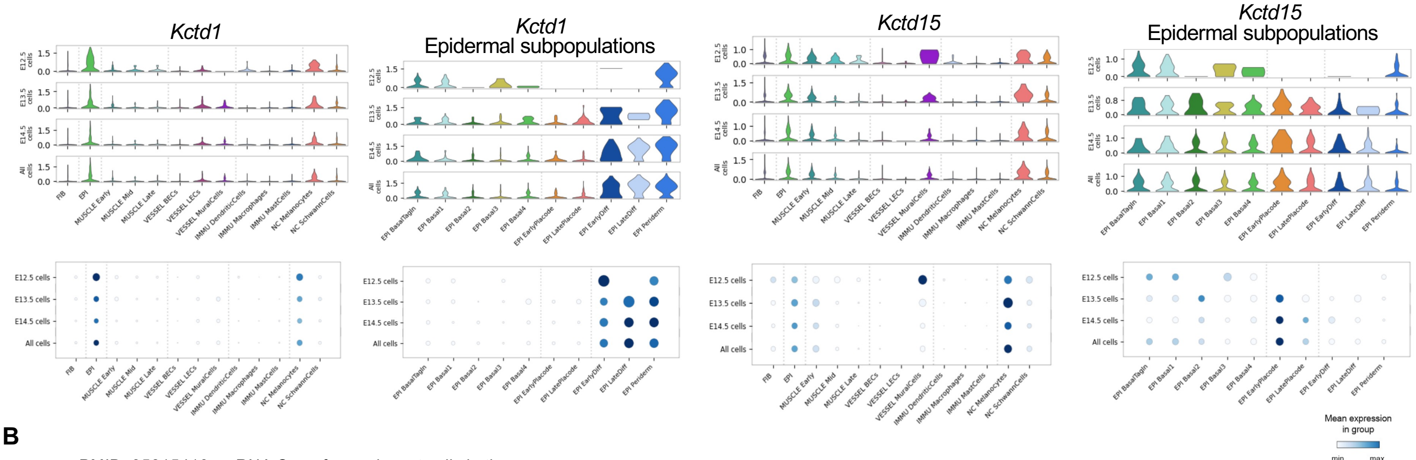


**B**

PMID: 35815410: scRNA Seq of neural crest cells in the mouse

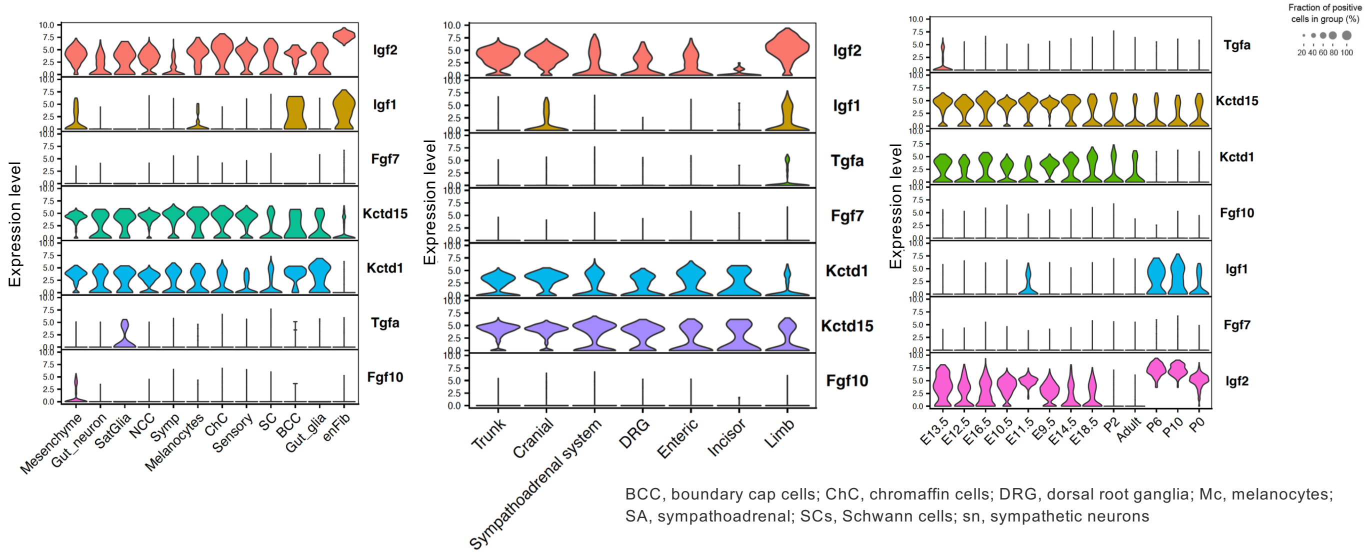


BCC, boundary cap cells; ChC, chromaffin cells; DRG, dorsal root ganglia; Mc, melanocytes; SA, sympathoadrenal; SCs, Schwann cells; sn, sympathetic neurons

**C**

scRNA-Seq data: Carnegie stage 8–11 cynomolgus monkey (*Macaca fascicularis*) embryos

Zhai J, Guo J, Wan H, Qi L et al. Primate gastrulation and early organogenesis at single-cell resolution. Nature 2022 Dec;612(7941):732-738. PMID: 36517595 (GSE193007)

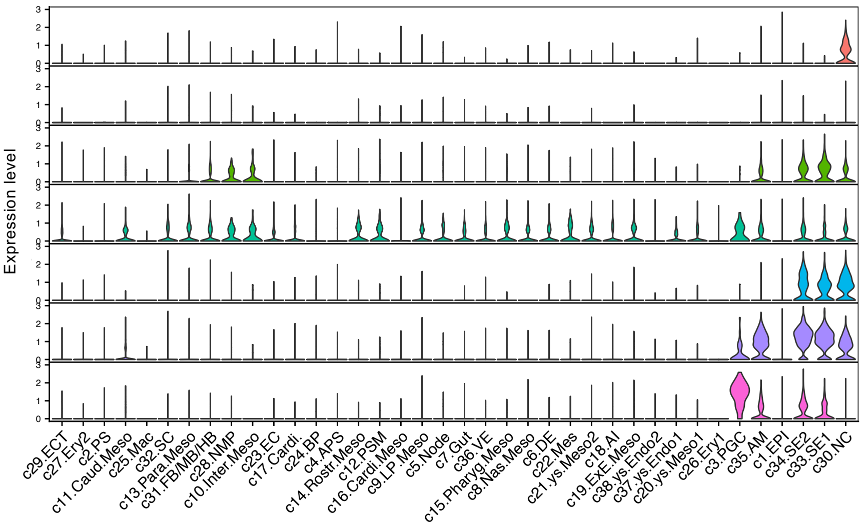


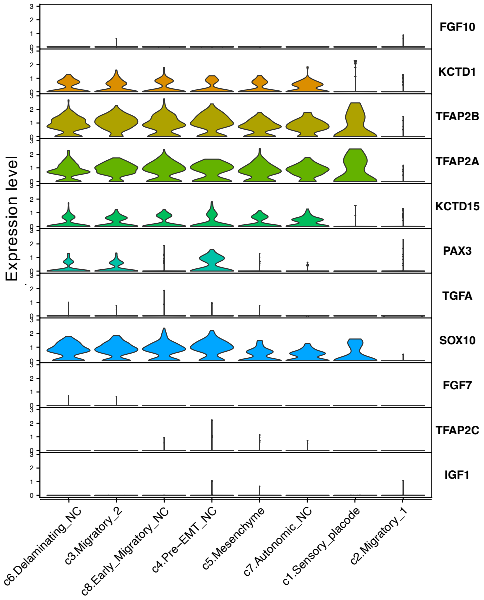


KCTD1: NCC, surface ectoderm, neuromesodermal progenitor, intermediate mesoderm, brain, amnion

KCTD15: NCC, surface ectoderm, primordial germ cells, neuromesodermal progenitor, …

EPI, epiblast; Mes, mesenchyme; EC, endothelial cell; BP, blood progenitor; Mac, macrophage; Ery, erythrocyte; ys.Endo, yolk sac endoderm. ys, yolk sac; Al, allantois; AM, amnion; FB, forebrain; Cardi., cardiac. PS: primitive streak; EPI: epiblast; PS (no. 2), anterior primitive streak (APS, no. 4), definitive endoderm (DE, no. 6), node (no. 5) and nascent mesoderm (Nas.Meso, no. 8), Nas.Meso derivatives, including intermediate (Inter.Meso), paraxial (Para.Meso), rostral (Rostr.Meso), pharyngeal (Pharyn.Meso), cardiac (Cardi.Meso), lateral plate (LP.Meso), caudal (Caud.Meso) mesoderm cells and extra-embryonic mesenchymal cells (EXMCs, including allantois (Al), yolk sac (ys.Meso) and extra-embryonic mesoderm cells (ExE.Meso), neuromesodermal progenitor (NMP) and presomitic mesoderm (PSM) cells, extra-embryonic mesenchymal cells (EXMCs, including ys.Meso, ExE.Meso, some mesenchyme, among others), neural ectoderm (NE, also called neural plate), surface ectoderm (SE, cluster nos. 33 and 34) at the periphery and neural plate border (NPB) between the two, which ventrally delaminates and differentiates into neural crest (NC, cluster no. 30); SC: spinal cord; primordial germ cells (PGCs), ectoderm (NE, FB/MB/HB, SE, SC and NC), mesoderm (node, Nas.Meso, Inter.Meso, Para.Meso, PSM, NMP) and endoderm (VE, DE and Gut)

**Supplemental Figure 6**

**A**

*Wnt1-Cre;Rosa26-tdTomato* mouse: cardiac NCC-derived cells (E10.5-P7)
PMID: 34569705

*Kctd15>Kctd1* in mural, mesenchymal and neuronal cardiac NCCs

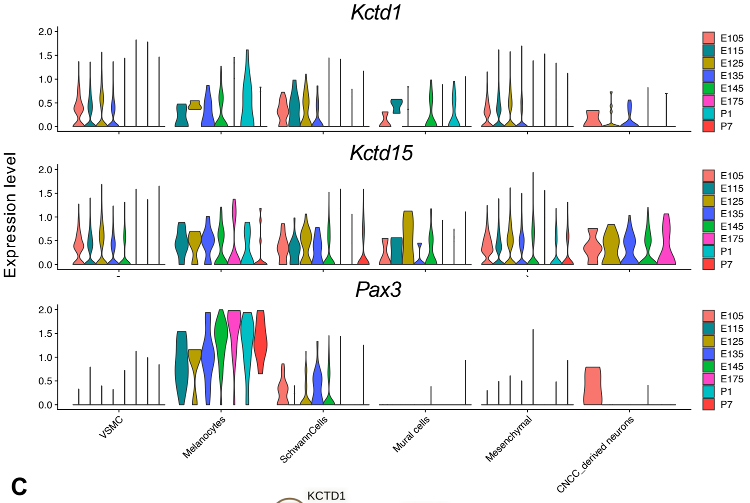


**B**

*Wnt1-Cre;Rosa26-tdTomato* mouse: neural crest cells from mouse embryos (E8.5-E10.5)
PMID: 31171666

*Kctd15>Kctd1* in all cardiac NCCs, including in the neural tube

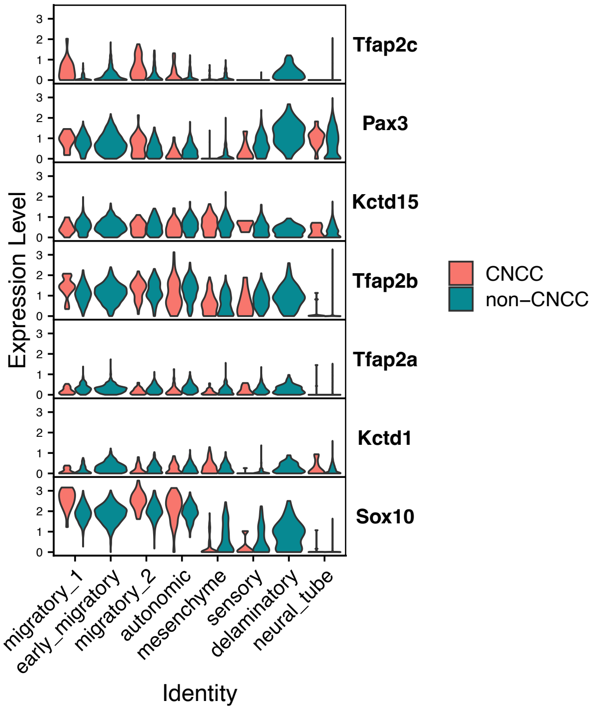


**C**

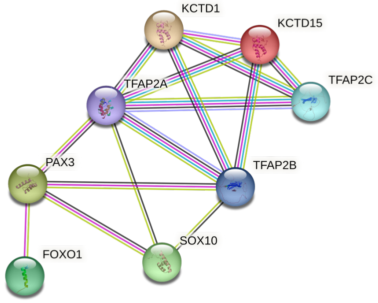


**D**

mouse OFT dissected from three successive developmental stages corresponding to the early, middle, and late stages of OFT remodeling and septation
PMID: 31365875

*Kctd15>Kctd1* in mesenchymal (C0, C1, C7, C8) and epicardial cells of the OFT

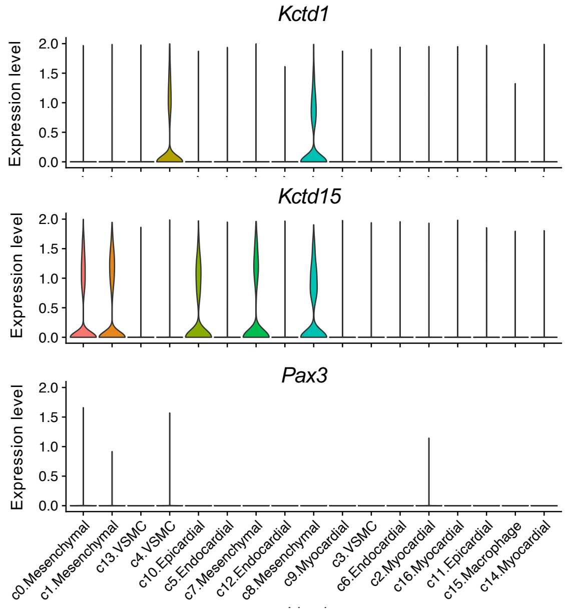


**E**

developing mouse hearts at E7.75, E8.25 and E9.25
image from PMID: 31341279

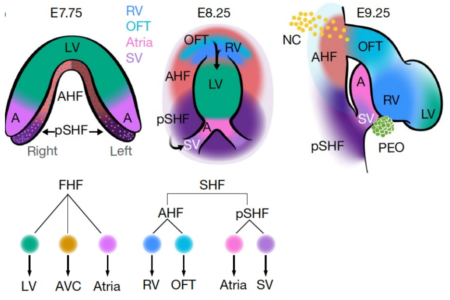


EMP: early myocardium progenitors
SV: sinus venosus
OFT: outflow tract
AVC: atrioventricular canal

**Supplemental Figure 7**

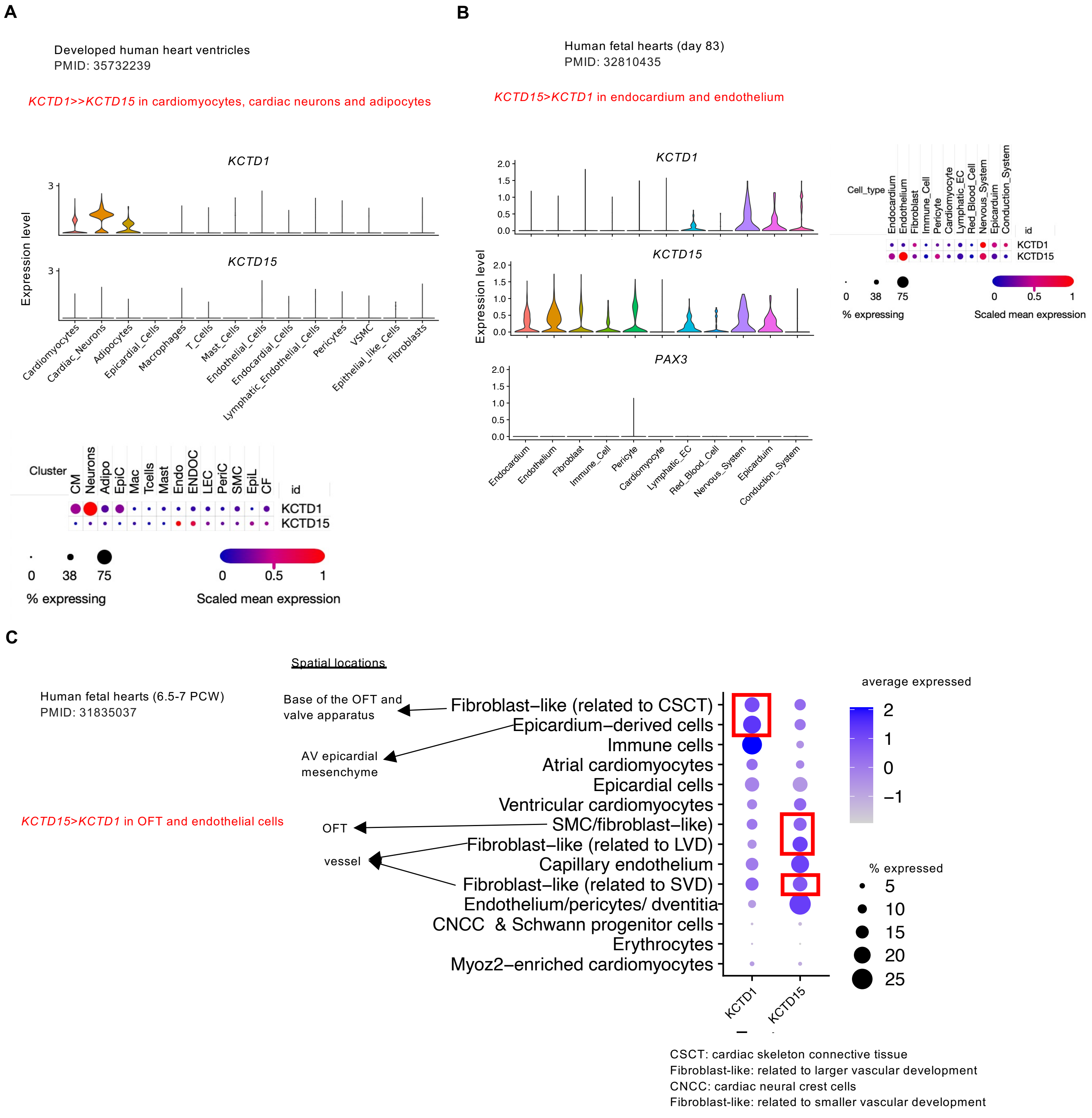


# Supplemental Figure 8

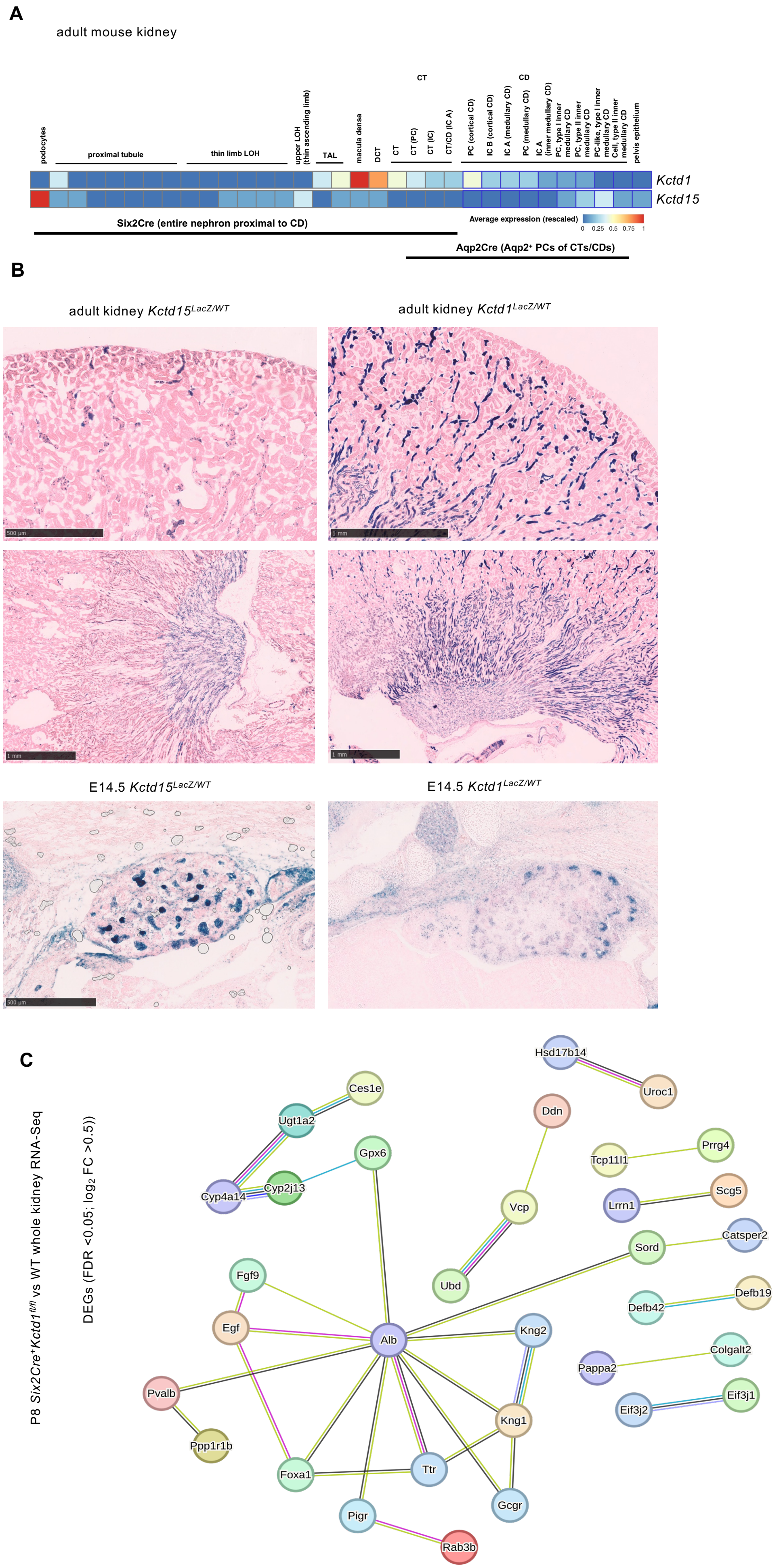


**Supplemental Figure 9**

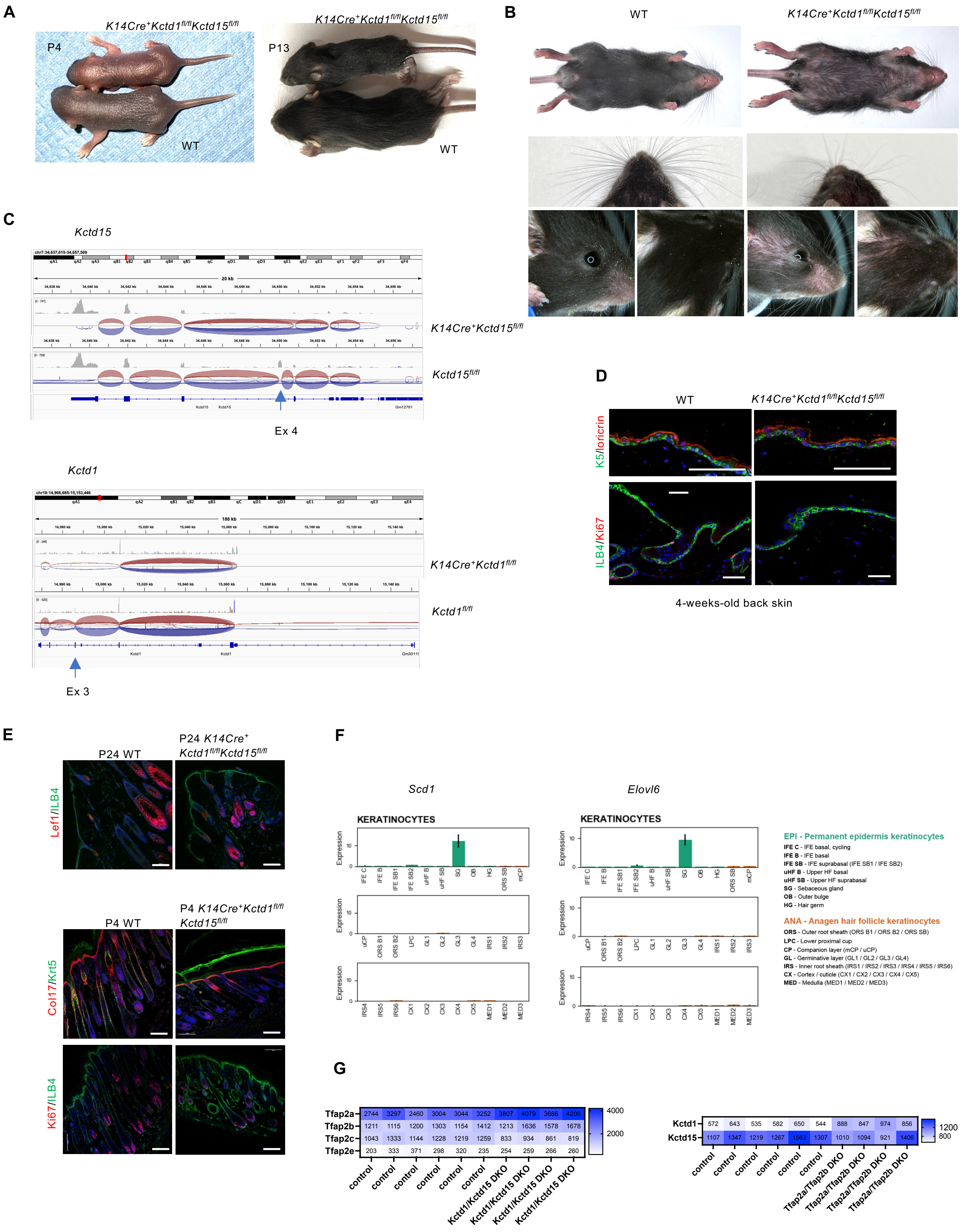


# Supplemental Figure 10

A

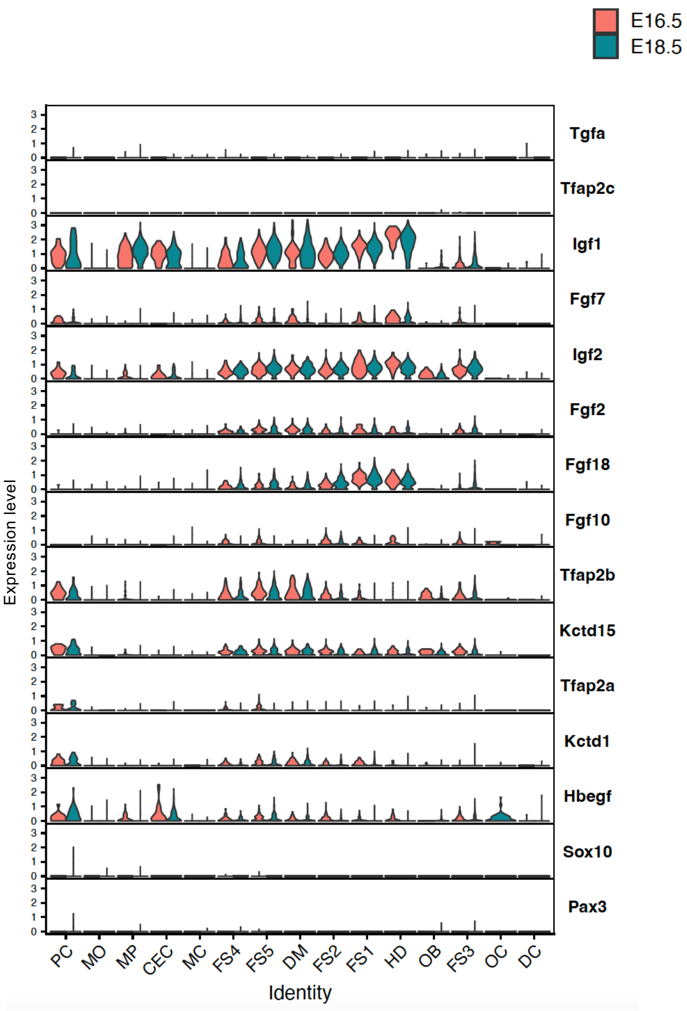


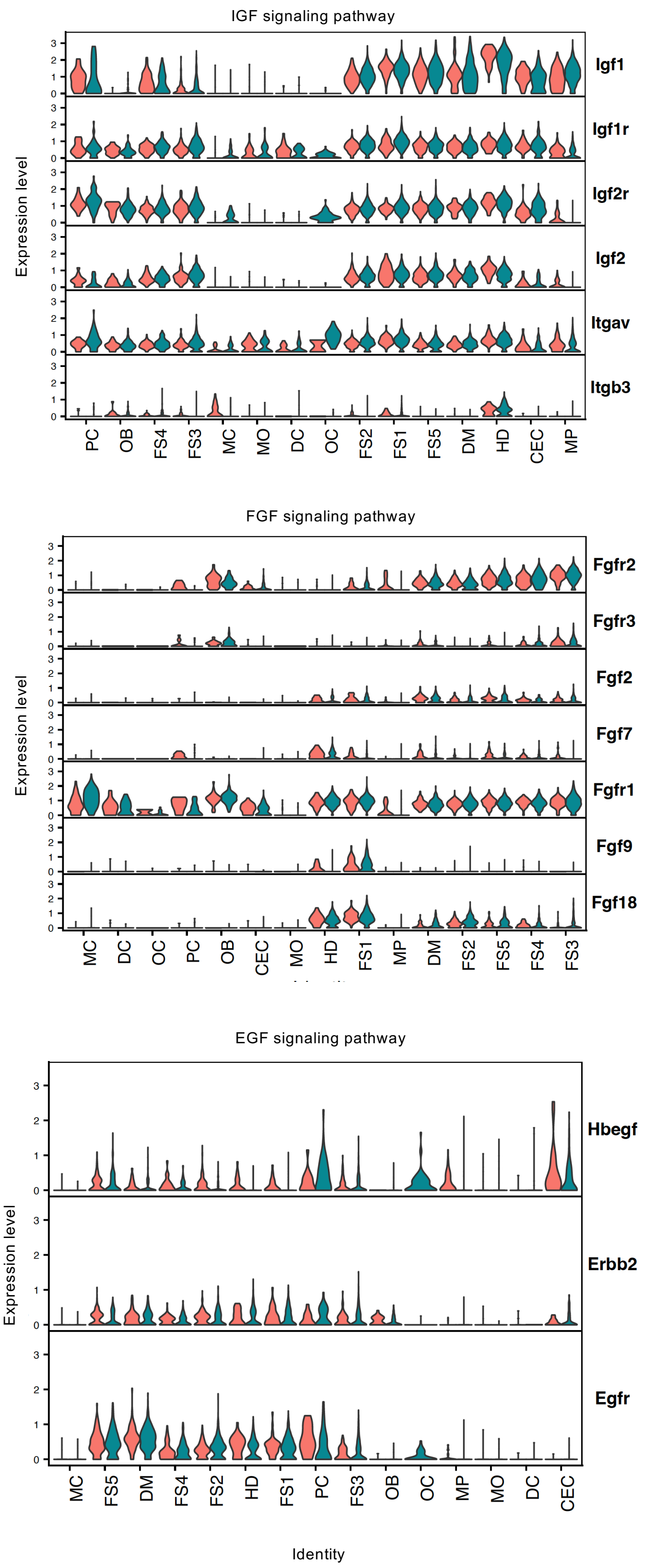


B

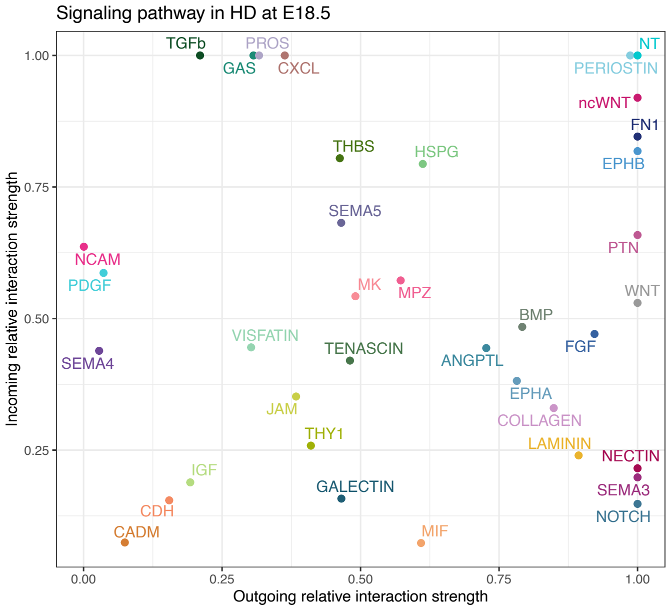


**Supplemental Figure 11**

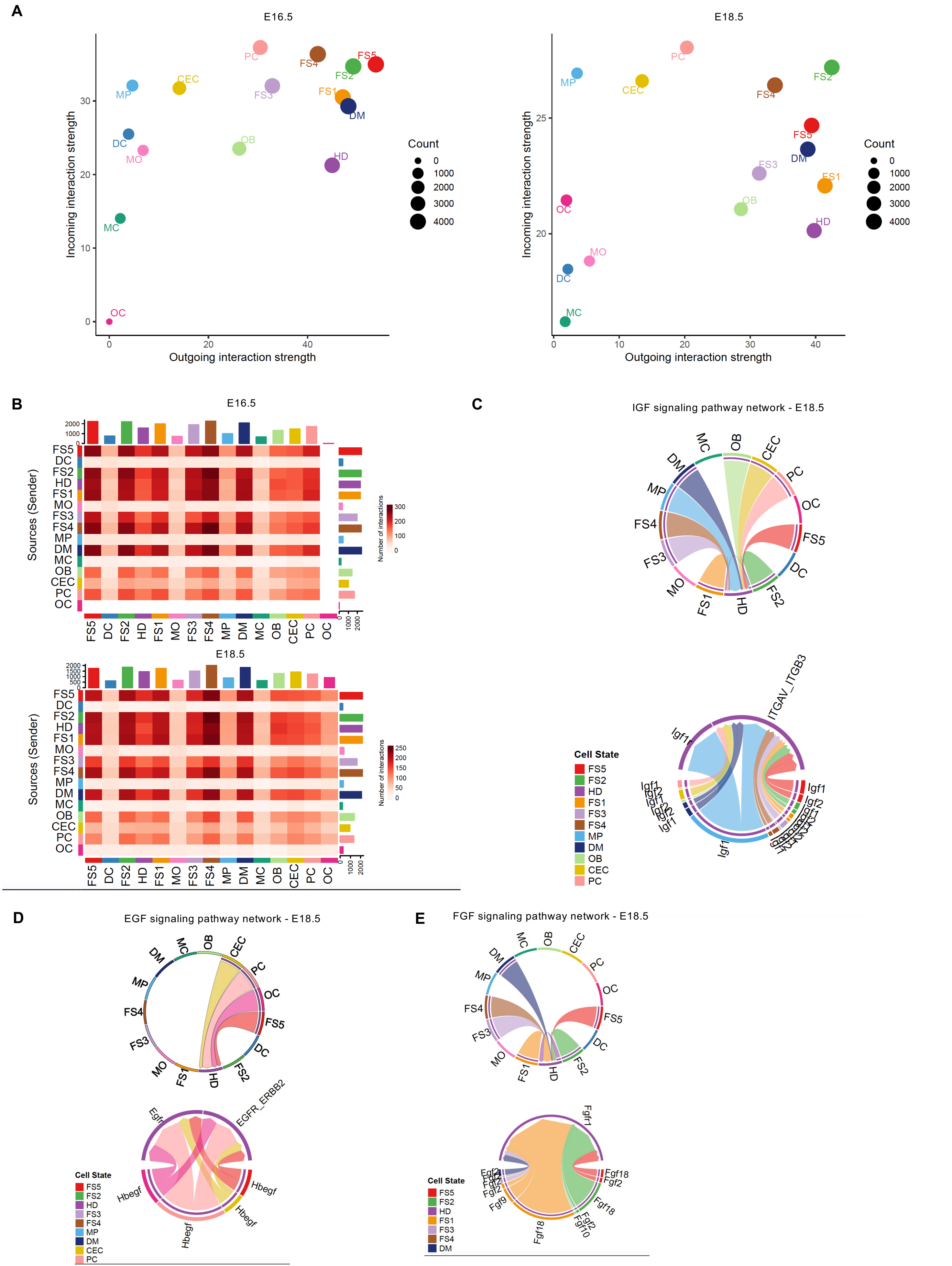


**Supplemental Figure 12**
