# Supplementary material for: KCTD1/KCTD15 complexes control ectodermal and neural crest cell functions, and their impairment causes aplasia cutis
Source: J Clin Invest. 2023 Dec 19;134(4):e174138. doi: 10.1172/JCI174138 (PMC10866662; doi:10.1172/JCI174138)
Supplement: Supplemental tables 1-2 [file jci-134-174138-s080.pdf]

Supplemental Table 1: Primary antibodies used in this study for immunofluorescence labeling.

rabbit anti-AP-2β (1:200)	Cell Signaling Technology	Cat# 2509, RRID:AB_2058198
guinea pig anti-Krt5 (1:200)	American Research Products	03-GP-CK5, RRID:AB_1541051
rabbit anti-Krt6a (1:500)	Biolegend	905701, RRID:AB_2565052
Foxi3 (mouse monoclonal, clone N359/28) (1:100)	NeuroMab	RRID: AB_2315836
rabbit anti-Col17 (1:100)	Abcam	ab184996 RRID:AB_3073488
rabbit anti-Melan-A (1:400)	Abcam	ab210546, RRID:AB_2889292
rabbit anti-Lef1 (1:100)	Cell Signaling Technology	2230S, RRID:AB_823558
rabbit anti-Ki67 (1:100)	Abcam	ab16667, RRID:AB_302459
rabbit anti-loricrin (1:200)	Abcam	ab85679, RRID:AB_2134912
rabbit anti-AP-2α (1:200)	Abcam	ab108311, RRID:AB_10861200
rabbit anti-β3 tubulin (1:50)	Cell Signaling Technology	5666S, RRID:AB_10691594
rabbit anti-GFP (1:100)	Thermo Fisher Scientific	Cat# A-11122, RRID:AB_221569
rabbit anti-active β-catenin (non-phospho Ser33/37/Thr41) (1:100)	Cell Signaling Technology	Cat# 8814S, RRID:AB_11127203
rabbit anti-SCD1 (C12H5) antibody (1:100)	Cell Signaling Technology	Cat# 2794S, RRID:AB_2183099
mouse anti-CD68 (1:100)	Thermo Fisher Scientific	Cat# 14-0688-82, RRID:AB_11151139
rabbit anti-KCTD15 (1:100)	Abcam	Cat# ab106373, RRID:AB_10859595
rabbit anti-KCTD1 (1:100)	Sigma	Cat# SAB1301602

Supplemental Table 2: PCR primers used in this study for semiquantitative RT-PCR.

**Primer Name**	**Sequence (5' to 3')**
mKCTD15-up1	TCCTCGCTTAACGCACACG
mKCTD15-dw1	AGACGGCTTATTCTGGAGTCA
mPax3-up1	CCGGGGCAGAATTACCCAC
mPax3-dw1	GCCGTTGATAAATACTCCTCCG
m-Pax3-up2	TACCAGCCCACGTCTATTCC
m-Pax3-dw2	TAGCCTGCGGTGCTATAGGT
mKctd15-up2	TCCTCGCTTAACGCACACG
mKctd15-dw2	AGACGGCTTATTCTGGAGTCA
mKCTD15-up3	GTTTCCCTCTCAACGGCTAC
mKCTD15-dw3	CGGCAAAGAACATATTCGCTG
m36B4 up	TCACTGTGCCAGCTCAGAAC
m36B4 dw	AATTTCAATGGTGCCTCTGG
